# Gene Size Matters: An Analysis of Gene Length in the Human Genome

**DOI:** 10.3389/fgene.2021.559998

**Published:** 2021-02-11

**Authors:** Inês Lopes, Gulam Altab, Priyanka Raina, João Pedro de Magalhães

**Affiliations:** Integrative Genomics of Ageing Group, Institute of Ageing and Chronic Disease, University of Liverpool, Liverpool, United Kingdom

**Keywords:** genomics, transcripts, gene expression, immune system, mRNA, SNPs

## Abstract

While it is expected for gene length to be associated with factors such as intron number and evolutionary conservation, we are yet to understand the connections between gene length and function in the human genome. In this study, we show that, as expected, there is a strong positive correlation between gene length, transcript length, and protein size as well as a correlation with the number of genetic variants and introns. Among tissue-specific genes, we find that the longest transcripts tend to be expressed in the blood vessels, nerves, thyroid, cervix uteri, and the brain, while the smallest transcripts tend to be expressed in the pancreas, skin, stomach, vagina, and testis. We report, as shown previously, that natural selection suppresses changes for genes with longer transcripts and promotes changes for genes with smaller transcripts. We also observe that genes with longer transcripts tend to have a higher number of co-expressed genes and protein-protein interactions, as well as more associated publications. In the functional analysis, we show that bigger transcripts are often associated with neuronal development, while smaller transcripts tend to play roles in skin development and in the immune system. Furthermore, pathways related to cancer, neurons, and heart diseases tend to have genes with longer transcripts, with smaller transcripts being present in pathways related to immune responses and neurodegenerative diseases. Based on our results, we hypothesize that longer genes tend to be associated with functions that are important in the early development stages, while smaller genes tend to play a role in functions that are important throughout the whole life, like the immune system, which requires fast responses.

## Introduction

With the sequencing of the human genome (Lander et al., 2001; Venter et al., 2001; International Human Genome Sequencing Consortium, 2004) arose a great interest in understanding the relationship between genotype and phenotype, especially concerning human health (Gonzaga-Jauregui et al., 2012; Goldfeder et al., 2017). However, despite the recent advancements, we have yet to fully understand the human genome and its complexity (Simonti and Capra, 2015).

Several studies have tried to decipher the connections between the length of a gene and its functions. It is believed that genes that are more evolutionarily conserved are often associated with longer gene length and higher intronic burden (Wolf et al., 2009; Vishnoi et al., 2010; Gorlova et al., 2014; Grishkevich and Yanai, 2014). In contrast, a shorter gene length is associated with a high expression, smaller proteins, and little intronic content (Urrutia and Hurst, 2003). This hypothesis is further supported by housekeeping genes, which are widely expressed and have characteristics similar to genes with shorter length (Eisenberg and Levanon, 2003). It was hypothesized that, due to the great levels of expression in smaller genes, there is selective pressure to maximize protein synthesis efficiency (Urrutia and Hurst, 2003). If that is the case, then the question remains regarding which functions benefit longer genes to compensate for their more expensive production of proteins.

Gene length has been associated with biological timing. In response to stimuli, smaller genes produce proteins faster, and these smaller proteins often play a part in the regulation of longer proteins, which, in turn, are expressed later in the response. This allows for regulatory mechanisms to be set up in preparation for the expression of important proteins (Kirkconnell et al., 2017). Indeed, longer genes have been associated with important biological processes, including embryonic development (Yang et al., 2018) and neuronal processes (Sahakyan and Balasubramanian, 2016). Longer genes have also been previously shown to be related to diseases such as cancer, cardiomyopathies, and diabetes (Sahakyan and Balasubramanian, 2016).

In this work, we used human genome data to identify possible functions associated with gene size, with a focus on protein-coding regions and genes. Correlation tests were used to identify relationships between gene length and other gene and protein characteristics. We observed that longer genes are expressed in the brain, heart diseases, and cancer, while smaller genes mostly participate in the immune system and in the development of the skin. Therefore, we hypothesize, based on our results, that genes with longer transcripts are mostly associated with functions in the early development stages, while genes with smaller transcripts have important roles in day-to-day functions.

## Materials and Methods

### Data Retrieval and Filtering

All protein-coding human transcripts and genes (*N*_transcripts_ = 92,696), their length, transcript count, and GC content were obtained using the BioMart (Zerbino et al., 2018) website (GRCh38.p12, Ensembl 96, April 2019). Transcript length is defined by Ensembl as the total length of the exons in a gene plus the lengths of its untranslated (UTR) regions. Gene length was obtained using the R (version 3.5.2) packages biomart (version 2.38.0) and GenomicRanges (version 1.34.0) and based on the code for the getGeneLengthAndGCContent function of the EDASeq (version 2.14.1) package. To avoid any discrepancies between how Ensembl defines transcript length and our calculated gene length, we extracted the start and end positions for all exons+UTR regions of all transcripts and calculated gene length based on the combined length between those regions. Using R, the transcripts with the highest transcript length per gene were selected. In the case of ties, due to multiple transcripts having the same length per gene, we used some tags (APPRIS annotation was the principal one, if there was an entry in RefSeq or GENCODE) used by Ensembl as a tiebreaker. Should that fail, the oldest transcript was chosen, by means of having a smaller numerical ID. Transcripts associated with PATCH locations or assemblies were removed from our dataset. For each transcript, we obtained data regarding their number of exons, coding sequence (CDS) length, the number of single nucleotide polymorphisms (SNPs), synonymous (“synonymous_variant”), missense (“missense_variant“), and nonsense (“stop_gained”) SNPs, protein length, and the dN and dS values using the biomart package. For the dN and dS values, only those associated with one-to-one orthologs were selected for the present analysis. Average expression was obtained from the USCS Table browser tool (Karolchik et al., 2004) using expression as the group and the GTEx Gene track. Tissue-specific tau values of expression were obtained from another work (Palmer et al., 2019). The number of SNPs per gene was obtained using the biomart package and website.

The whole file produced and used in the analysis for this work can be found in Supplementary Table S1 (*N* = 19,714).

A second dataset was built using the APPRIS annotation (Rodriguez et al., 2013) provided by Ensembl. Transcripts were first selected for each gene, based on whether they were identified as the principal isoform. Transcripts with entries in RefSeq were used as a tiebreaker. Should there still be duplicates, the oldest transcript was used. This dataset can be found in Supplementary Table S2A (*N* = 19,702).

Genes related with aging (*N* = 307) were obtained from GenAge (Build 19; Tacutu et al., 2018).

### Statistical Tests, Graphs, and Other Packages

R and the function corr.test were used to perform the correlation tests. Due to the abundance of data, there were a lot of ties in the ranks, which prevented the usage of Spearman’s correlation, so instead we chose to use the Kendall test for the correlations. Partial correlations were done using the ppcor (version 1.1) package. For multivariable regression, the lm function in R was used based on the following formula: Number of PPI ~ Transcript Length + Number of publications. The figures produced in this work were created using the ggplot2 (version 3.2.0) package in R. The other packages used over the course of this work were: corrplot (version 0.84), psych (version 1.9.12.31), ggpubr (version 0.2.1), cowplot (version 1.0.0), stringr (version 1.4.0), dplyr (version 0.8.5), plyr (version 1.8.4), and tidyr (version 0.8.3).

### Functional Analysis

WebGestalt (2019 release; Liao et al., 2019) was used to do the overrepresentation enrichment analysis for each of the Gene Ontology (GO) categories (biological process, cellular component, and molecular function). The top 5% genes, with the highest and the lowest gene lengths, were ran against the reference option of genome. The significance level was FDR < 0.05 and the multiple test adjustment was done using the Benjamini-Hochberg method.

For confirmation of the results, the same two 5% lists were run on DAVID’s (Huang et al., 2009a,b) annotation clustering option using the complete human genome as background. Only those terms with a value of *p* and false discovery rate (FDR) smaller or equal to 0.05 were considered. Default categories were used, except for the category “UP_SEQ_FEATURE” since it was introducing a lot of redundant results.

To help better visualize the GO terms obtained from the analysis described above, the tool REViGO (Supek et al., 2011) was used. The values of *p* here considered were the FDR values obtained previously, with the human database option used for the GO terms.

With regard to the analysis done using the Kyoto Encyclopedia of Genes and Genomes (KEGG) pathways, the grouping of genes with pathways was obtained from the Molecular Signature database (version 6.2; Kanehisa and Goto, 2000; Subramanian et al., 2005; Liberzon et al., 2011, 2015; Kanehisa et al., 2017, 2019), as was done previously by another group (Sahakyan and Balasubramanian, 2016). For each gene, the length used was that of the corresponding transcript from our dataset. Additionally, the coloring of the box plot was done based on the fact that the pathway in question is directly associated with the category (when the KEGG pathway schematic shows cells from the category) or if they could be indirectly associated with the category (using available literature). For this latter case, the appropriate literature was selected if the said literature mentioned elements of the KEGG pathway being involved in the said category. The categorization on the basis of published work has its advantages, but there is often overlapping of functions within these categories; for example, calcium signaling also happens in the muscle (Kuo and Ehrlich, 2015) and immune system (Vig and Kinet, 2009), the Wnt signaling pathway also has a role in cancer (Zhan et al., 2017), and the TGF-beta signaling pathway can also be associated with the immune system (Worthington et al., 2012), among others.

While the KEGG pathways used in this work did not incorporate all of the genes in our dataset, these KEGG pathways were considered canonical by the MSigDB (Subramanian et al., 2005; Liberzon et al., 2015), which would provide more certainty that our results are genuine by removing a lot of the ambiguity around the existence of several of our genes.

To further our understanding of the influence of gene length in the immune system, we analyzed data from Reactome (version 75; Jassal et al., 2019). Data were extracted based on the ontology levels. If we assume that the immune system is the first ontology level, then the second level would include its child terms (innate immune system, adaptive immune system, and cytokine signaling), with the third level including the child terms of the second level, and so on.

### Co-Expression Analysis

Co-expression correlation values were extracted from GeneFriends (van Dam et al., 2015). For each gene (*N* = 19,714), in the whole dataset and in the top 5% lists of genes with the longest (High group) and the smallest (Low group) transcript lengths (*N* = 986 for each list), the number of genes with correlation values superior or equal to 0.6 or smaller or equal to −0.6 were obtained using R. From our original dataset (*N* = 19,714 genes), 1,046 genes were not present in GeneFriends (whole dataset), of which 25 missing genes were within the High group and 110 missing genes were within the Low group.

For obtaining the median values of genes present in the GeneFriends database, the co-expression values for each gene across the database were merged, and this was followed by the calculation of median values using R.

### Protein-Protein Interaction Analysis

BioGRID (release 3.5.174) REST API (Stark, 2006), in combination with the R package httr (version 1.4.0), was used to obtain all protein-protein interactions for the whole dataset and for the top 5% longest and smallest genes. All redundant and genetic interactions were removed from this analysis.

For the publication bias analysis, the number of publications, in PubMed, per gene of each group were obtained using the Entrez Programming Utilities (E-utilities) and the R packages XML (version 3.98-1.19), httr, and biomart.

## Results

### Longest and Shortest Genes

From all of the protein-coding genes in the human genome, a dataset was built selecting only the longest transcripts for each gene (*N* = 19,714 genes; Supplementary Table S1). As our focus is on protein-coding regions, we used the transcript length in our analysis, owing to the fact that there is a very strong correlation between the length of the longest transcript of a gene and its respective gene length (Kendall test: *τ* = 0.72, *p* < 2.20E−16; Supplementary Figure S1). The five biggest genes in terms of transcript length have been studied previously, and they are associated with neuronal functions (Tao and Sampath, 2010; Yamagishi et al., 2011; Hu et al., 2016), cardiac tissue (Ware and Cook, 2017), and cancer (Felder et al., 2014; Table 1). However, the smallest genes might be annotation errors in the genome build.

**Table 1:** List of the top 5 longest protein-coding transcripts in the human genome.

Transcript stable ID	Gene ID	Gene name	Transcript length	Gene length	Exon count	Intron count	Number of SNPs	Protein size
**Longest genes**								
ENST00000589042	ENSG00000155657	*TTN*	109,224	118,976	363	362	69,258	35,991
ENST00000397910	ENSG00000181143	*MUC16*	43,816	43,830	84	83	38,498	14,507
ENST00000262160	ENSG00000175387	*SMAD2*	34,626	36,426	11	10	26,668	467
ENST00000330753	ENSG00000185070	*FLRT2*	33,681	34,901	2	1	25,451	660
ENST00000609686	ENSG00000273079	*GRIN2B*	30,355	30,941	13	12	90,195	1,484

We also built an additional dataset using the principal isoform based on APPRIS annotation (*N* = 19,702; Supplementary Table S2A). We found an overlap of 14,955 transcripts between both datasets, resulting in a 75% overlap with the original transcript length-based dataset. Furthermore, the results presented in the following section were vastly similar to those obtained using the APPRIS dataset (Supplementary Table S2 and Supplementary Figure S2), and therefore we focused our analysis and discussion on the transcript length-based dataset.

### Functional Analysis

One of the main objectives of the present study was to understand whether gene function was associated with gene length. Keeping this in mind, and using a list of the top 5% protein-coding genes with the longest and smallest transcript lengths, we performed a functional analysis using tools like WebGestalt (Liao et al., 2019), DAVID (Huang et al., 2009a,b), KEGG (Kanehisa and Goto, 2000), and Molecular Signature Database (Subramanian et al., 2005; Liberzon et al., 2015).

**Figure 1:**
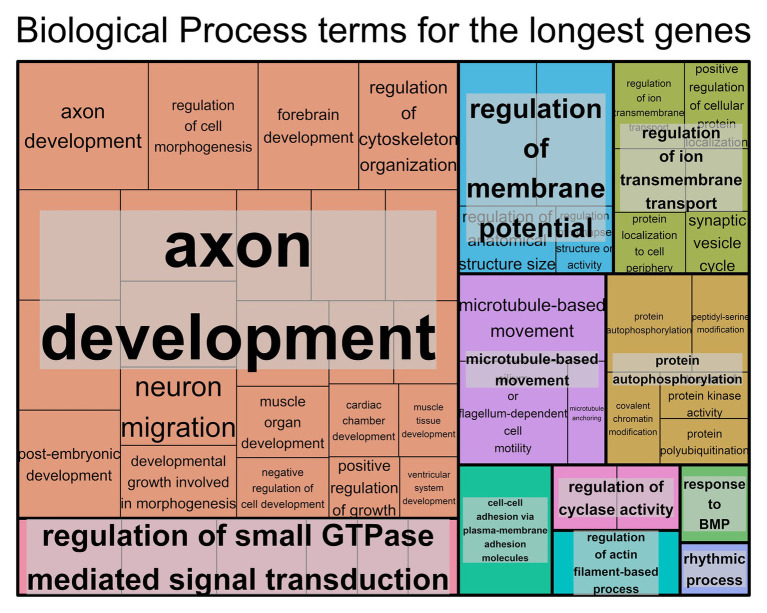
Biological process Gene Ontology (GO) terms found associated with genes with the longest transcript length. Individual compartments represent each biological process term, and compartments were grouped based on semantic similarity. Compartment proportion is based on the false discovery rate (FDR) value. Overrepresentation enrichment analysis was performed with WebGestalt (Liao et al., 2019) and the visualization tool REViGO (Supek et al., 2011) was used to produce this figure. The significance level was *p* < 0.05 and the FDR was set at 0.05. FDR estimation was done using the Benjamini-Hochberg method. Full data available in Supplementary Table S2A.

For the genes with longer transcript lengths (Figure 1), most of the biological functions found seem to be associated with the brain, specifically with regard to neurons. This can also be confirmed when looking at the cellular component (Supplementary Figure S3A) and molecular function (Supplementary Figure S3B) and at the similar results produced using DAVID (Supplementary Table S3). The top 10% longest protein-coding genes produced similar results (Supplementary Table S3 and Supplementary Figures S3E–G).

**Figure 2:**
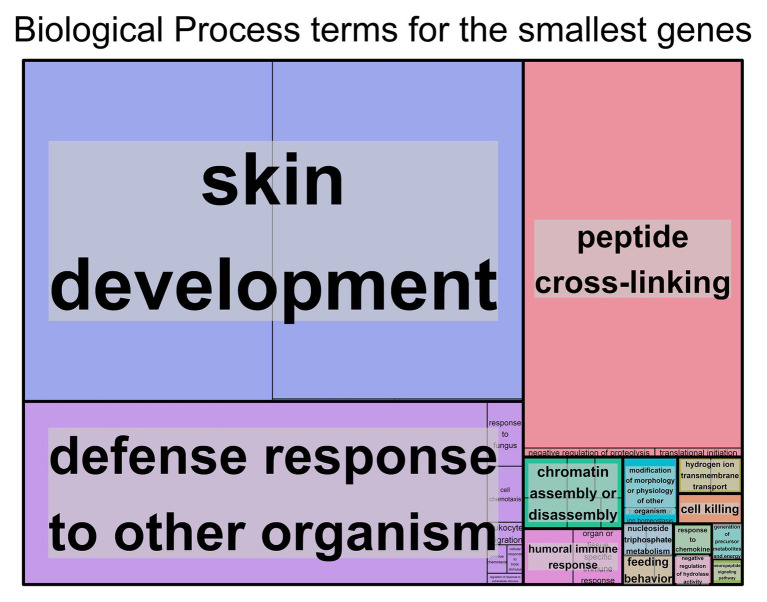
Biological process Gene Ontology (GO) terms found associated with genes with the smallest transcript length. Individual compartments represent each biological process term, and compartments were grouped based on semantic similarity. Compartment proportion is based on the false discovery rate (FDR) value. Overrepresentation enrichment analysis was performed with WebGestalt (Liao et al., 2019) and the visualization tool REViGO (Supek et al., 2011) was used to produce this figure. The significance level was *p* < 0.05 and the FDR was set at 0.05. FDR estimation was done using the Benjamini-Hochberg method. Full data available in Supplementary Table S2B.

For the genes with shorter transcript lengths (Figure 2), most of the biological functions found are related to the skin and the immune system. Cellular component (Supplementary Figure S3C), molecular function (Supplementary Figure S3D), and the DAVID (Supplementary Table S3) results support this observation. Again, the top 10% smallest protein-coding genes produced similar results (Supplementary Table S3 and Supplementary Figures S3 H–J).

**Figure 3:**
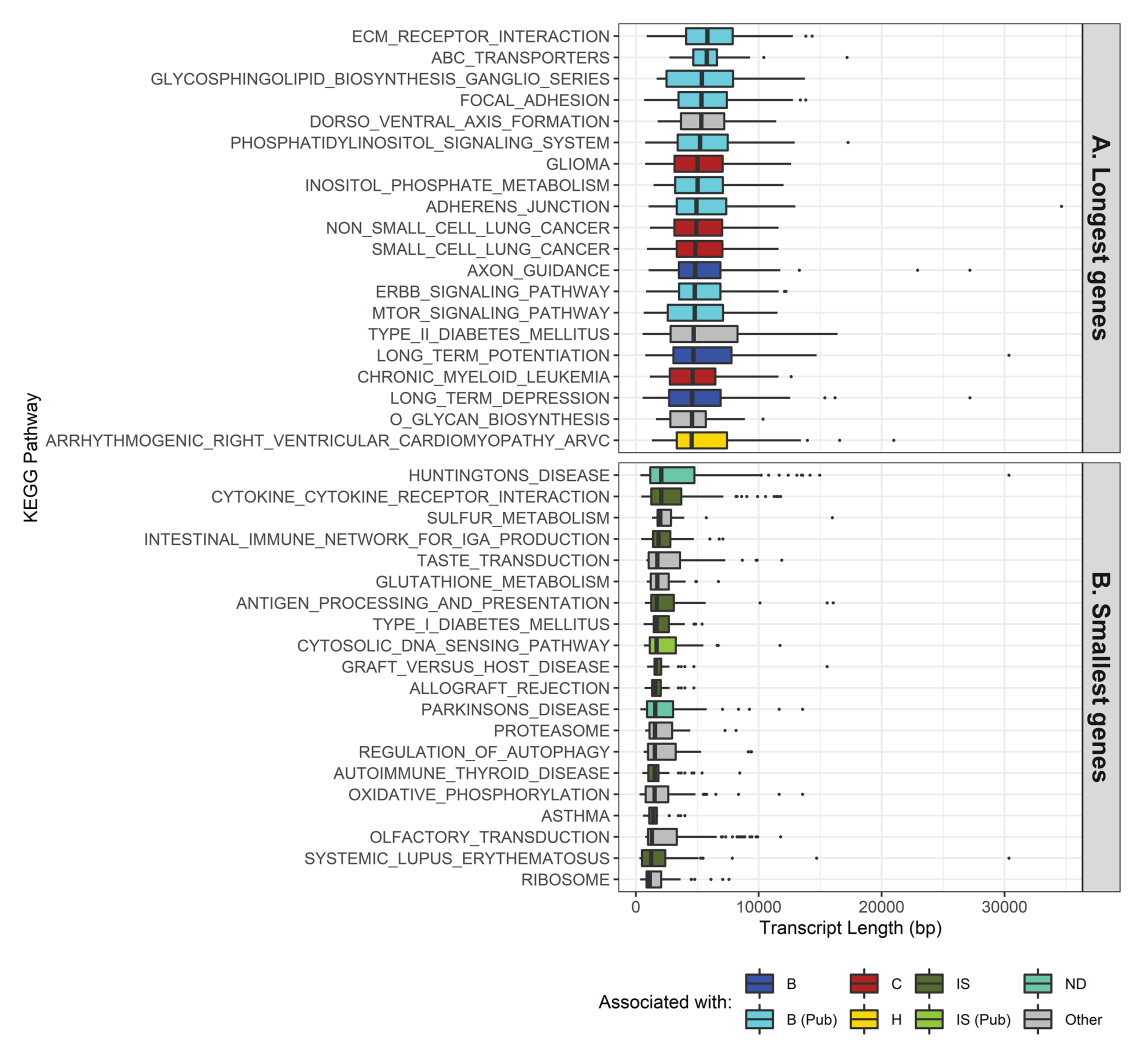
Transcript length distribution per Kyoto Encyclopedia of Genes and Genomes (KEGG) pathway for the longest and smallest genes. *Colors* illustrate what the KEGG pathway has been directly associated with (*B* for brain, *C* for cancer, *H* for heart, *IS* for immune system, and *ND* for neurodegenerative diseases), due to it being stated in the pathway itself or indirectly associated with (Pub tag), by means of literature references. KEGG pathways and genes involved in said pathways were obtained from the Molecular Signature Database (Subramanian et al., 2005; Liberzon et al., 2015). **(A)** Top 20 pathways with the longest genes, ordered by median. **(B)** Top 20 pathways with the smallest genes, ordered by median.

The results for the KEGG pathways (Figure 3A and Supplementary Figure S4) were color coded for each box plot based on their association with the terms we found most relevant (brain, cancer, heart, immune system, muscle, neurodegenerative disease, skin, and others). For cases where there was no direct association, a literature search was done for relevant articles that showed that those pathways were related to the brain (Dermietzel and Spray, 1993; Fisher et al., 2002; Funderburgh, 2002; Lasky and Wu, 2005; Lin et al., 2006; Frere et al., 2012; Kwok et al., 2012; Monje et al., 2012; Russo et al., 2012; Bauer et al., 2014; Kerrisk et al., 2014; Massaly et al., 2014; Mei and Nave, 2014; Stocker and Chenn, 2015; Schnaar, 2016; Zeng et al., 2016; Noelanders and Vleminckx, 2017; Grube et al., 2018; Russo et al., 2018; Dickson, 2019), cancer (Ogretmen, 2018), immune system (Prentki and Madiraju, 2008; Le Floc’h et al., 2011; Barber, 2014; Seif et al., 2017; Zhang et al., 2019), and the skin (Taylor et al., 1991; Fisher and Voorhees, 1996; Iversen and Kragballe, 2000; Ziboh et al., 2000; Slominski et al., 2013). From the total 19,714 genes in our dataset, 5,203 (26%) were annotated with associations to KEGG pathways. The number of genes per pathway can be found in Supplementary Table S4.

Looking at the KEGG pathway results for the longest transcript lengths, we identified pathways associated with the brain, cancer, heart disease, and muscle (Figure 3A and Supplementary Figure S4), while the pathways with the shortest transcript lengths are mostly associated with the immune system; a few of them were also associated with the skin and neurodegenerative diseases (Figure 3B and Supplementary Figure S4).

The full KEGG results (186 gene sets) can be found in Supplementary Figure S4, and the KEGG pathway IDs can be found in Supplementary Table S4.

Finally, we wished to further understand the role of transcript length in the several functions of the immune system. Using the pathway database Reactome (Jassal et al., 2019), we investigated the distribution of transcript length at several ontology levels of the immune system. For the second ontology level, genes of the innate immune system (median = 2,830) and of the cytokine signaling pathways (median = 2,890) are significantly smaller than the genes from the adaptive immune system (median = 3,112), although the difference between them was not substantial (Supplementary Figure S5A). Regarding the third (Supplementary Figure S5B) and fourth (Supplementary Figure S5C) ontology levels, shorter genes appear to participate in the antimicrobial peptide pathway (defensins). Interestingly, part of the complement pathway (activation of C3 and C5; Supplementary Figure S5C) includes longer genes, but overall, the complement pathway (Supplementary Figure S5B) appears to be on the shorter-transcript-length side.

### Gene Properties Correlated With Transcript Length

**Figure 4:**
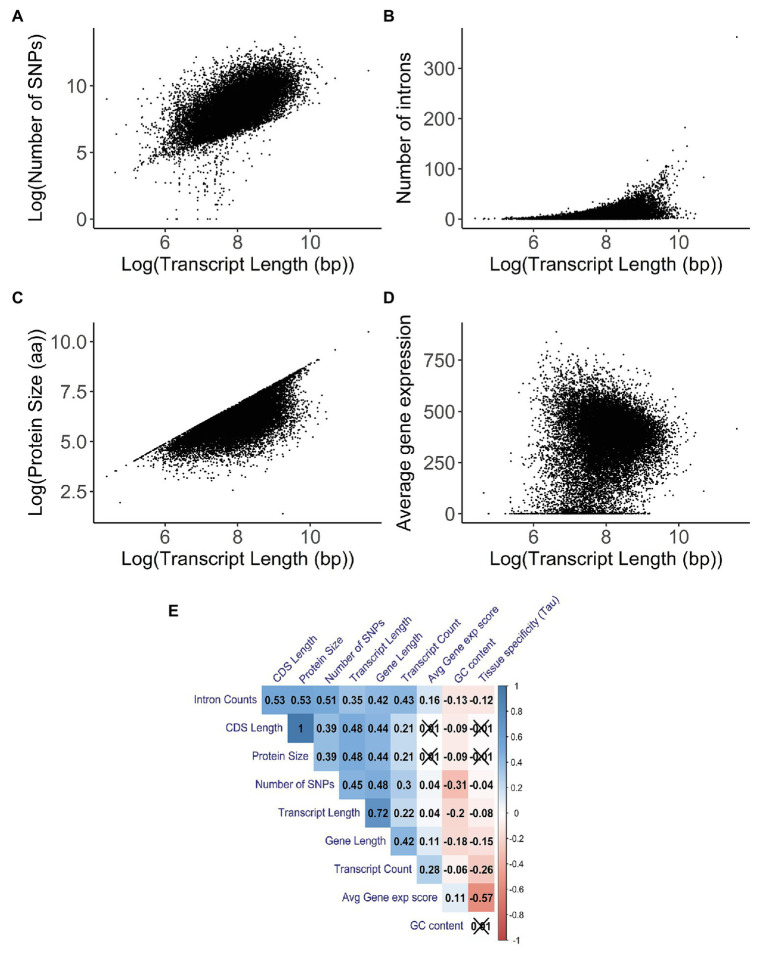
Correlation analysis between transcript length (in base pairs) and other gene characteristics. A 2D density plot can be found in Supplementary Figure S4B. Parts **(A–D)** have been logarithmically transformed in order to help visualize their relationship and/or account for the skewing introduced by outliers. The original versions of the figures can be found in Supplementary Figures S4C–F. **(A)** Correlation between the log-transformed number of single nucleotide polymorphisms (SNPs) and the log-transformed transcript length (in base pairs; Kendall test: *τ* = 0.45, *p* < 2.20E−16). The number of SNPs and the transcript length for each transcript were obtained using biomart. **(B)** Correlation between the number of introns and the log-transformed transcript length (in base pairs; Kendall test: *τ* = 0.35, *p* < 2.20E−16). The number of introns and the transcript length for each transcript were obtained using biomart. **(C)** Correlation between the log-transformed protein size (in amino acids) and the log-transformed transcript length (in base pairs; Kendall test: *τ* = 0.48, *p* < 2.20E−16). Protein size and transcript length were obtained using biomart. **(D)** Correlation between the average gene expression and the log-transformed transcript length (in base pairs; Kendall test: *τ* = 0.04, *p* = 3.06E−14). Average gene expression was obtained from the UCSC Genome browser; this value was derived from the total median expression level across all tissues and was based on the GTEx project. Transcript length was obtained using biomart. **(E)** Correlation matrix between gene properties. Kendall’s test was used as a measurement of correlation, with the *numbers* and the *gradient of colors* symbolizing the tau values for each comparison. The values for the number of SNPs are for each transcript. Values that are *crossed out* are not statistically significant. Values are clustered together based on their tau values.

In order to understand the relationship between transcript length and other gene characteristics, a correlation analysis was performed. When looking at the number of SNPs for each transcript (Figure 4A), there was a significant positive correlation with transcript length (Kendall test: *τ* = 0.45, *p* < 2.20E−16). Similar results were found when comparing the number of SNPs per gene with gene length (Kendall test: *τ* = 0.49, *p* < 2.20E−16; Supplementary Figure S6A). After comparing the number of introns and the transcript length (Figure 4B), we found a weak but significant positive correlation between these two variables (Kendall test: *τ* = 0.35, *p* < 2.20E−16). The strongest positive correlation (Kendall test: *τ* = 0.48, *p* < 2.20E−16) was the association with protein size (Figure 4C); the weakest correlation (Kendall test: *τ* = 0.04, *p* = 3.06E−14) was the association with average gene expression (Figure 4D).

Additionally, for the correlations with the transcript count (Supplementary Figure S6G) and GC content (Supplementary Figure S6H), we observed a weak but significant positive correlation (Kendall test: *τ* = 0.22, *p* < 2.20E−16) and a weak significant negative correlation (Kendall test: *τ* = −0.19, *p* < 2.20E−16), respectively.

We were also interested in understanding the effect of transcript length in specific mutations. We observed some strong and statistically significant correlations between transcript length and synonymous (Kendall test: *τ* = 0.44, *p* < 2.20E−16; Supplementary Figure S6I) and missense (Kendall test: *τ* = 0.42, *p* < 2.20E−16; Supplementary Figure S6J) mutations. However, in the case of nonsense mutations (Kendall test: *τ* = 0.21, *p* < 2.20E−16; Supplementary Figure S6K), a weaker but significant positive correlation with transcript length was observed. This was followed by the calculation of missense/synonymous (MIS/SYN) and nonsense/synonymous (NONS/SYN) rates in order to measure the functional importance of gene length. We observed that these ratios had similarly negative correlations with transcript length, with MIS/SYN having a weaker significant correlation (Kendall test: *τ* = −0.07, *p* < 2.20E−16; Supplementary Figure S6L) than NONS/SYN (Kendall test: *τ* = −0.19, *p* < 2.20E−16; Supplementary Figure S6M).

In order to better understand whether the correlations found were solely due to the transcript length or whether other factors were influencing them, we built a correlation matrix with several gene characteristics (Figure 4E). We observed that properties like intron counts, CDS length, protein size, number of SNPs, and transcript counts have strong positive correlations among themselves, some of which were stronger than any other correlations with transcript length. This indicates that the strong correlations with transcript length might not be due to the sole action of the transcript length itself, but rather due to the combined effects between several gene characteristics that also correlate with each other. Furthermore, partial correlations were performed for the number of SNPs (Kendall test: *τ* = 0.27, *p* < 2.20E−16), intron count (Kendall test: *τ* = −0.02, *p* = 2.33E−05), protein size (Kendall test: *τ* = 0.34, *p* < 2.20E−16), average gene expression (Kendall test: *τ* = 0.02, *p* = 5.68E−07), transcript count (Kendall test: *τ* = 0.05, *p* < 2.20E−16), and GC content (Kendall test: *τ* = −0.09, *p* < 2.20E−16) against transcript length while accounting for these other variables. The differences in the tau values between the correlations and partial correlations further illustrate that these variables are not independent of each other.

### Distribution of Transcript Length and Expression in Human Tissues

In this present work, we have found that the transcript length seems to peak at 2,065 bp, with smaller transcripts being more common than longer ones (Supplementary Figure S7A). As described previously (Gorlova et al., 2014), the distribution of the number of introns in the human genome (Supplementary Figure S7B) has a mode of three introns, and there are very few genes with a large number of introns. The gene with the most introns is TTN, with 362 introns, which is also the gene with the longest transcript length.

**Figure 5:**
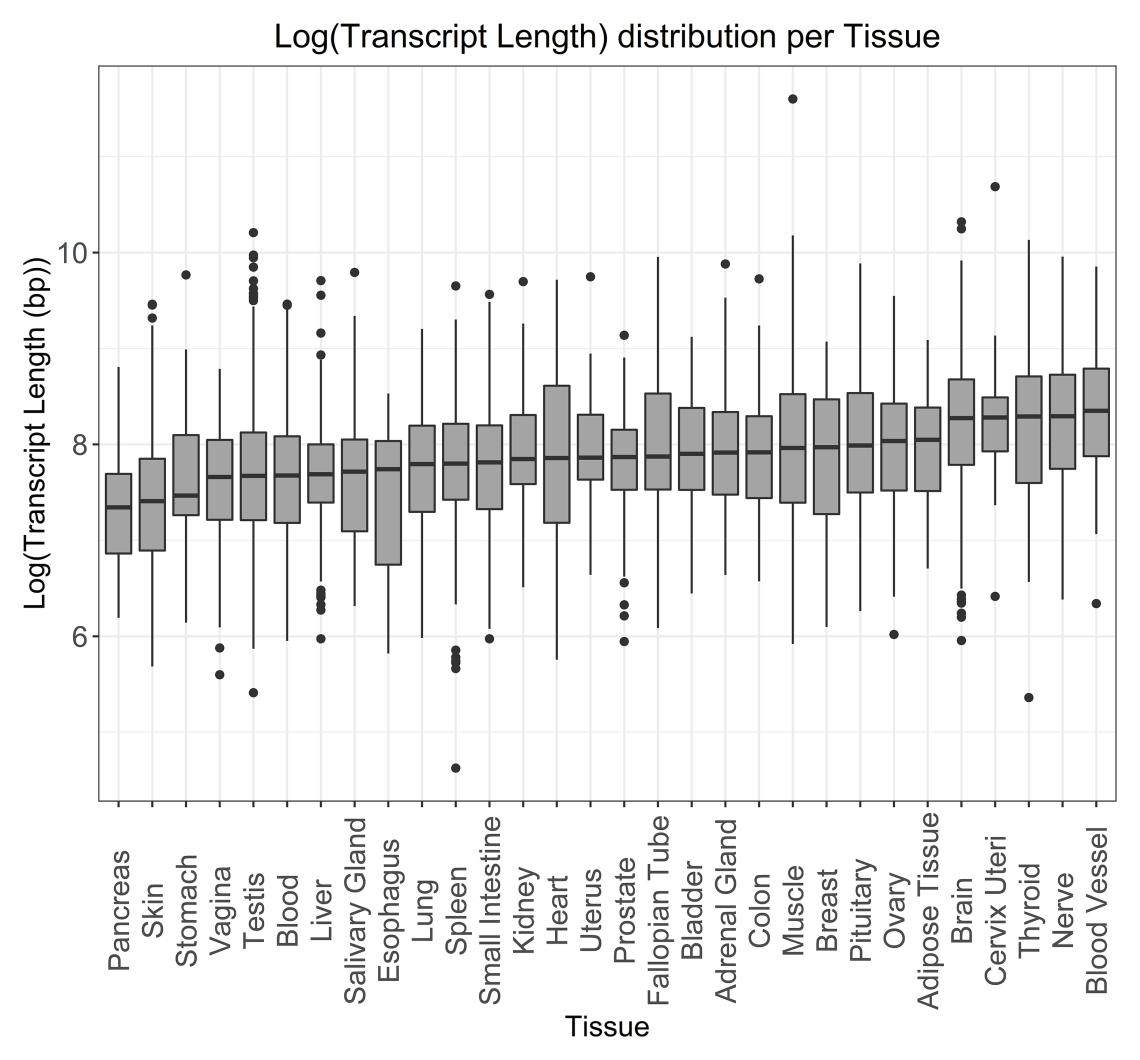
Log-transformed transcript length distribution for genes specifically expressed in the given tissues. Tissue specificity was defined as a gene having a tau specificity score greater than 0.8.

To better understand the distribution of transcript length in human tissue expression, we used tau values obtained from GTEx data (Palmer et al., 2019). Tau was used as a measure of tissue specificity, based on the expression profile in different tissues, with values ranging from 0, for broadly expressed genes, to 1, for tissue-specific genes (Yanai et al., 2005). For genes with a tau value above 0.8 (Figure 5 and Supplementary Figure S8 for the non-log-transformed version), we observed that longer tissue-specific genes are often associated with the blood vessel, nerve, thyroid, cervix uteri, and brain, while smaller tissue-specific genes are found in the pancreas, skin, stomach, vagina, and testis.

### Aging and Transcript Length

Aging is an important factor in our lives, and it affects most organisms. We were curious to see whether, for genes related to aging, the distribution of transcript length was significantly different from the rest of the protein-coding genes. We observed (Supplementary Figures S9A,B) that the genes associated with aging (*N* = 307; Tacutu et al., 2018) have longer transcript lengths (median = 3,517) when compared with the rest of our dataset (median = 2,956) and that this difference of medians was significant (Wilcoxon rank-sum test: *p* = 0.00036).

To further understand whether longer or smaller genes were more prominent with age, we used genes from aging gene expression signatures obtained from a meta-analysis in human, mouse, and rat tissues (Palmer et al., 2019). Genes from this signature were either overexpressed (*N*_Total_ = 449, *N*_Brain_ = 147, *N*_Heart_ = 35, *N*_Muscle_ = 49) or underexpressed (*N*_Total_ = 162, *N*_Brain_ = 16, *N*_Heart_ = 5, *N*_Muscle_ = 73) with age. Overall, the difference in the medians for the distribution of transcript length in the genes overexpressed (median = 3,068) and underexpressed (median = 3,026.5) with age was not observed to be significant (Wilcoxon rank-sum test: *p* = 0.81; Supplementary Figure S9C). However, the tissue-specific signatures of aging show that the brain favors smaller genes with age (Wilcoxon rank-sum test: *p* = 0.00086, median for overexpression in the brain = 2,651, median for underexpression in the brain = 5,824; Supplementary Figure S9D).

### Evolution and Transcript Length

The relationship between intronic burden and evolution has been established before (Gorlova et al., 2014), but very few studies approached this on a gene length front. Therefore, we obtained the dN and dS values for three organisms paired with human – mouse (Supplementary Figure S10A), gorilla (Supplementary Figure S10B), and chimpanzee (Supplementary Figure S10C) – and we aimed to see how the distribution of transcript length happened in function of their dN/dS ratios. Overall, longer genes were associated with a dN/dS ratio less than 1 (the median transcript lengths are 3,294, 3,377, and 3,338 for mouse, chimpanzee, and gorilla, respectively), while smaller genes seem to be more associated with dN/dS ratios above or equal to 1 (the median transcript lengths are 1,171.5, 2,229.5, and 2,092 for mouse, chimpanzee, and gorilla, respectively), and the medians for both transcript length groups were always significantly different (Wilcoxon rank-sum test: *p* = 0.00073 for mouse and *p* < 2.2E−16 for both gorilla and chimpanzee).

### Co-Expression Analysis and Protein-Protein Interactions

**Figure 6:**
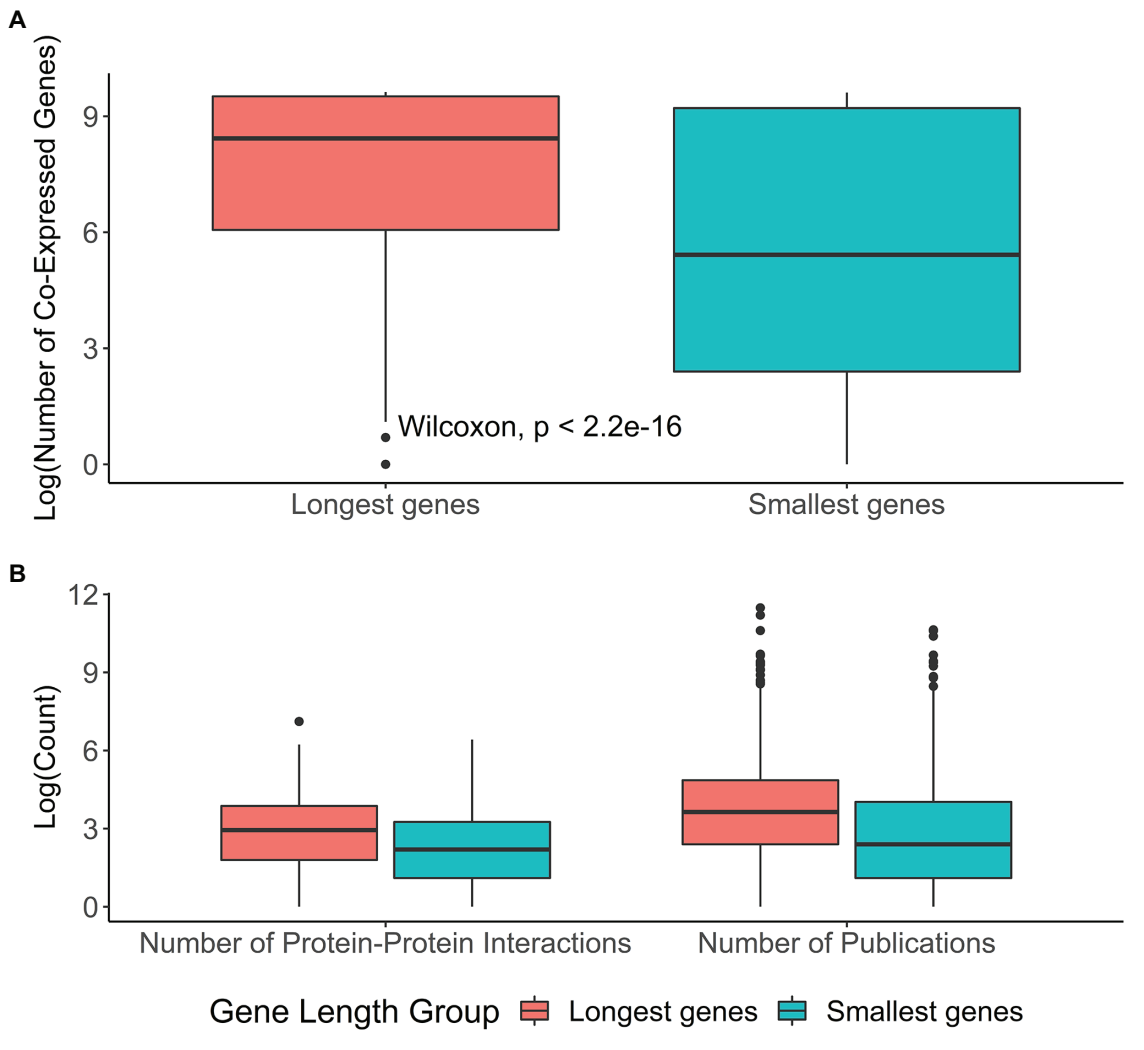
Co-expression and protein-protein interaction results pertaining to the longest and the smallest genes. The High group corresponds to the top 5% longest genes found in our original dataset (*N*_High_ = 986), while the Low group corresponds to the top 5% smallest genes found in our original dataset (*N*_Low_ = 986). **(A)** Distribution of the log-ransformed number of co-expressed genes for the long genes and the small genes. The number of co-expressed genes were obtained from data publicly available in GeneFriends (van Dam et al., 2015). **(B)** Distribution of the number of protein-protein interactions and the number of publications for longer and smaller genes, all log-transformed. The number of protein-protein interactions were obtained from BioGRID (Stark, 2006) and the number of publications obtained from PubMed.

Co-expression networks can help us better understand the functions of genes that are often expressed together and thus tend to be functionally related (van Dam et al., 2018). In order to determine whether gene length influenced the amount of co-expressed partners, we used data from GeneFriends (van Dam et al., 2015; Supplementary Table S5). We observed a weak correlation between transcript length and the number of co-expression partners in our dataset (Kendall test: *τ* = 0.10, *p* < 2.2E−16; Supplementary Figure S11A). However, despite this weak correlation, longer genes appear to have more co-expressed gene partners than do smaller genes (Wilcoxon rank-sum test: *p* < 2.2E−16; Figure 6A; not-transformed figure in Supplementary Figure S11B, median values of the co-expression partners for longer genes = 2,725, median values of the co-expression partners for smaller genes = 32). We further analyzed the top and lower hundred human co-expressed genes from the GeneFriends database (Supplementary Table S5) and observed that the top highly co-expressed genes in the database have significantly longer transcript lengths (Wilcoxon rank-sum test: *p* = 0.00072, median = 3,880; Supplementary Figure S11C) with respect to the bottom ones (median = 2,587.5).

To determine whether transcript length also influenced the number of protein-protein interactions, we used the protein-protein interaction data from BioGRID (Stark, 2006; Supplementary Table S6). The results obtained were similar to the co-expression, where a weak correlation was observed between transcript length and the number of protein-protein interactions (Kendall test: *τ* = 0.06, *p* < 2.2E−16; Supplementary Figure S12A).

To ascertain that the interactions found were not due to publication bias, we obtained the number of publications for each gene from PubMed and compared these to gene length and to the number of interactions (Figure 6B). We observed that the number of interactions and publications were significantly different between each gene length group (Wilcoxon rank-sum test: *p* < 2.2E−16 for both comparisons), with both being higher for the group comprising longer length genes. In order to assess the level of influence of publication bias in our protein-protein interaction dataset, we used correlations between the values of protein-protein interactions and the number of publications, and we observed that, for both gene length groups, the correlations were not the strongest (Kendall test: longest genes, *τ* = 0.26, *p* < 2.2E−16; smallest genes, *τ* = 0.36, *p* < 2.2E−16), implying that while there might be some publication bias in effect, the strength of that effect is rather weak. To further support this, we carried out a multivariable regression to determine the influence of the number of publications and transcript length in the number of protein-protein interactions (PPI) found (*p* of *F* statistic = 1.936E−10). We again observed that the number of publications did not have a significant association with the number of PPI (*t* = 1.41, *p* = 1.59E−1), unlike transcript length (*t* = 6.55, *p* = 6.06E−11). This resulted in the model being extremely inaccurate (*R*^2^ = 0.0022, residual standard error = 78.05).

In the group of the longest genes, 208 (21%) entries had zero protein-protein interactions, while for the smallest group of genes, 544 (55%) entries had zero protein-protein interactions. This means that there were either no physical interactions for those genes or that there were no entries in BioGRID for them. So as to account for this, and similarly to what we did for the co-expression analysis, we extracted the top 100 genes with the most and fewest protein-protein interactors (without null values) in our dataset and observed the distribution of their transcript length. We observed that the genes with the most protein-protein interactions were longer (median transcript length = 3,737) than the genes with the smallest amount of protein-protein interactions (Wilcoxon rank-sum test: *p* = 0.039, median transcript length = 2,764; Supplementary Figure S12B).

## Discussion

With this work, we tried to elucidate the factors associated with gene length and, in particular, whether gene length is associated with the function of the resulting proteins in the cell. Even looking at the five longest genes, we can get a small glimpse into the possible functions associated with gene length. *TTN* is the longest transcript in the human genome and serves several important functions in the skeletal and cardiac muscles and is often involved in structure, sensory, and signaling responses (Chauveau et al., 2014; Savarese et al., 2016; Ware and Cook, 2017). The mucin *MUC16* (or CA125) is mostly known as a biomarker in ovarian cancer and is used to monitor patients as an indicator of cancer recurrence (Felder et al., 2014; Haridas et al., 2014; Das and Batra, 2015). Furthermore, MUC16 normally functions as a protector of epithelial cells (Haridas et al., 2014). SMAD family member 2 (*SMAD2*) is thought to play a critical role in neuronal function (Tao and Sampath, 2010) and to have a protective role in hepatic fibrosis (Xu et al., 2016). The gene *FLRT2* is believed to have a role in tumor suppression in breast and prostate cancers (Wu et al., 2016; Bae et al., 2017), and in mouse models, *FLRT2* has been found as a guiding agent in neuronal and vascular cells (Yamagishi et al., 2011; Seiradake et al., 2014). For the *GRIN2B* gene, it has been shown to play an important role in neuronal development and in cell differentiation in the brain (Hu et al., 2016; Bell et al., 2018). We cannot obtain any information at the moment pertaining to the function of the five smallest genes since all of them are either novel and have yet to be properly studied and, indeed, could be annotation errors in the human genome assembly.

In order to understand the effects of gene length on protein function, we performed a functional analysis. For longer genes, the GO terms obtained were mostly associated with neurons; for example, terms like axon development, axon part, neuron-to-neuron synapse, actin and cell polarity (Polleux and Snider, 2010), and GTPases (Polleux and Snider, 2010). For tissue-specific genes, the brain and nerves also had the longest genes. Looking at the KEGG pathways associated with the longest genes, the categories present are in the brain, cancer, heart diseases, and muscle. Previous studies have associated longer genes with neurons (Zylka et al., 2015; Takeuchi et al., 2018; McCoy and Fire, 2020) and muscle (Hosokawa et al., 2019). Due to the very nature of longer genes, one expects high rates of mutation, not only due to their size but also due to possible collisions between the RNA polymerase and the DNA polymerase, which cause instability and possible mutations (Helmrich et al., 2011). It is not surprising to find associations between longer genes and cancer (Sahakyan and Balasubramanian, 2016) and heart pathologies often caused by mutations in particularly long genes, like *DSC2* and *TTN* (Jefferies and Towbin, 2010; Maron and Maron, 2013; Corrado et al., 2017).

Looking at smaller genes, most of the GO terms were associated with the skin, for example skin development and cornified envelope, or with the immune system, for example, defense response to other organisms and receptor agonist activity. Smaller tissue-specific genes also have a major presence in the skin. With regard to the KEGG pathways associated with the smaller genes, most pathways were involved in the immune system, with a few also being present in neurodegenerative diseases and in the skin. In addition, the Reactome immune system pathway with the smallest genes was found to be associated with antimicrobial peptides (defensins). Defensins are small peptides that play a role in innate immunity and have been found to be expressed in several mammal species (Holly et al., 2017). Moreover, the complement cascade, another important function in the innate immune system, was also associated with smaller genes for the most part, with a specific pathway (activation of C3 and C5) being the exception. Previous studies have observed that most genes associated with immune functions are rather small in size (Pipkin and Monticelli, 2008). However, to our knowledge, there are no previous studies to support the association of smaller genes with skin development.

In spite of this, our findings led us to believe that there is a disparity in gene sizes for genes that have a role or are present in tissues with very little to almost no development postnatally (like neurons) and for genes (not involved in housekeeping) that are quite frequently expressed during a human’s whole lifetime (like in skin development and immune response) or are involved in functions that require fast responses. Corroborating our findings for the functional analysis, a recent preprint has showed that, with age, there is a downregulation of long transcripts and an upregulation of short transcripts, in a phenomena they named “length-driven transcriptome imbalance,” which, in humans, affects the brain the most (Stoeger et al., 2019). As we observed, smaller genes can be associated with the immune system – and inflammation has a role in many aging-related diseases (Goldberg and Dixit, 2015) – while longer genes are mostly associated with brain development, a function that happens early in life.

In terms of gene characteristics, there was no strong correlation with transcript length. The strongest positive correlations were with protein size and number of SNPs, with transcript count, number of introns, GC content, and average gene expression having a weak significant positive correlation.

The correlation we observed between average gene expression and transcript length was not in line with previous observations, which suggested that highly expressed genes are often shorter in length (Urrutia and Hurst, 2003). We observed that, among smaller genes, the average gene expression was, in fact, the highest (Supplementary Figure S6F). However, shorter genes also had a great variability in the average gene expression values, and there was almost no correlation between transcript length and average gene expression. What has been stated in the previous studies is relevant, but the whole image is not captured properly. Rather than stating that the smaller genes are highly expressed, it is more accurate to say that smaller genes have a greater variability of levels of expression than longer genes. Previous studies have shown that the length of messenger RNA (mRNA) will affect the translation dynamics, with smaller mRNAs producing more proteins (in both normal and cancer cells) than their longer counterparts, a phenomenon that may be due to energy conservation in the case of translation errors or misfolding (Valleriani et al., 2011; Wang et al., 2013; Guo et al., 2015). This matches with what we observed as well: smaller genes will have a more important role in day-to-day functions due to their ability to be rapidly translated and in more numbers; on the other hand, longer genes are important for early life development, especially in the brain and heart, where it would be worth spending more energy in more long-lasting, robust functions.

While the observation that the number of SNPs is correlated with transcript length is not surprising since, logically, longer genes will have a higher probability of accumulating more mutations than smaller genes, it is unexpected that the correlation was not stronger. To further explore this, we used different mutation ratios and observed their relationship with transcript length. Similar to the correlation results for the number of SNPs, both synonymous and missense mutations were also highly correlated with transcript length. It is particularly interesting that the correlation values were so high for missense mutations since these may cause loss of function in the resulting protein. Likewise, it could be one of the reasons why the correlation between nonsense mutations and transcript length is weaker than that for synonymous and missense mutations. Other works (Gorlova et al., 2014) have used the MIS/SYN and NONS/SYN ratios as a measure of functional importance, where, if a gene is important in terms of its function, it will be less tolerant toward the accumulation of missense and nonsense mutations. We observed that there was a, albeit faint, negative correlation between the ratios MIS/SYN and NONS/SYN and transcript length, which, based on the notions in the work of Gorlova et al., would imply that longer genes appear to be more functionally important than smaller genes. The negative correlation between the ratios MIS/SYN and NONS/SYN showed that longer genes may have more mechanisms in place to prevent loss-of-function mutations when compared with synonymous mutations. Moreover, we have to take account of “outliers” when looking into the correlation between transcript length and protein size (Supplementary Figure S6E), specifically for longer genes. One would expect that, for longer genes, the proteins produced would have a size comparable to their length and not be extremely small. However, some long transcripts result in small proteins due to the presence of very long 3’ UTR regions. While these regions still account for the calculation of transcript and gene size, they are not translated into the protein, causing the presence of these “outliers.” Previous studies have shown that the brain has a preference for these long 3’ UTR regions (Miura et al., 2013; Wang and Yi, 2014).

Interestingly, we noticed that genes associated with aging tend to be longer than the rest of the protein-coding genome. Moreover, we also showed that the overall (not tissue-dependent) expression of genes with age appears to be unrelated to transcript length and that the brain seems to favor the expression of smaller genes with age. The latter result is in line with the observations by Stoeger et al. (2019) who also witnessed an upregulation of smaller transcripts with age, especially in the brain. These results also make sense because small genes are often associated with immune function, which is often upregulated with age (de Magalhães et al., 2009). Furthermore, Palmer et al. (2019) observed that the genes overexpressed with age in the brain were mostly associated with immune functions. However, our results pertaining to the overall expression of genes with age are different from what Stoeger et al. (2019) observed that transcript length is an important source of aging-dependent changes in expression. It is possible that these differences between our results and those of Stoeger et al. (2019) are due to differences in the underlying datasets.

When comparing gene length with the dN/dS ratio for three organisms (gorilla, chimpanzee, and mouse), longer genes appear to evolve under stronger evolutionary constraints. Previous studies have shown that, for genes classified as “old” (by virtue of having orthologs in older organisms), their length will be longer, they will have more introns, and they evolve more slowly than smaller genes (Wolf et al., 2009; Vishnoi et al., 2010).

In terms of the co-expression analysis and protein-protein interactions, the longer genes, in general, had the most co-expression partners and protein-protein interactions. Further validating our observations, we also saw that the top hundred highest co-expression genes and PPI were longer in length as compared to the lowest co-expression genes and PPI. In light of these results, it is important to point out that gene length is a potential bias in large-scale genomic and systems biology studies that scientists should be aware of.

Not all genes are studied at the same depth. Some genes have more information related to expression or function than others. We observed this especially within our list of the 5% longest and smallest genes. Longer length genes had more functional information than shorter ones. We also observed that longer genes have more associated publications than smaller genes. Indeed, other groups have found that gene length can be an important predictor of the number of publications and that novel genes are not often studied to their full capacity (Stoeger et al., 2018), while others have found that genetic associations tend to be more biased toward longer genes (Mirina et al., 2012; de Magalhães and Wang, 2019).

The present study has its limitations. One of the limitations for this sort of study is that the results might be “time-specific.” With new discoveries related to the human genome and its genes, the trends here observed might change, specifically when it concerns the currently untapped field of smaller genes. Similarly, as we previously noted, longer genes have a lot more information related to them when compared with their smaller counterparts. While our findings with respect to the longer genes might be more reliable, we cannot have the same confidence in the case of the smaller genes, considering that a lot of these genes have yet to be properly studied. Moreover, while smaller genes might be annotation errors, they could also be pseudo-genes or even non-coding genes that possess an open reading frame (ORF) that were missed when Ensembl scanned for pseudo-genes. Even after taking into account the above limitations, however, the present study still provides novel insights pertaining to gene length and its possible role in early life development, diseases, and response time in the human genome.

## Conclusion

In this work, we aimed to further understand the relationships between gene length (mostly using the length of a gene’s longest transcript as a proxy for gene length) and gene function as well as factors associated with gene length. We observed that, for most of the factors studied, there was not a particularly strong correlation with transcript length. The strongest correlations were observed with the number of SNPs and protein size. We also showed that, for smaller genes, their association with high levels of expression is not entirely correct and that, instead, there is great variability of expression values among them. We also observed that longer genes appear to have more co-expression partners and protein-protein interactions in comparison to their smaller counterparts.

At the functional level, we observed that longer genes tend to be associated with functions in the brain, cancer, heart, and muscle, while smaller genes are associated with the immune system, skin, and neurodegenerative diseases. This led us to believe that gene length could be associated with the frequency of usage of the gene, with longer genes being less often used past development and smaller genes playing a frequent role daily in the human body, like the immune system.

In conclusion, gene size does matter: longer genes tend to have more SNPs, are more likely to be important in development, have more interactions, and, ultimately, are more studied.

## Data Availability Statement

The original contributions presented in the study are included in the article/Supplementary Material and in a GitHub repository (https://github.com/maglab/GeneLength_supplementary), further inquiries can be directed to the corresponding author.

## Author Contributions

JM, GA, and IL conceived the study. IL and PR performed the analysis. IL prepared the figures. JM, PR, GA, and IL drafted and finalized the paper. All authors contributed to the article and approved the submitted version.

### Conflict of Interest

The authors declare that the research was conducted in the absence of any commercial or financial relationships that could be construed as a potential conflict of interest.
