# Supplementary material for: Gene Size Matters: An Analysis of Gene Length in the Human Genome
Source: Front Genet. 2021 Feb 11;12:559998. doi: 10.3389/fgene.2021.559998 (PMC7905317; doi:10.3389/fgene.2021.559998)
Supplement: Supplementary file 7 [file Data_Sheet_1.pdf]

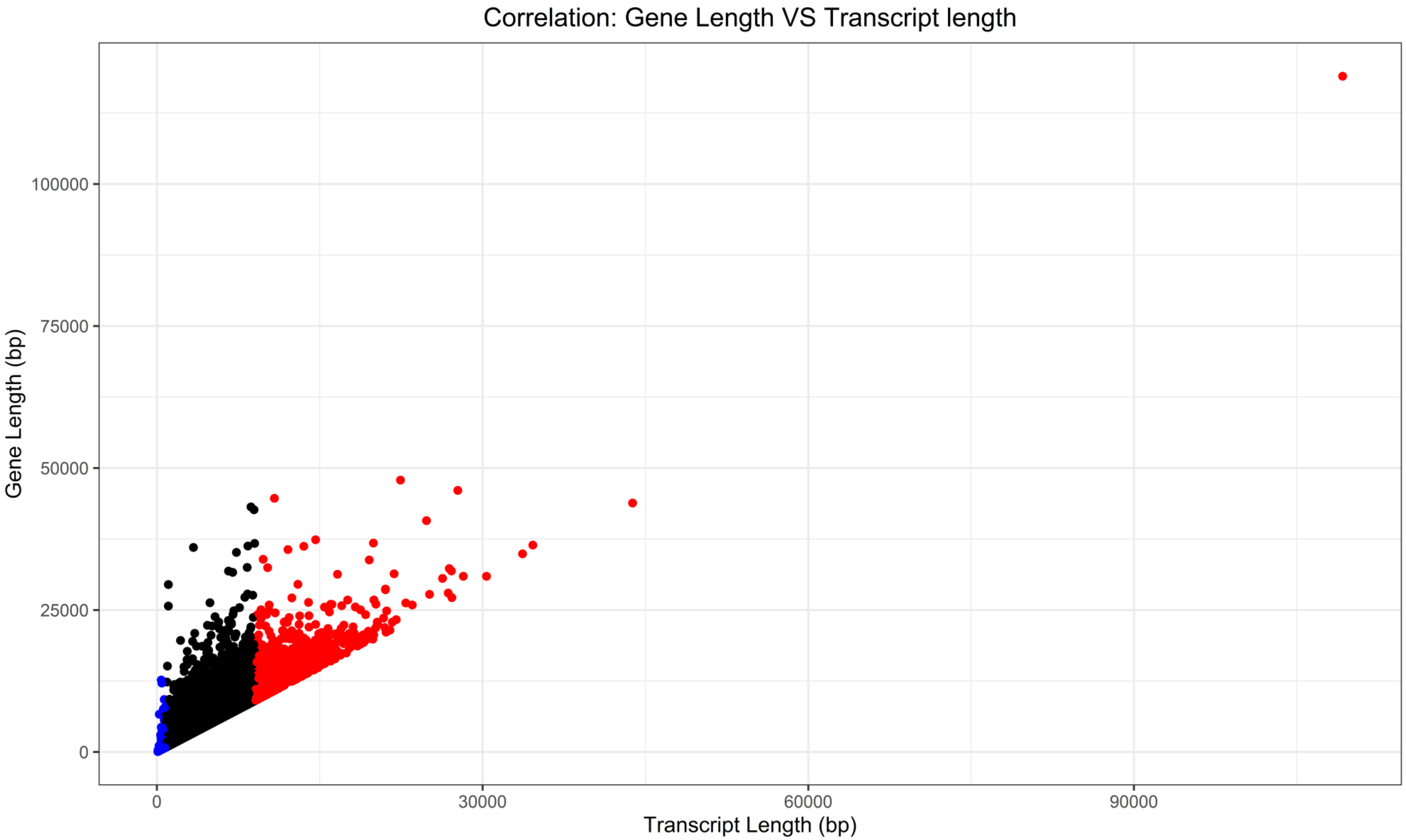

Supplementary Figure 1A.
Correlation between Gene Length and Transcript Length from the longest transcript in a gene. Blue indicates the top 5% smallest transcripts. Red indicates the top 5% longest transcripts. Gene Length and Transcript Length was obtained using biomart.

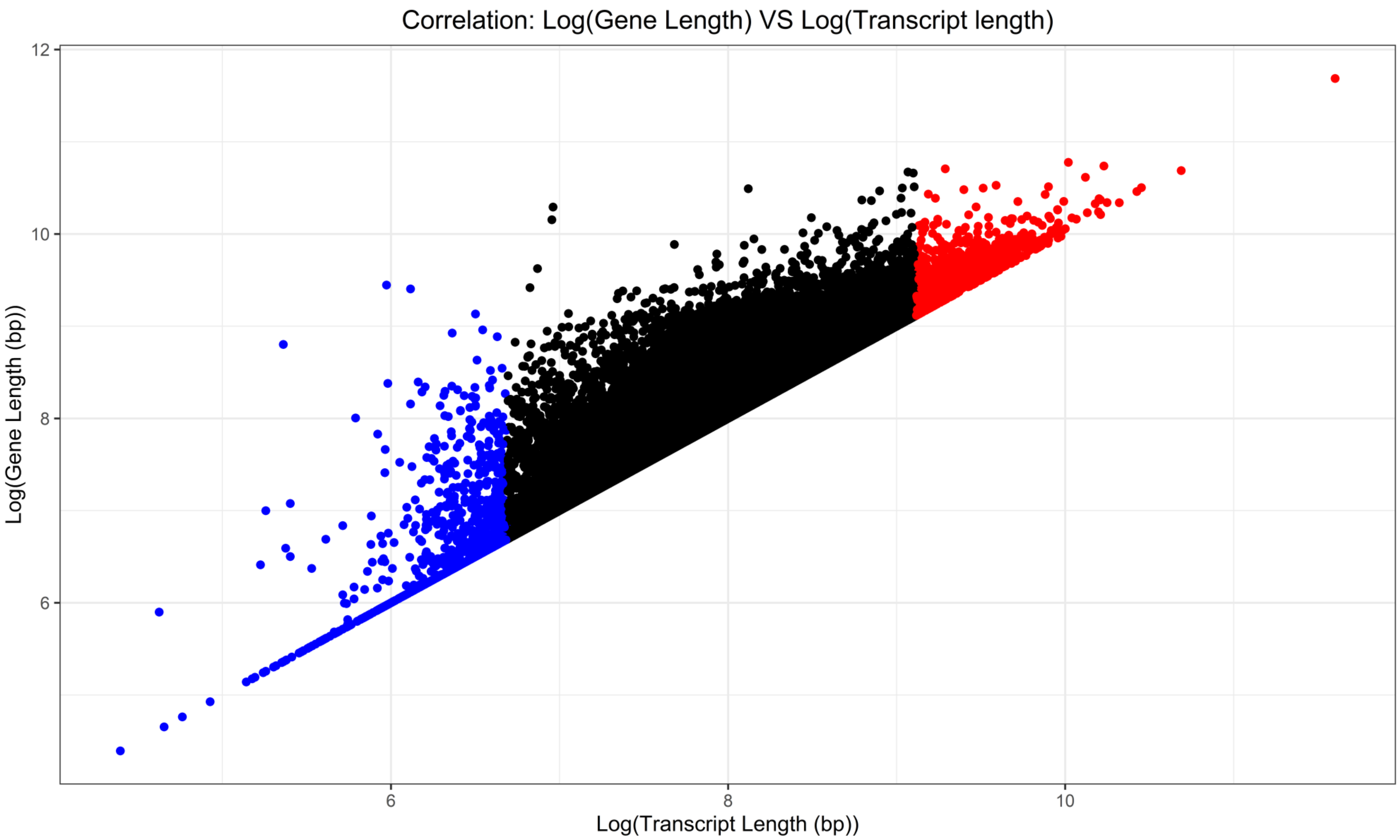

Supplementary Figure 1B.
Correlation between the log transformed Gene Length and the log transformed Transcript Length from the longest transcript in a gene. Blue indicates the top 5% smallest transcripts. Red indicates the top 5% longest transcripts. Gene Length and Transcript Length was obtained using biomart.

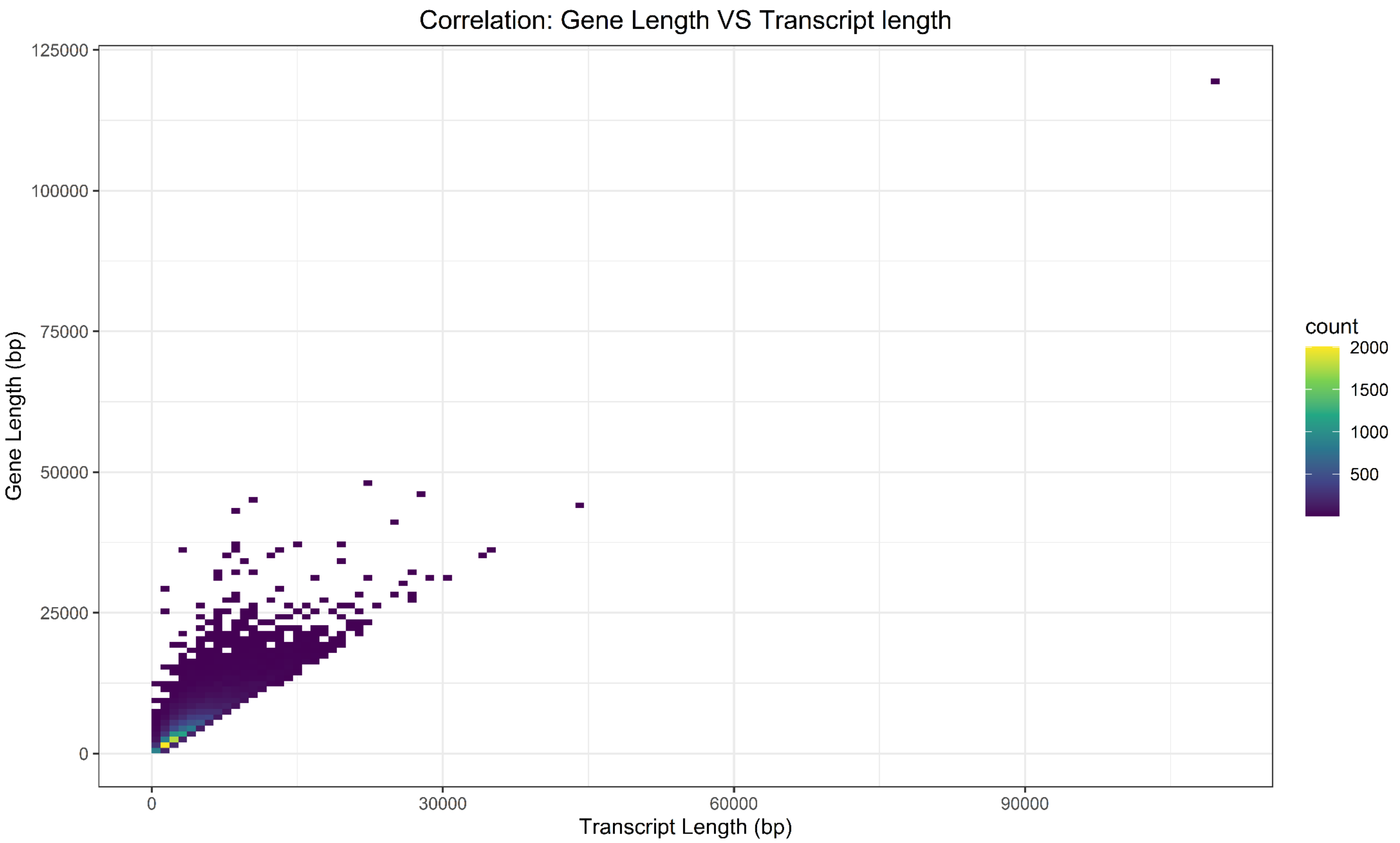

Supplementary Figure 1C.
2D density plot of the correlation between Gene Length and Transcript Length from the longest transcript in a gene. Gene Length and Transcript Length was obtained using biomart.
