# Supplementary material for: Gene Size Matters: An Analysis of Gene Length in the Human Genome
Source: Front Genet. 2021 Feb 11;12:559998. doi: 10.3389/fgene.2021.559998 (PMC7905317; doi:10.3389/fgene.2021.559998)
Supplement: Supplementary file 8 [file Data_Sheet_2.pdf]

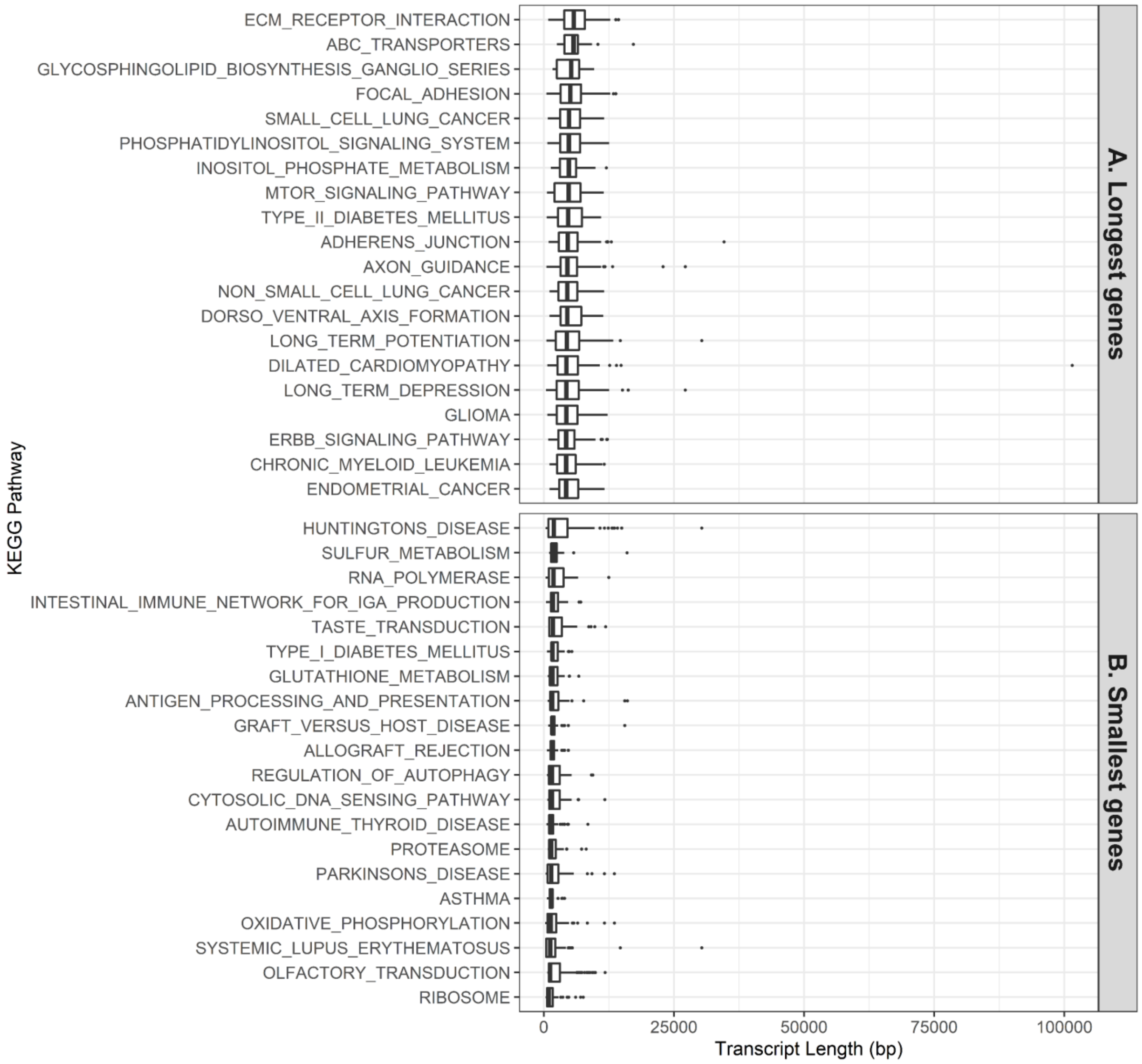

Supplementary Figure 2.
Transcript length distribution per KEGG Pathway for the APPRIS dataset. KEGG Pathways and genes involved in said pathways were obtained from the Molecular Signature Database.
