# Supplementary material for: Gene Size Matters: An Analysis of Gene Length in the Human Genome
Source: Front Genet. 2021 Feb 11;12:559998. doi: 10.3389/fgene.2021.559998 (PMC7905317; doi:10.3389/fgene.2021.559998)
Supplement: Supplementary file 9 [file Data_Sheet_3.pdf]

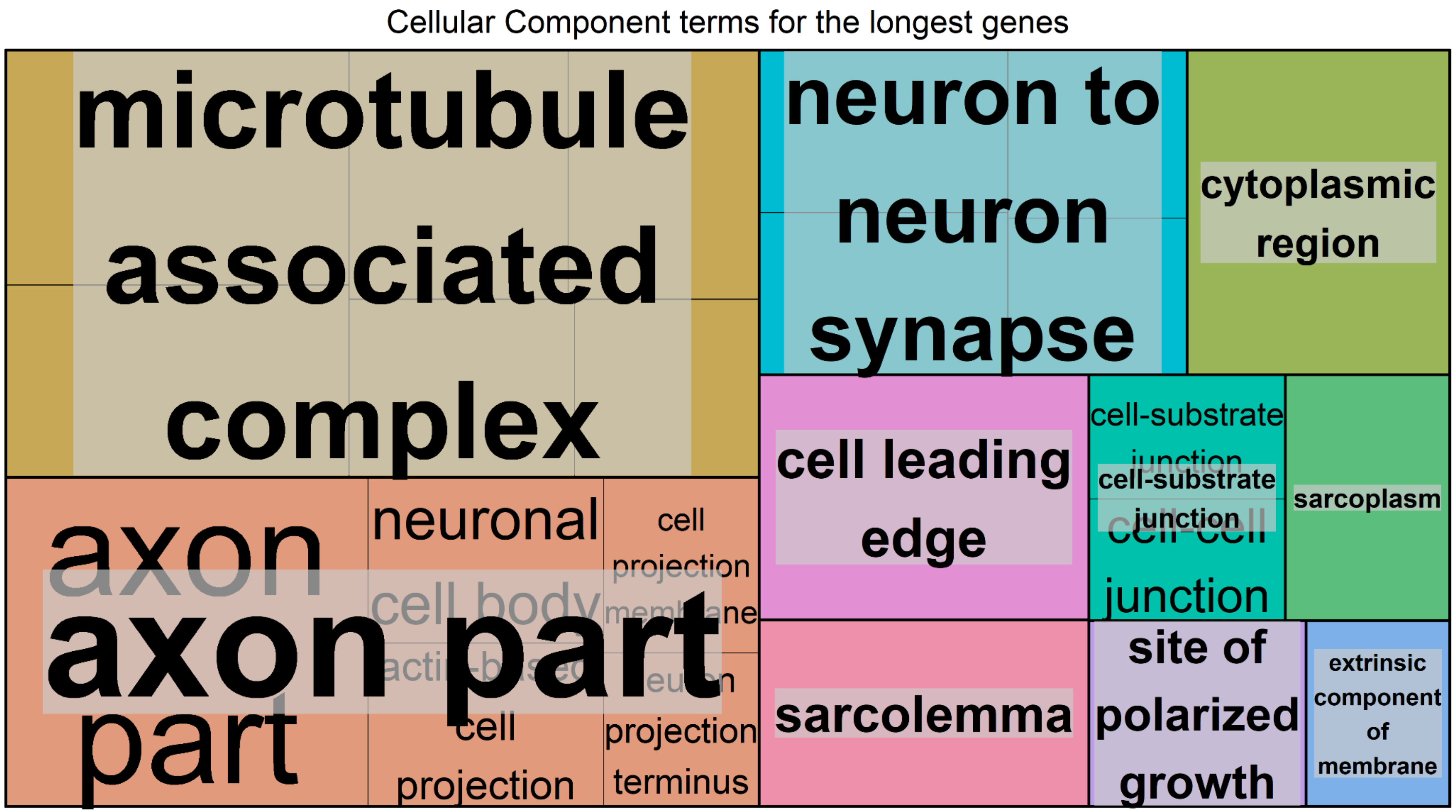

Supplementary Figure 3A.

Cellular Component terms found associated to genes with the longest transcript length (5%). The significance level was $p<0.05$ and the FDR was set at 0.05. FDR estimation was done using the Benjamini–Hochberg method.

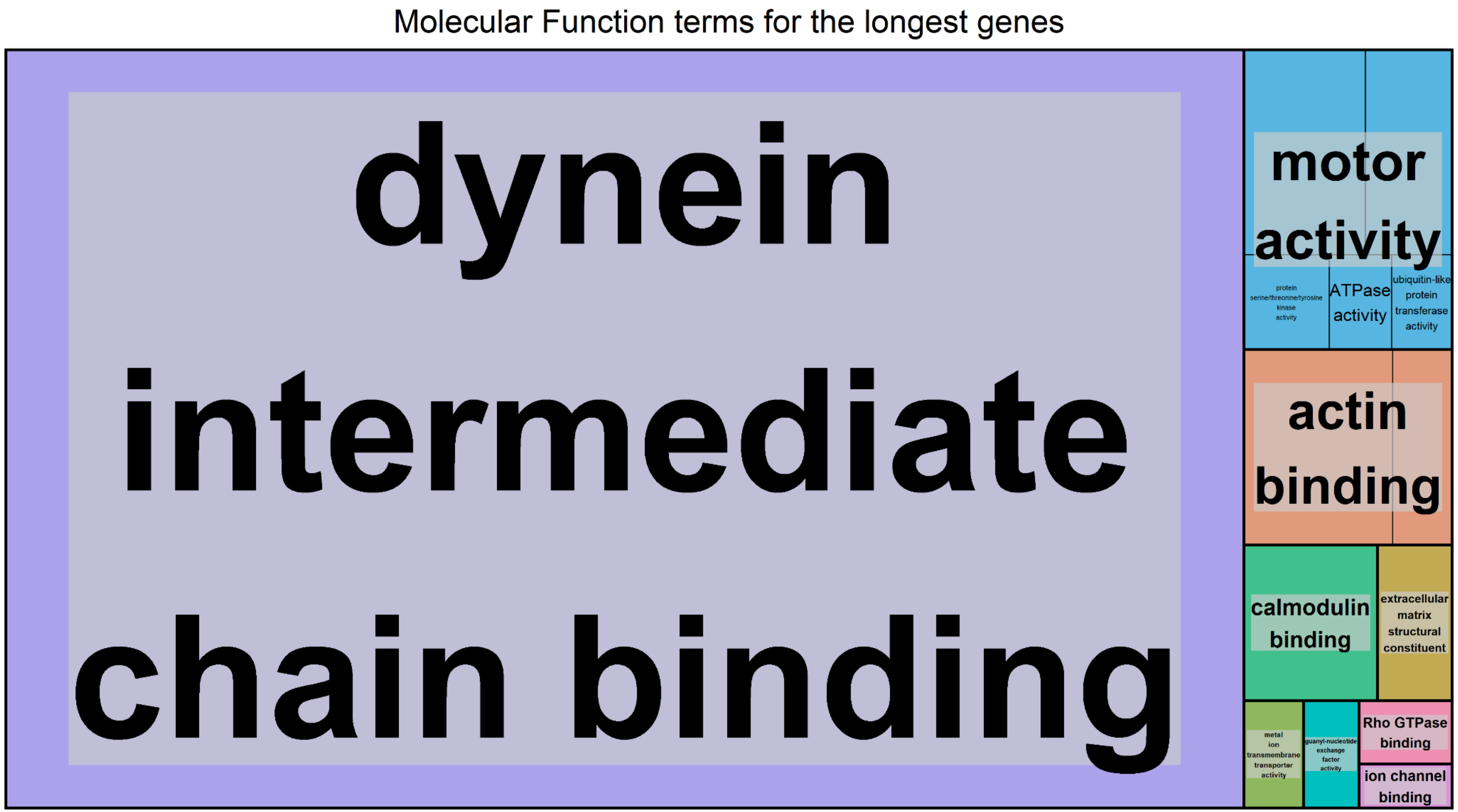

Supplementary Figure 3B.
Molecular Function terms found associated to genes with the longest transcript length (5%). The significance level was $p<0.05$ and the FDR was set at 0.05. FDR estimation was done using the Benjamini–Hochberg method.

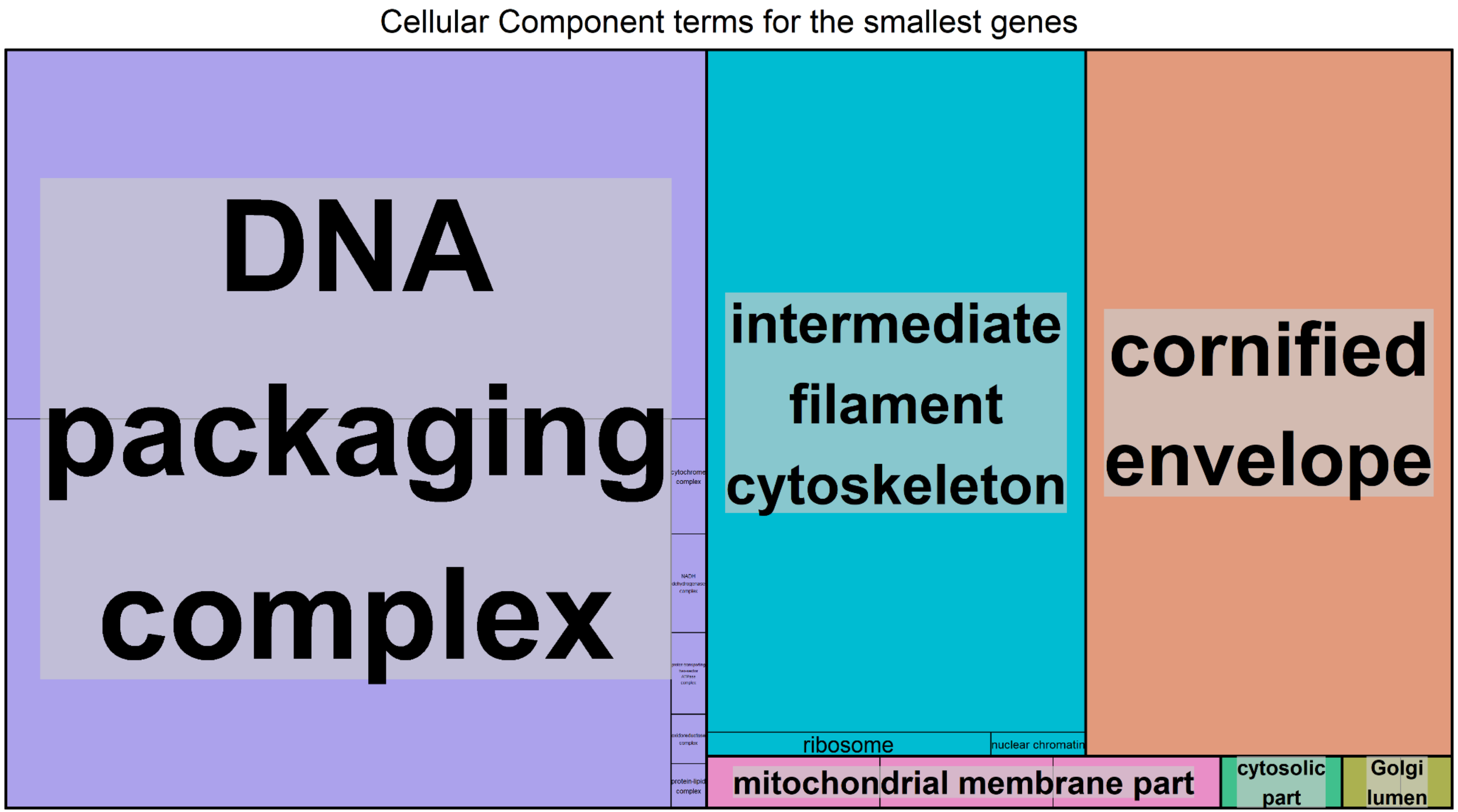

Supplementary Figure 3C.
Cellular Component terms found associated to genes with the smallest transcript length (5%). The significance level was p<0.05 and the FDR was set at 0.05. FDR estimation was done using the Benjamini–Hochberg method.

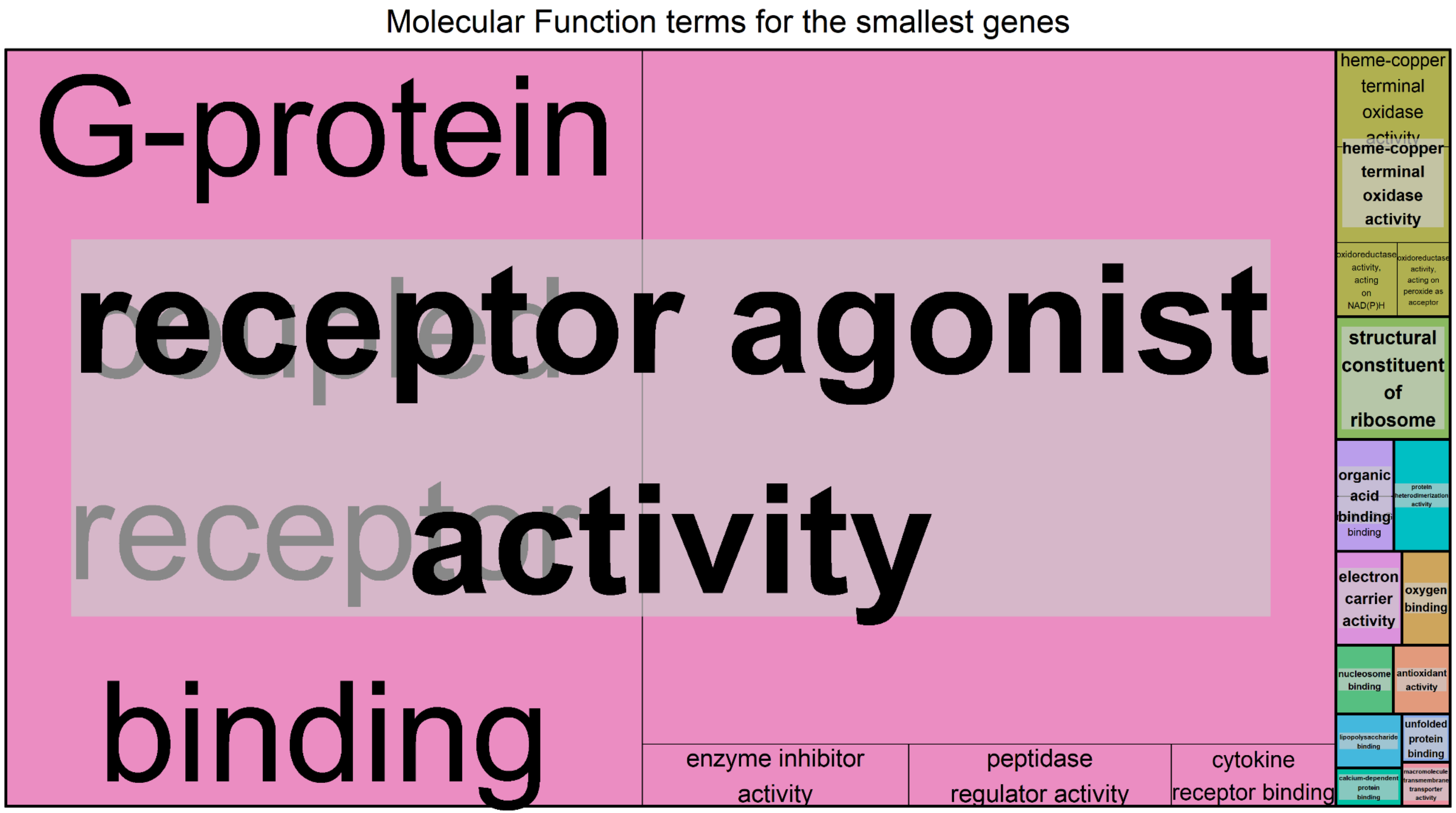

Supplementary Figure 3D.

Molecular Function terms found associated to genes with the smallest transcript length (5%). The significance level was $p<0.05$ and the FDR was set at 0.05. FDR estimation was done using the Benjamini–Hochberg method.

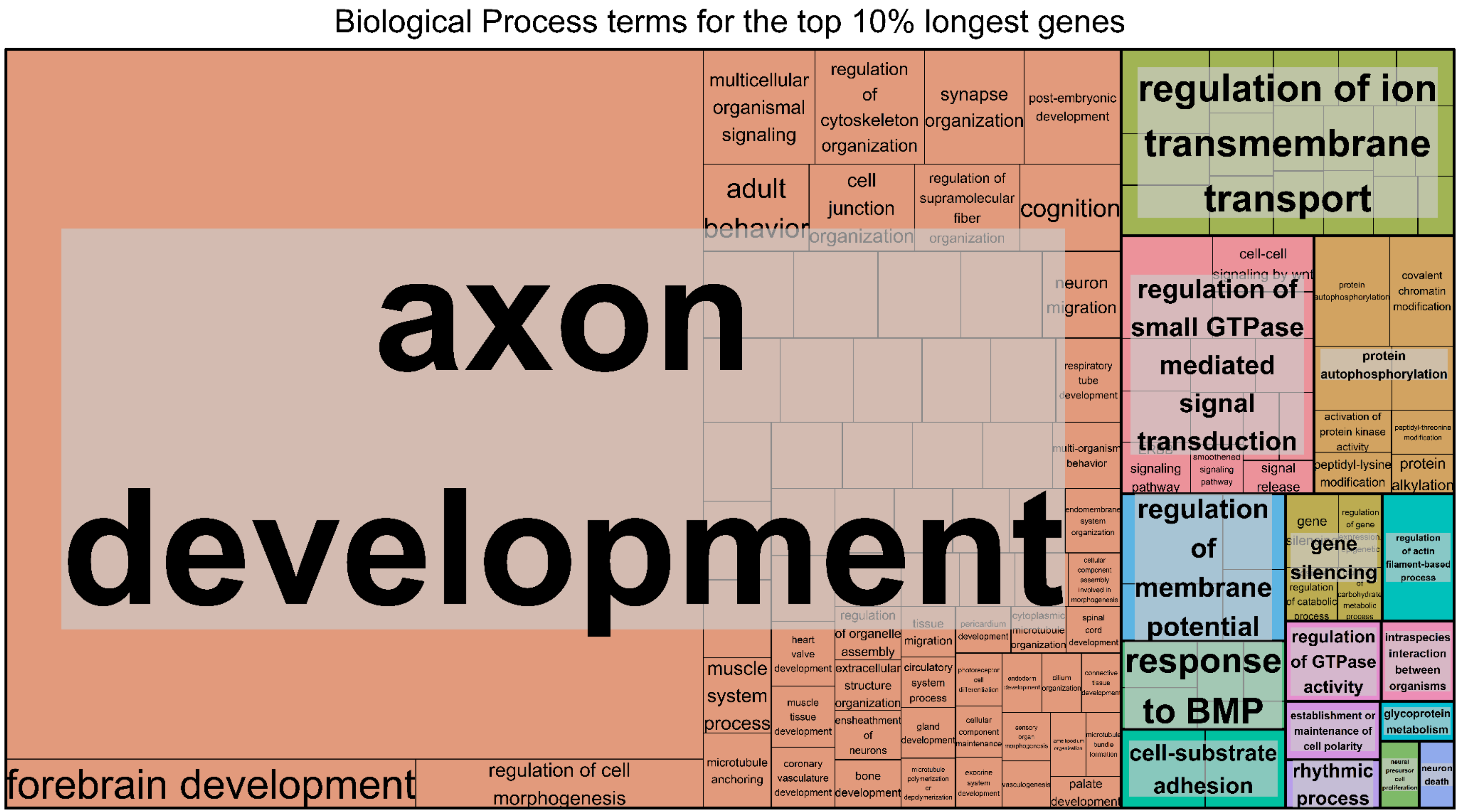

Supplementary Figure 3E.

Biological Process terms found associated to genes with the longest transcript length (10%). The significance level was p<0.05 and the FDR was set at 0.05. FDR estimation was done using the Benjamini–Hochberg method.

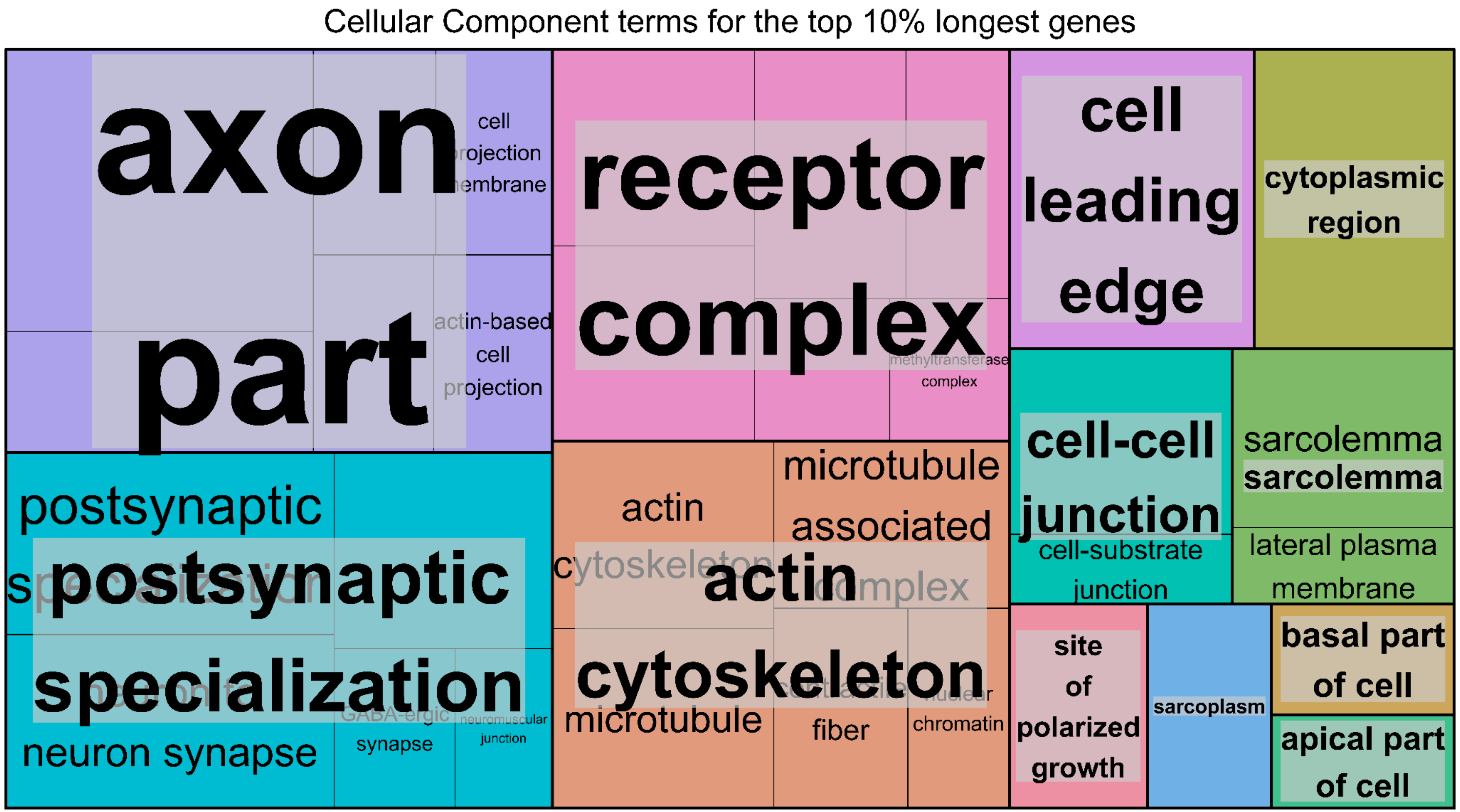

Supplementary Figure 3F.

Cellular Component terms found associated to genes with the longest transcript length (10%). The significance level was p<0.05 and the FDR was set at 0.05. FDR estimation was done using the Benjamini–Hochberg method.

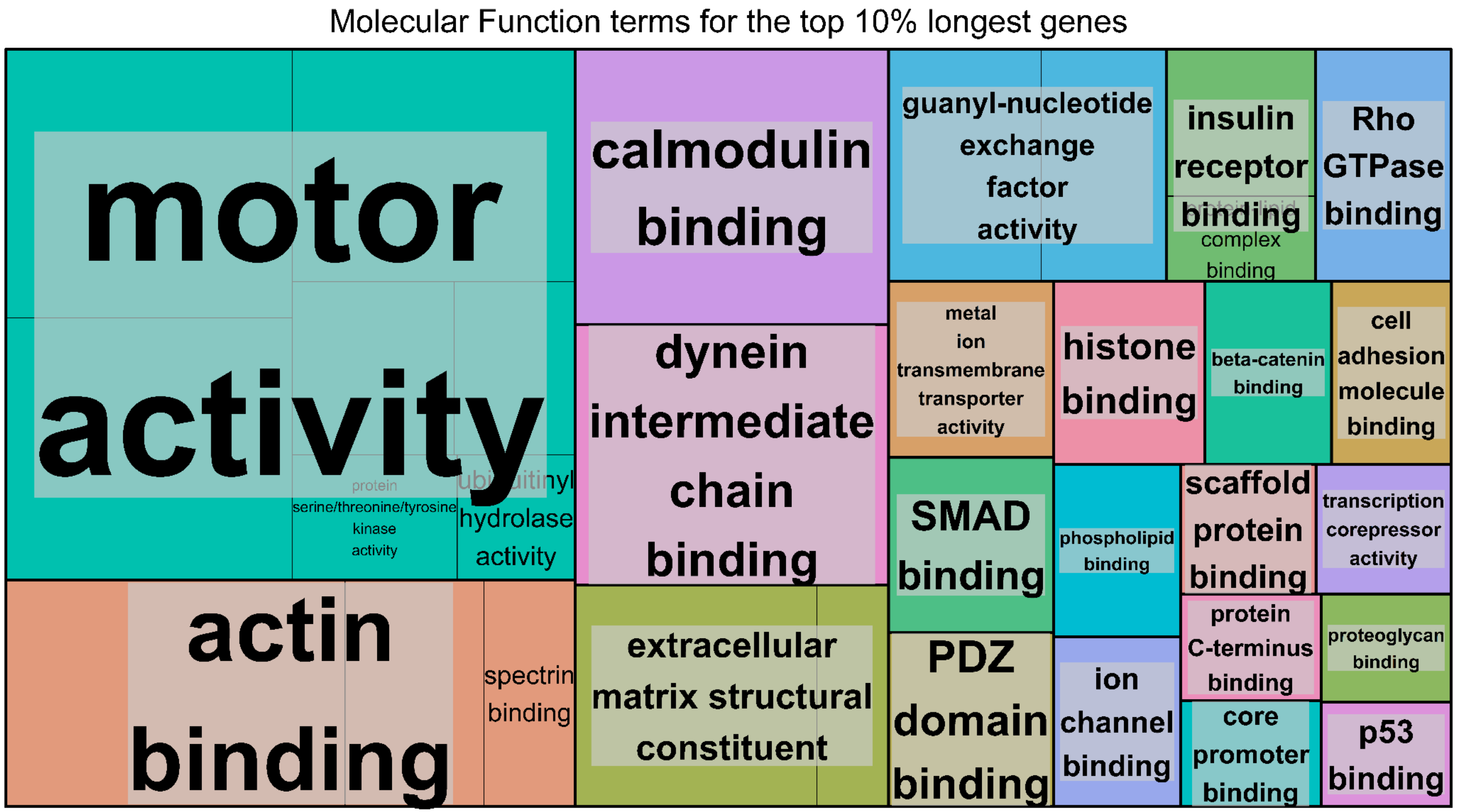

Supplementary Figure 3G.

Molecular Function terms found associated to genes with the longest transcript length (10%). The significance level was p<0.05 and the FDR was set at 0.05. FDR estimation was done using the Benjamini–Hochberg method.

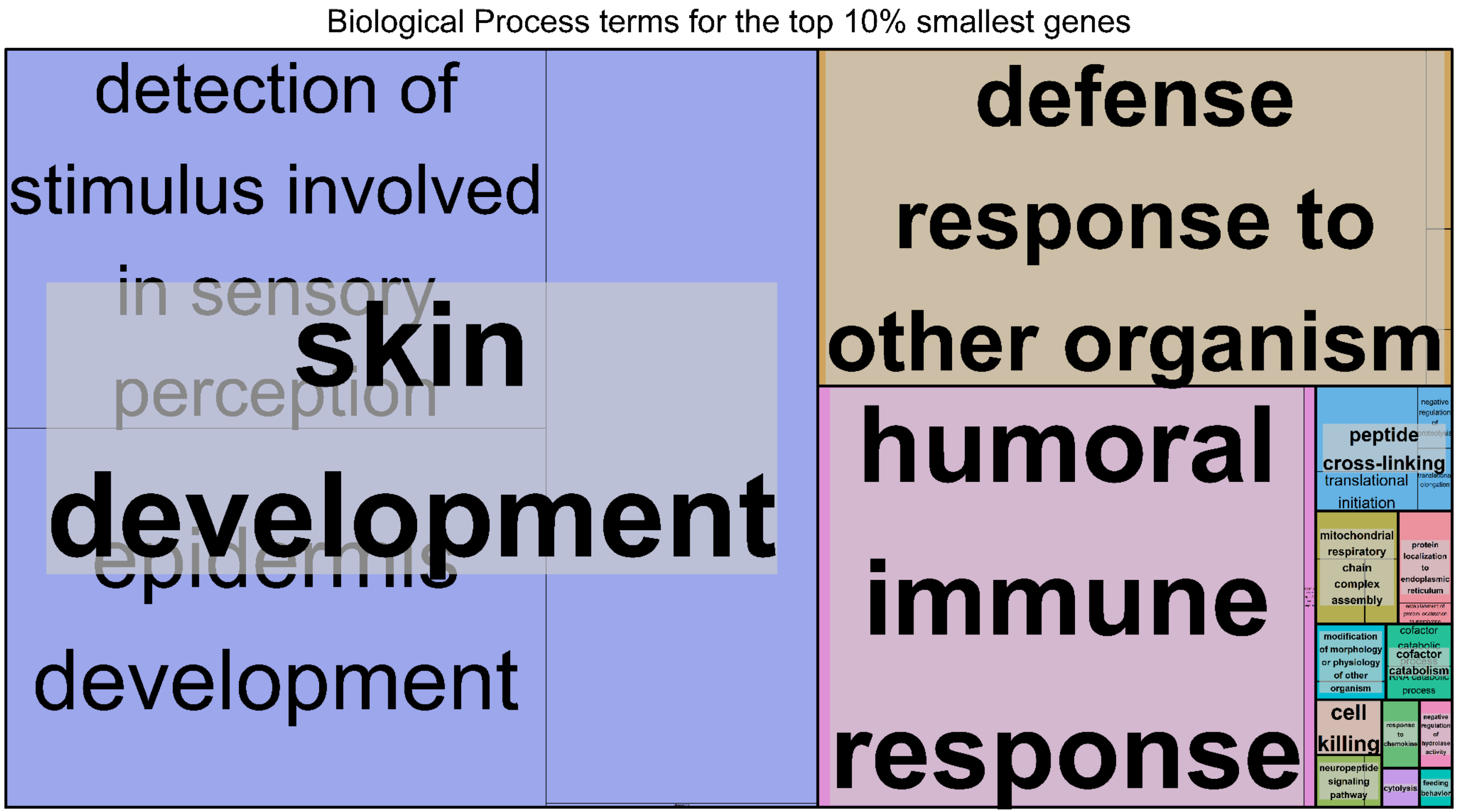

Supplementary Figure 3H.
Biological Process terms found associated to genes with the smallest transcript length (10%). The significance level was $p<0.05$ and the FDR was set at 0.05. FDR estimation was done using the Benjamini–Hochberg method.

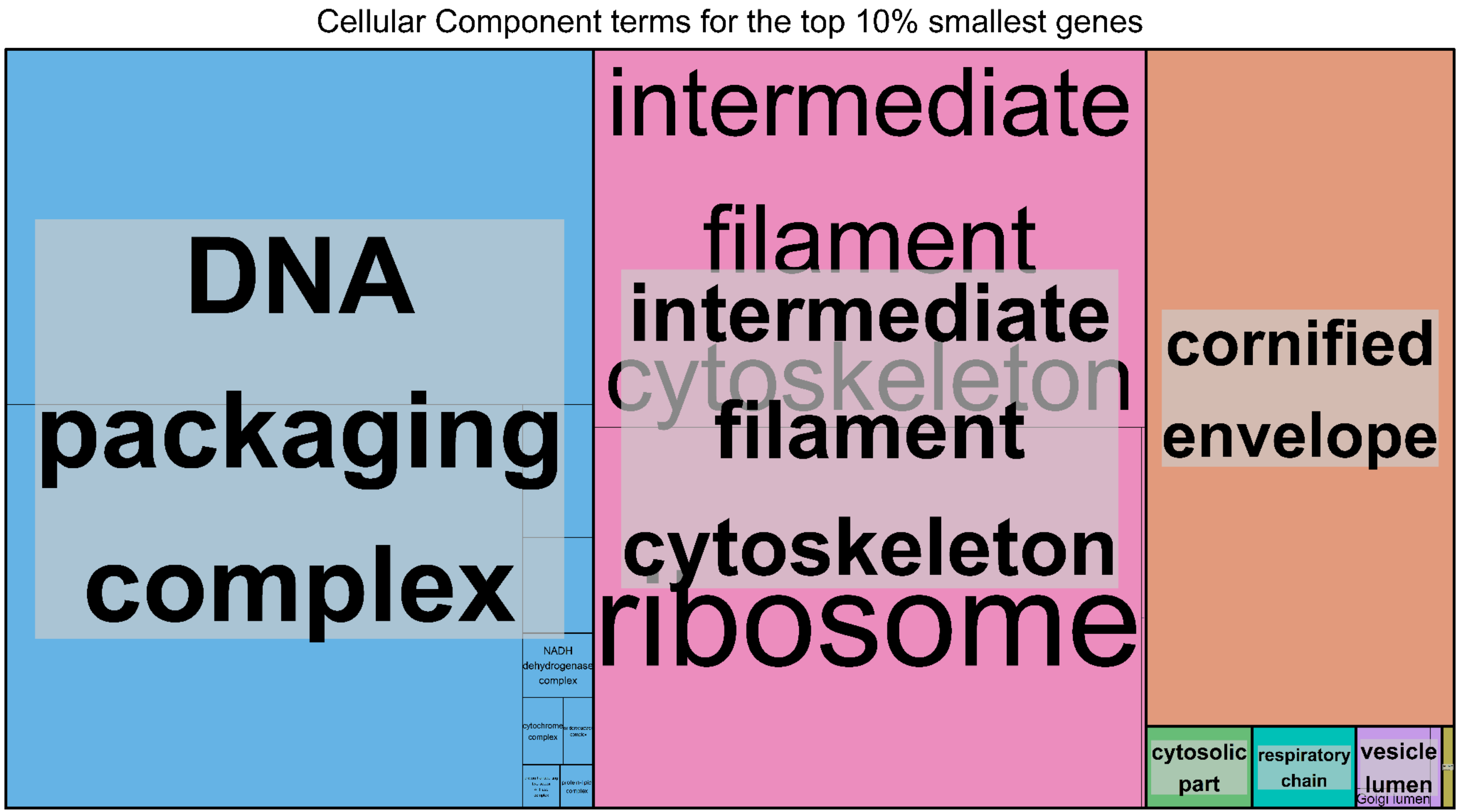

Supplementary Figure 3I.

Cellular Component terms found associated to genes with the smallest transcript length (10%). The significance level was $p<0.05$ and the FDR was set at 0.05. FDR estimation was done using the Benjamini–Hochberg method.

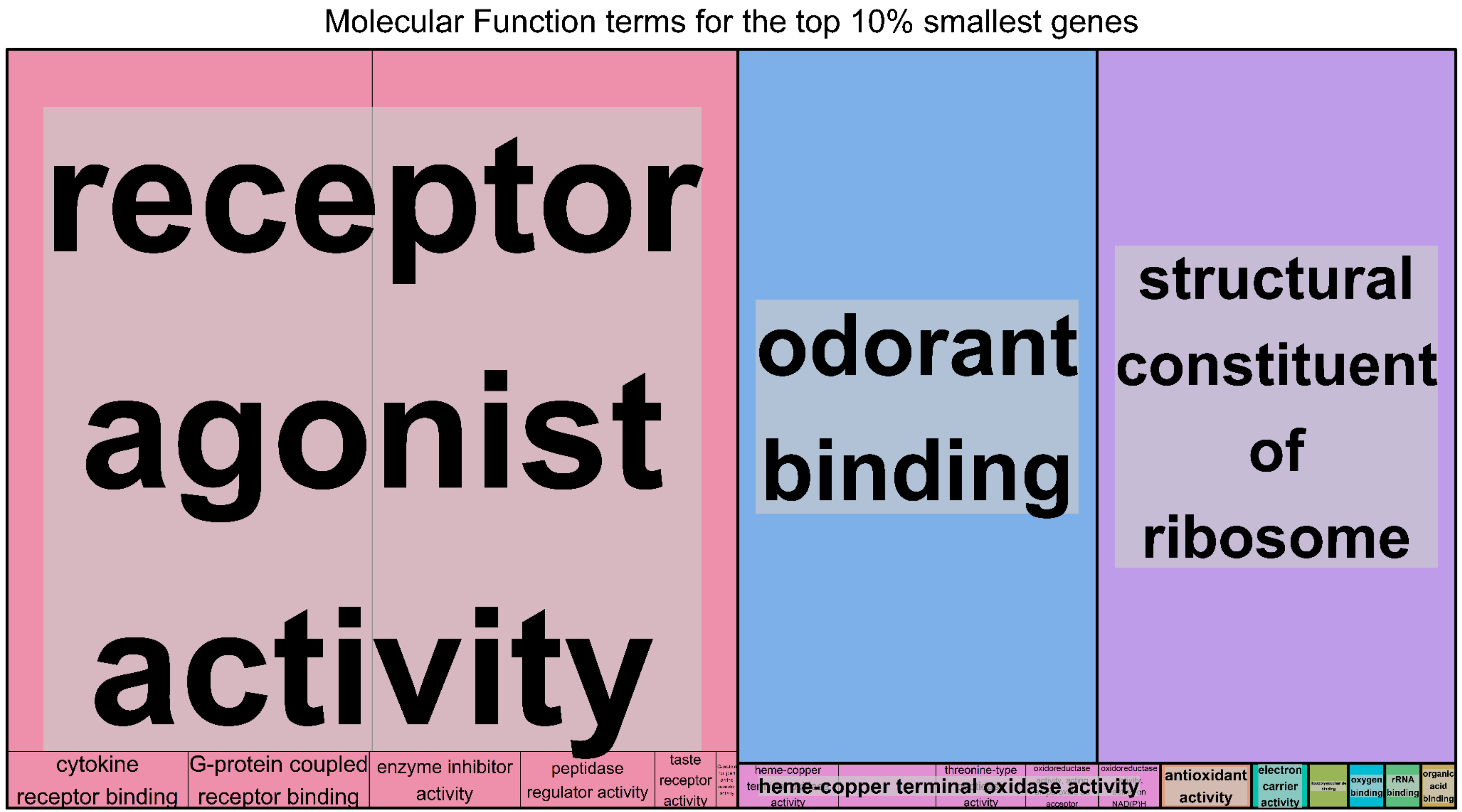

Supplementary Figure 3J.
Molecular Function terms found associated to genes with the smallest transcript length (10%). The significance level was $p<0.05$ and the FDR was set at 0.05. FDR estimation was done using the Benjamini–Hochberg method.
