# Supplementary material for: Gene Size Matters: An Analysis of Gene Length in the Human Genome
Source: Front Genet. 2021 Feb 11;12:559998. doi: 10.3389/fgene.2021.559998 (PMC7905317; doi:10.3389/fgene.2021.559998)
Supplement: Supplementary file 10 [file Data_Sheet_4.pdf]

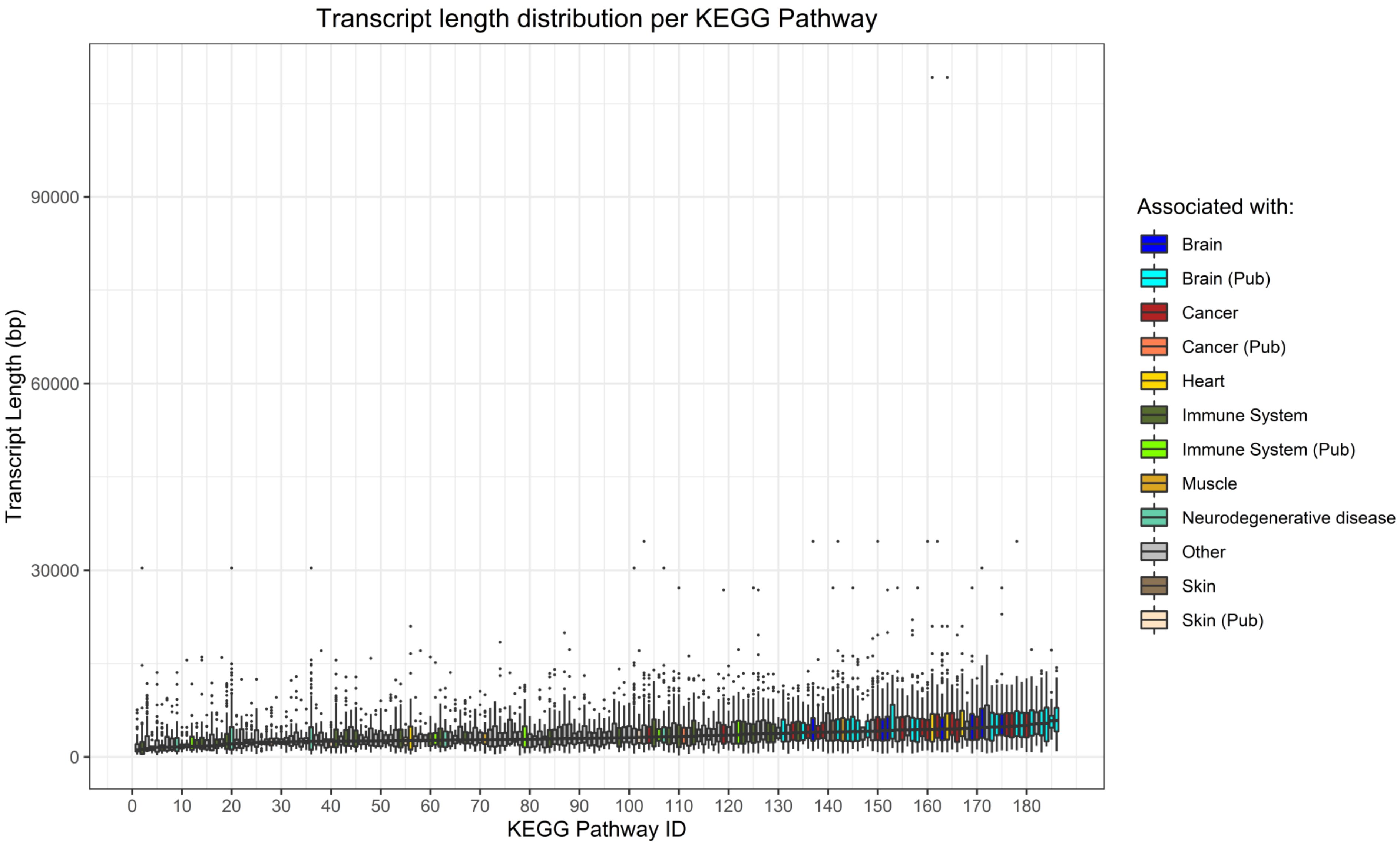

Supplementary Figure 4A.

Transcript length distribution per KEGG Pathway. Colours illustrate what the KEGG pathway has been directly associated with, due to it being stated in the pathway itself, or indirectly associated with (Pub tag), by means of literature references. KEGG Pathway IDs can be found in the Supplementary Table 4b. KEGG Pathways and genes involved in said pathways were obtained from the Molecular Signature Database.

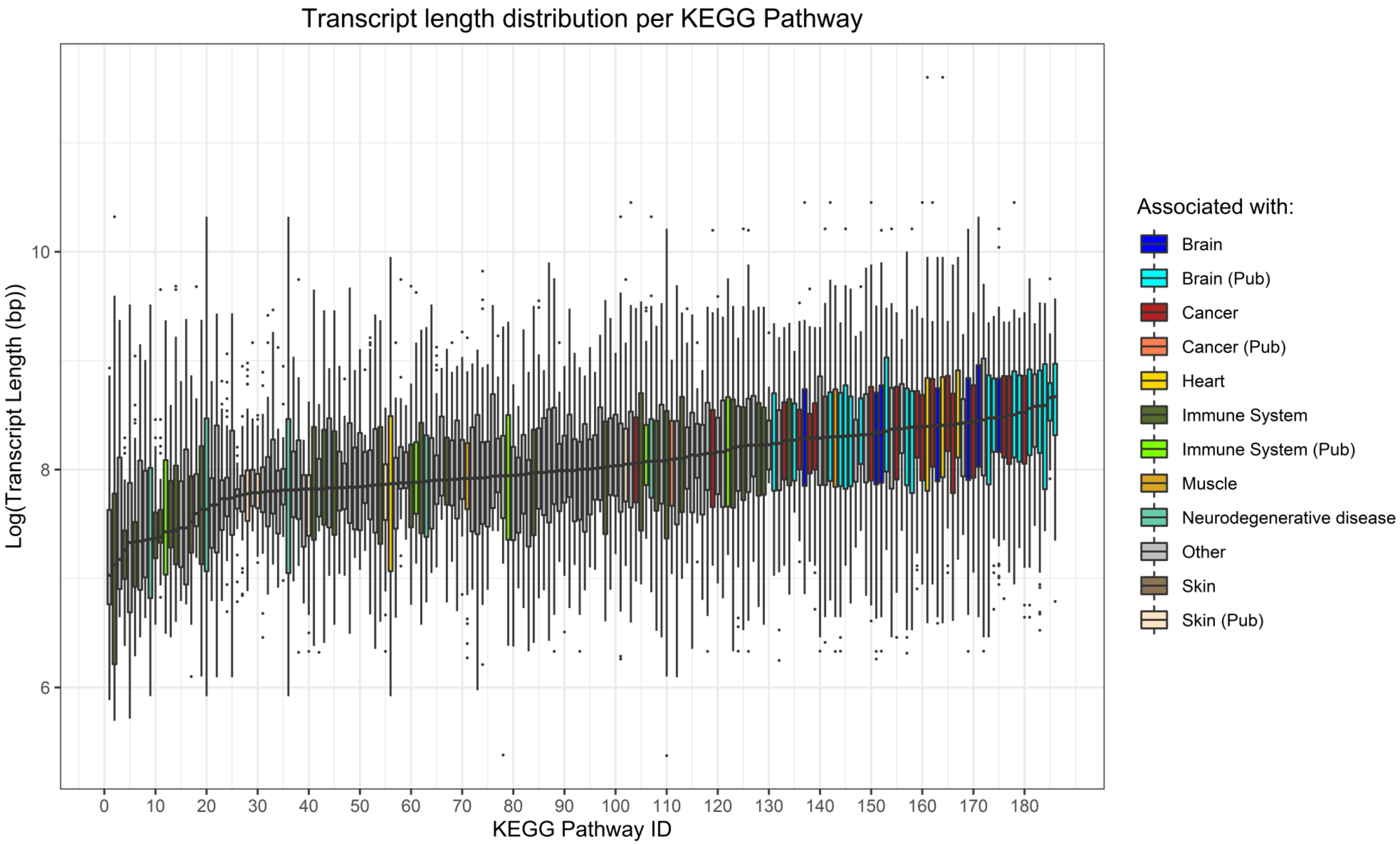

Supplementary Figure 4B.
Log transformed transcript length distribution per KEGG Pathway. Colours illustrate what the KEGG pathway has been directly associated with, due to it being stated in the pathway itself, or indirectly associated with (Pub tag), by means of literature references. KEGG Pathway IDs can be found in the Supplementary Table 4b. KEGG Pathways and genes involved in said pathways were obtained from the Molecular Signature Database.
