# Supplementary material for: Gene Size Matters: An Analysis of Gene Length in the Human Genome
Source: Front Genet. 2021 Feb 11;12:559998. doi: 10.3389/fgene.2021.559998 (PMC7905317; doi:10.3389/fgene.2021.559998)
Supplement: Supplementary file 11 [file Data_Sheet_5.pdf]

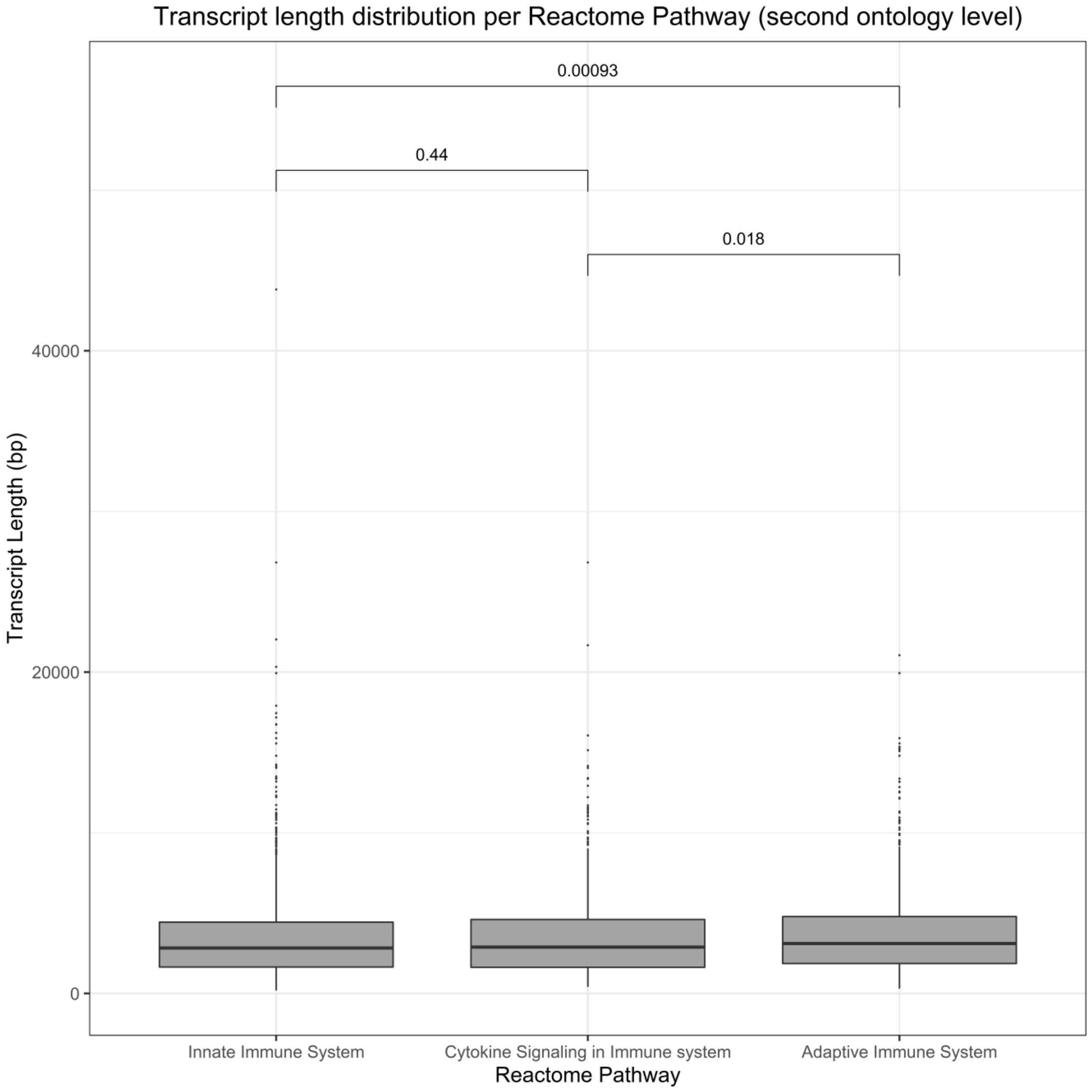

Supplementary Figure 5A.
Transcript length distribution per immune system pathway (second ontology level), from Reactome (Version 75).

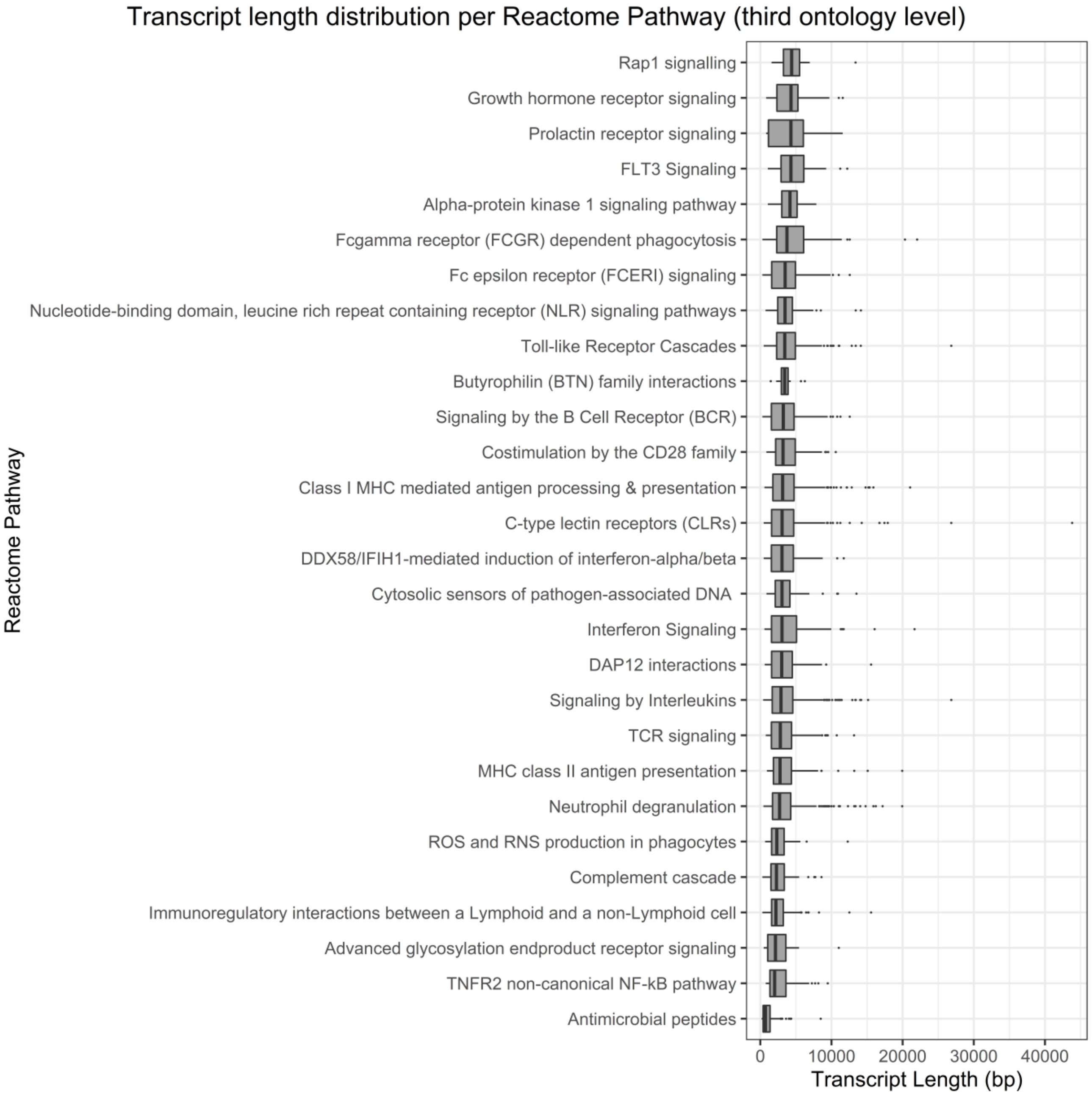

Supplementary Figure 5B.

Transcript length distribution per immune system pathway (third ontology level), from Reactome (Version 75).

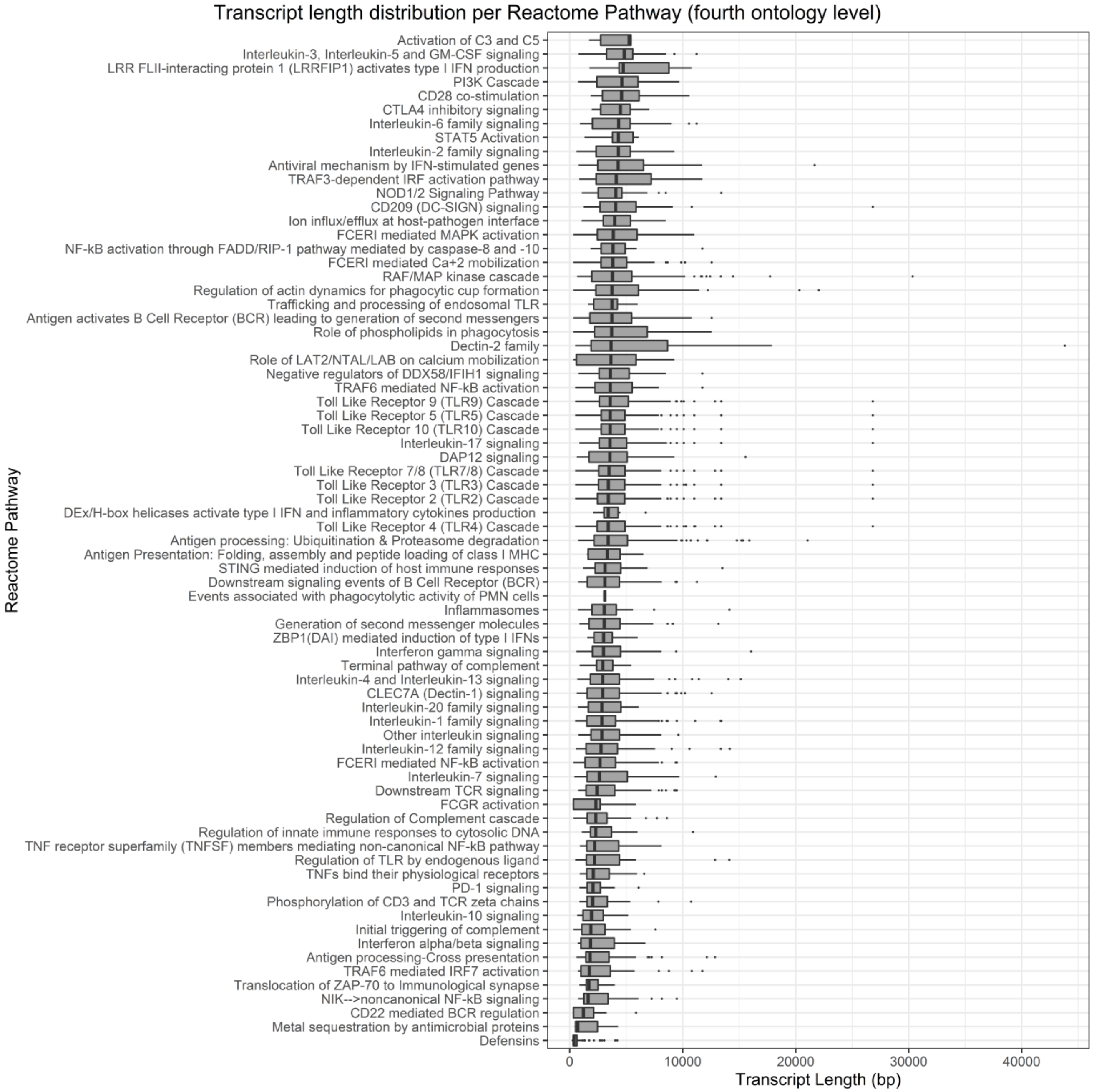

Supplementary Figure 5C.

Transcript length distribution per immune system pathway (fourth ontology level), from Reactome (Version 75).
