# Supplementary material for: Gene Size Matters: An Analysis of Gene Length in the Human Genome
Source: Front Genet. 2021 Feb 11;12:559998. doi: 10.3389/fgene.2021.559998 (PMC7905317; doi:10.3389/fgene.2021.559998)
Supplement: Supplementary file 12 [file Data_Sheet_6.pdf]

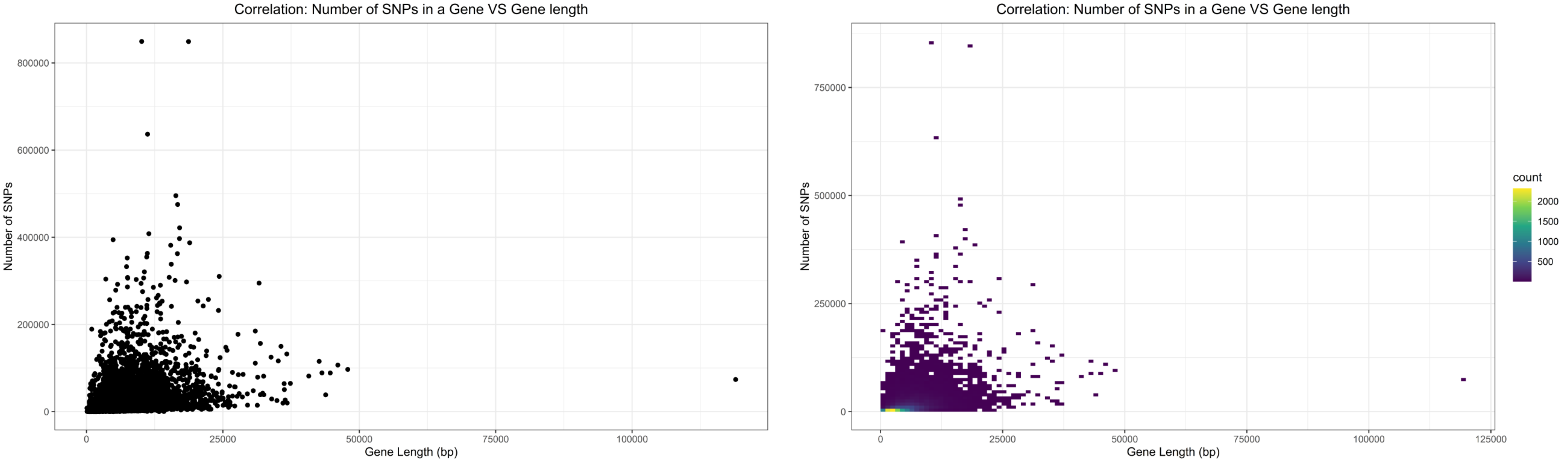

Supplementary Figure 6A.
Correlation between the Number of SNPs per gene and Gene Length and 2D density plot. Gene Length the Number of SNPs was obtained using the biomart.

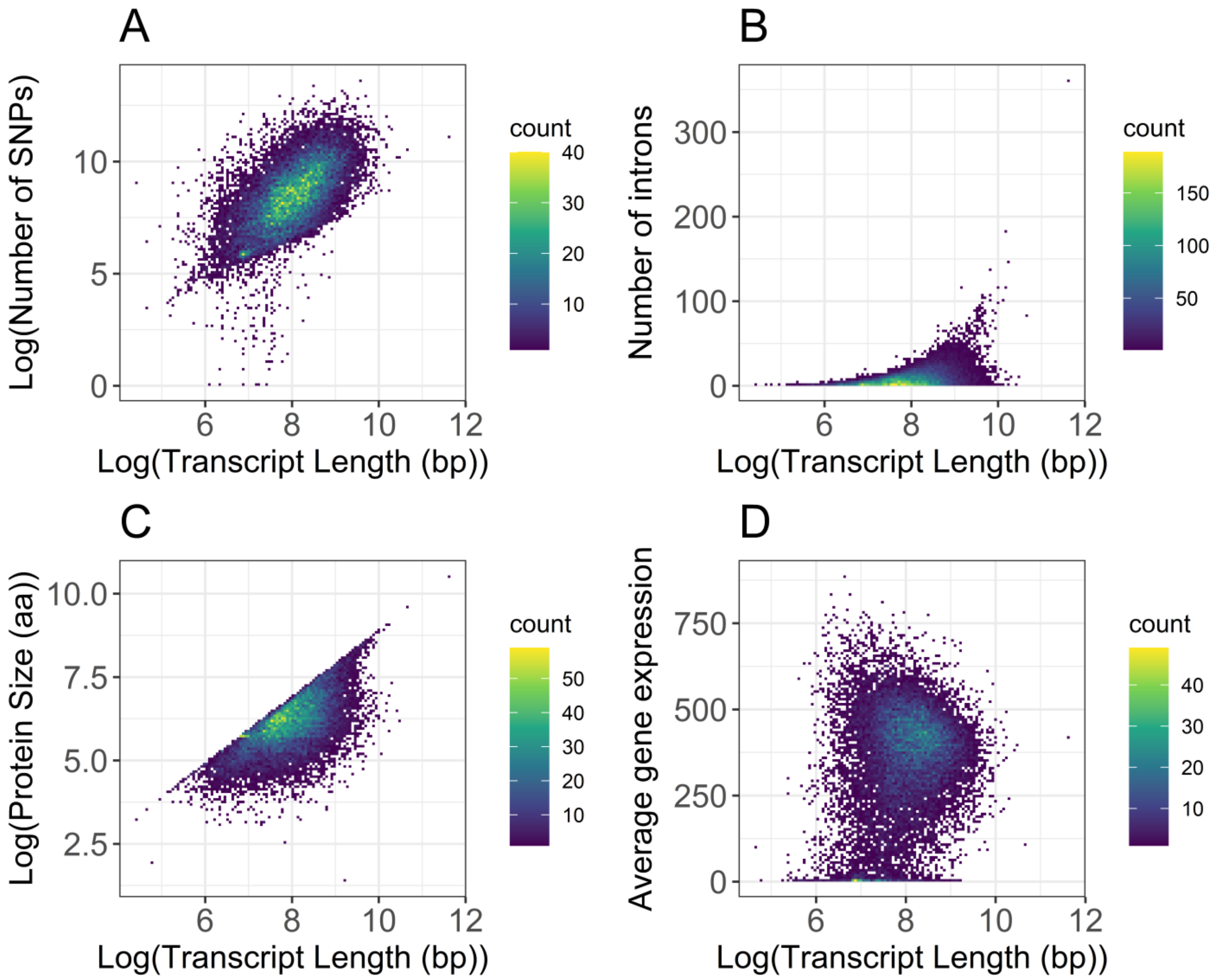

Supplementary Figure 6B.

2D density plot of the correlation analysis between Transcript Length (bp) and several other gene characteristics present in Fig 4. A: Correlation with the log transformed number of SNPs; B: Correlation with the log transformed number of introns; C: Correlation with the log transformed protein size; D: Correlation with the log transformed average gene expression.

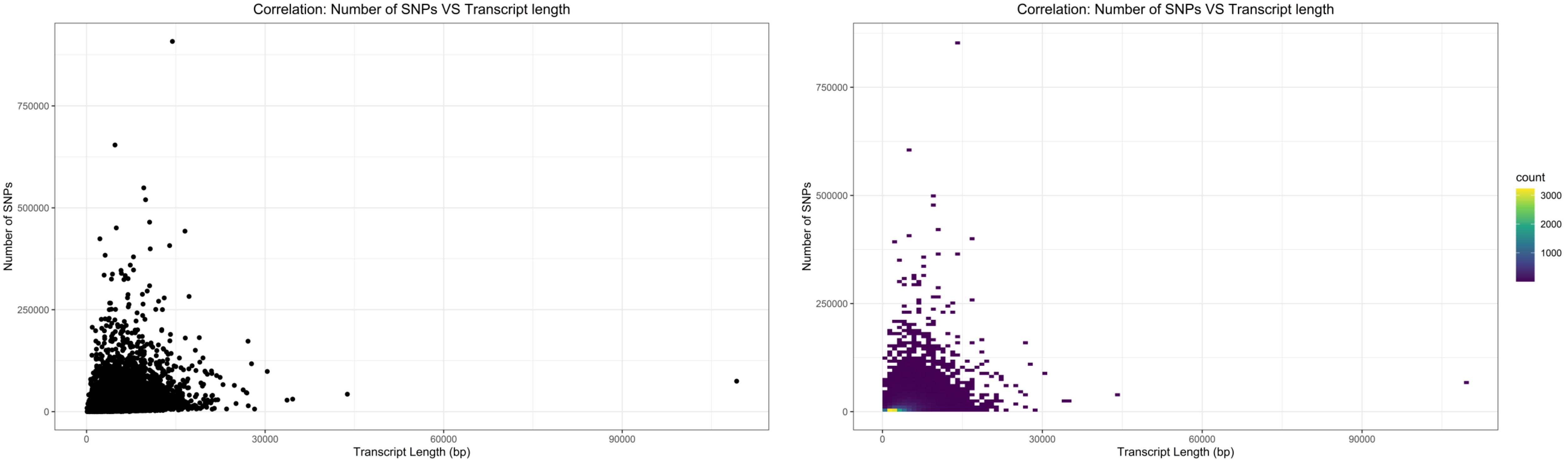

Supplementary Figure 6C.
Correlation between the Number of SNPs and Transcript Length and 2D density plot. Number of SNPs and Transcript Length were obtained using biomart.

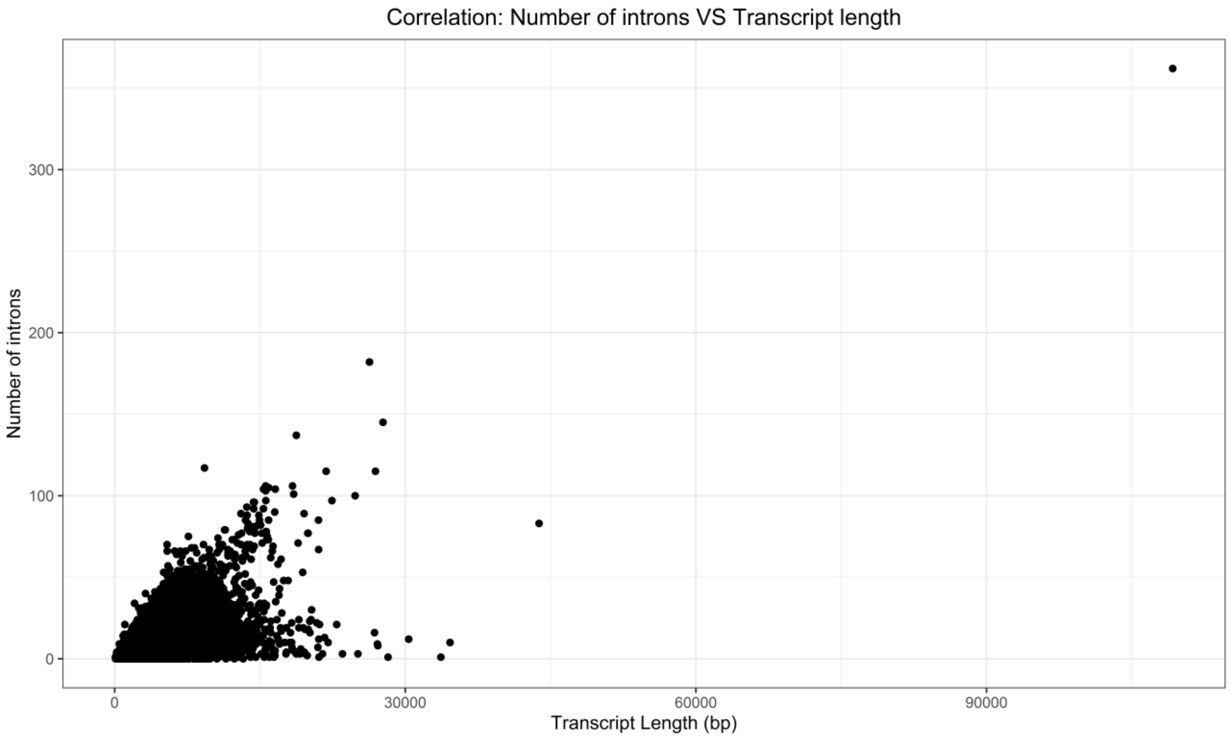

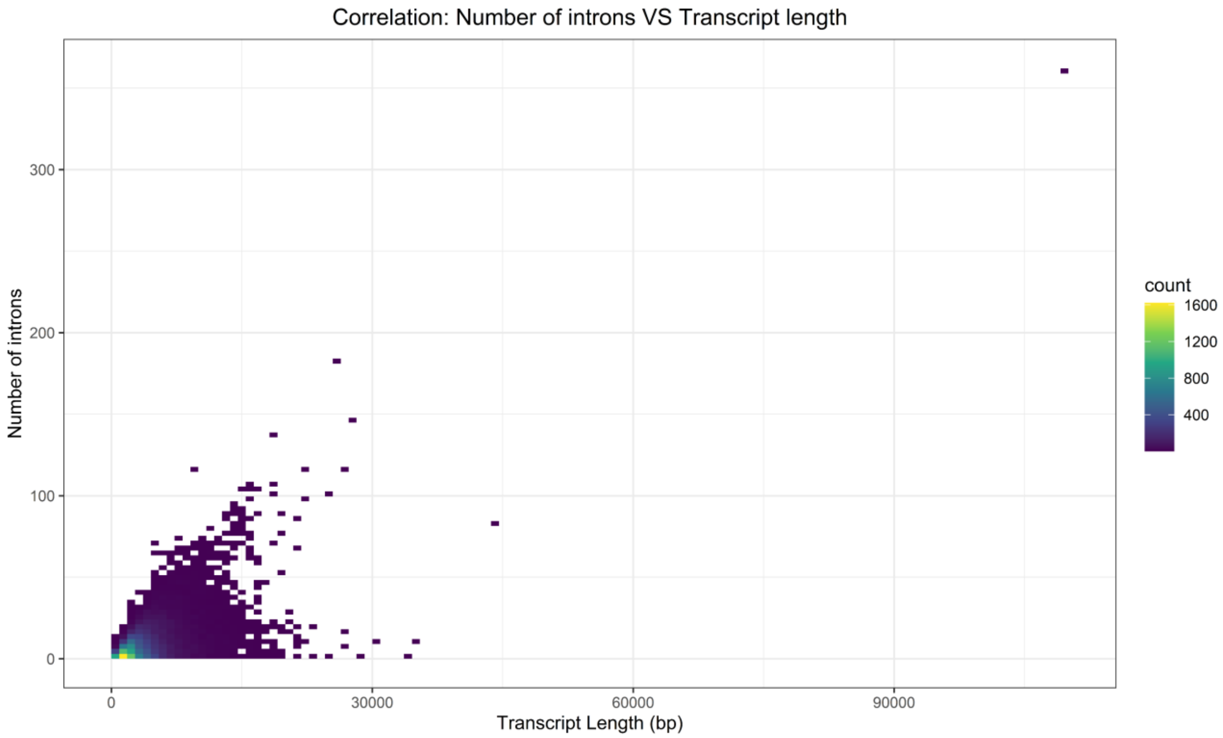

Supplementary Figure 6D.
Correlation between the number of introns and Transcript Length and 2D density plot. Number of introns and Transcript Length were obtained using biomart.

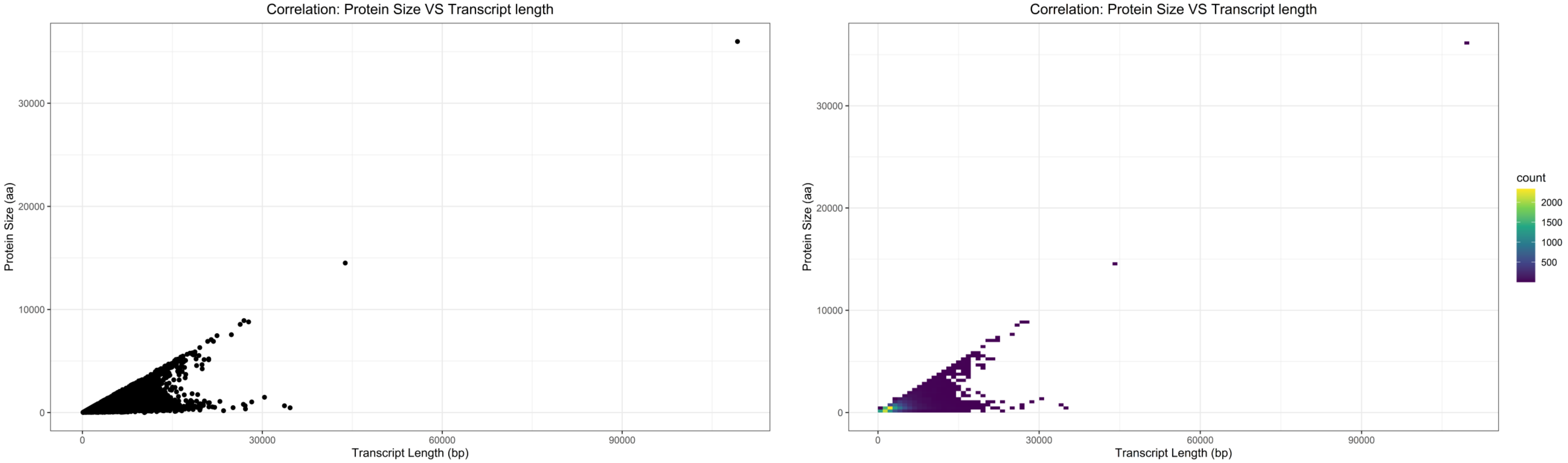

Supplementary Figure 6E.
Correlation between Protein size and Transcript Length and 2D density plot. Protein size and Transcript Length were obtained using biomart.

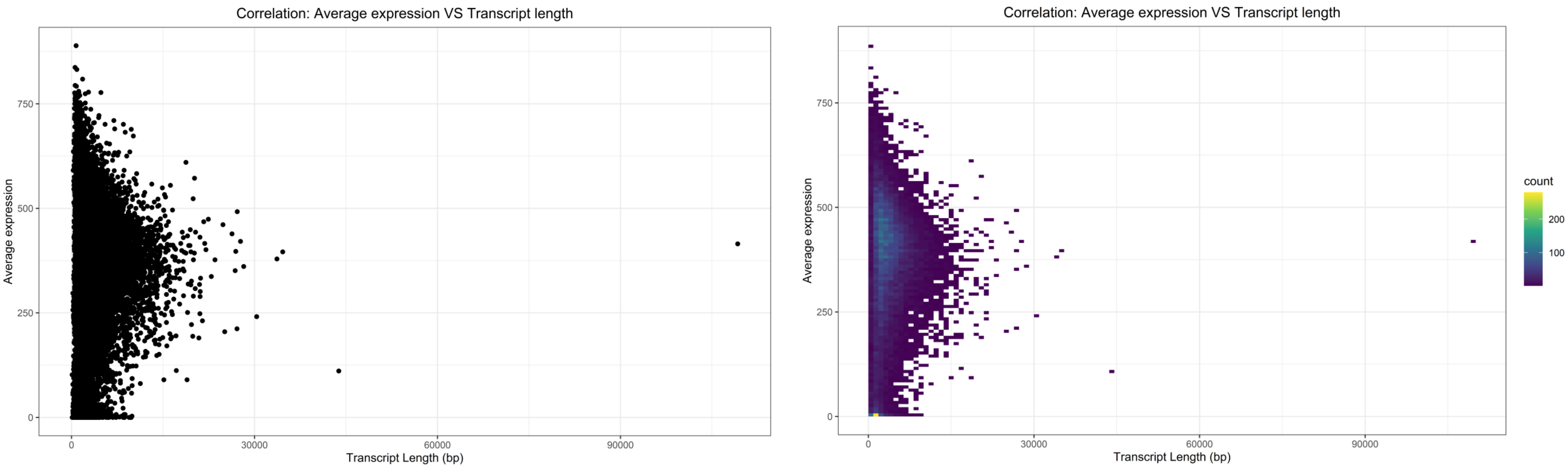

Supplementary Figure 6F.
Correlation between the Average Gene Expression and Transcript Length and 2D density plot. Average Gene Expression was obtained from the UCSC Genome browser and Transcript Length was obtained using biomart.

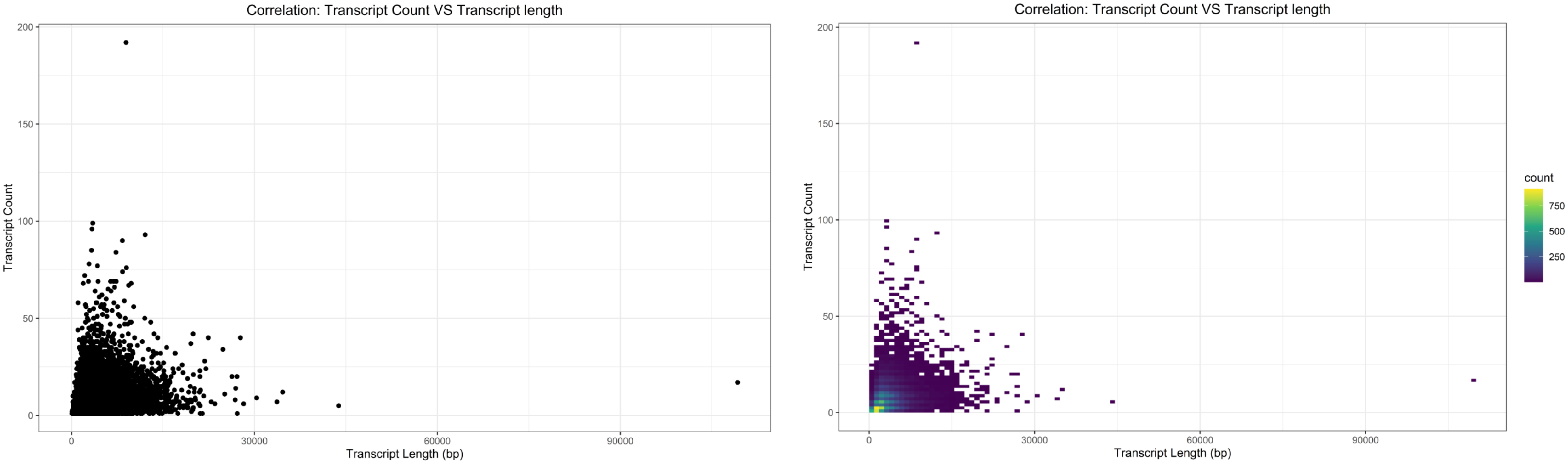

Supplementary Figure 6G.
Correlation between Transcript count and Transcript Length (bp) (Kendall test, tau = 0.22, p-value < 2.20E-16) and 2D density plot. Transcript count and Transcript Length for each transcript were obtained using biomart.

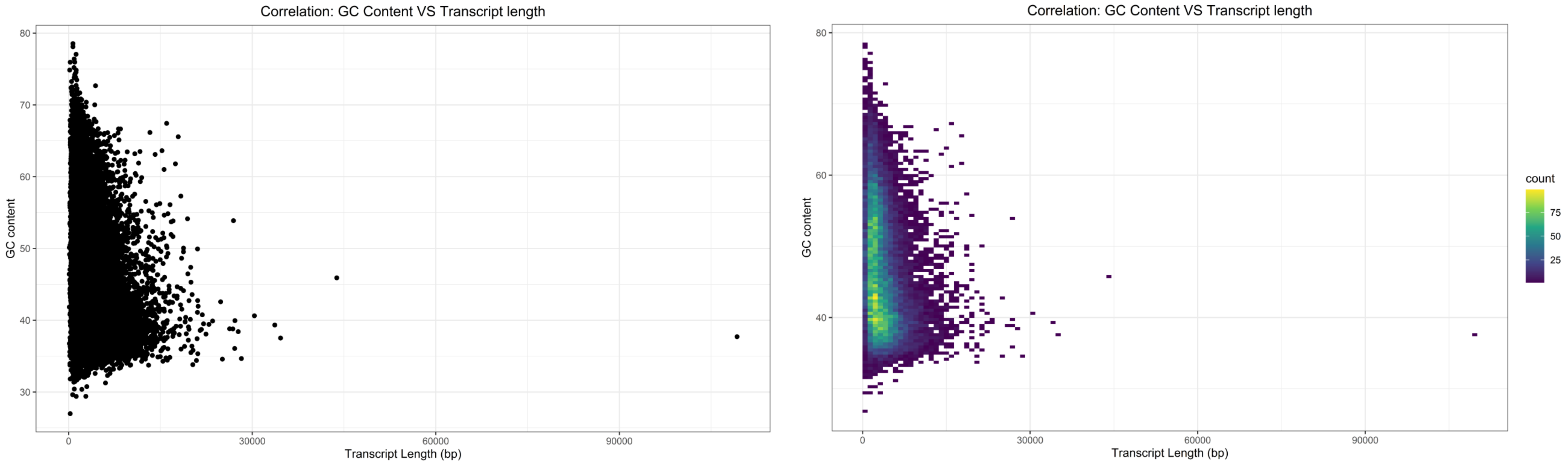

Supplementary Figure 6H.
Correlation between the GC Content and Transcript Length (bp) (Kendall test, tau = -0.19, p-value < 2.20E-16) and 2D density plot. GC Content and Transcript Length were obtained using biomart.

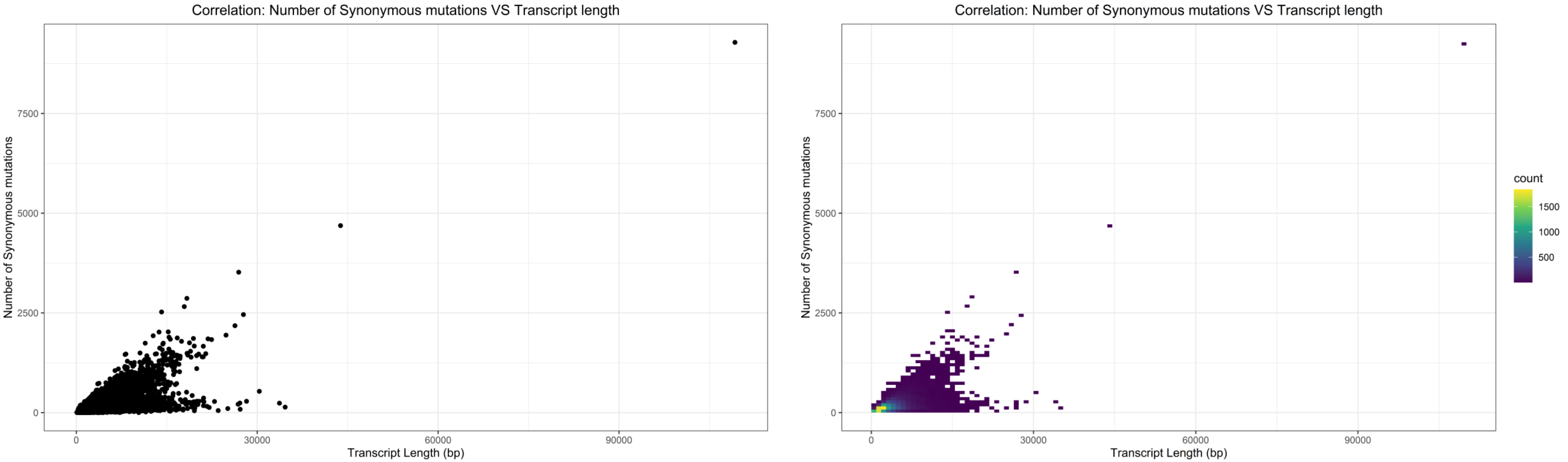

Supplementary Figure 6I.
Correlation between synonymous mutations and Transcript Length (bp) (Kendall test, tau = 0.44, p-value < 2.20E-16) and 2D density plot. The number of synonymous mutations and Transcript Length were obtained using biomart.

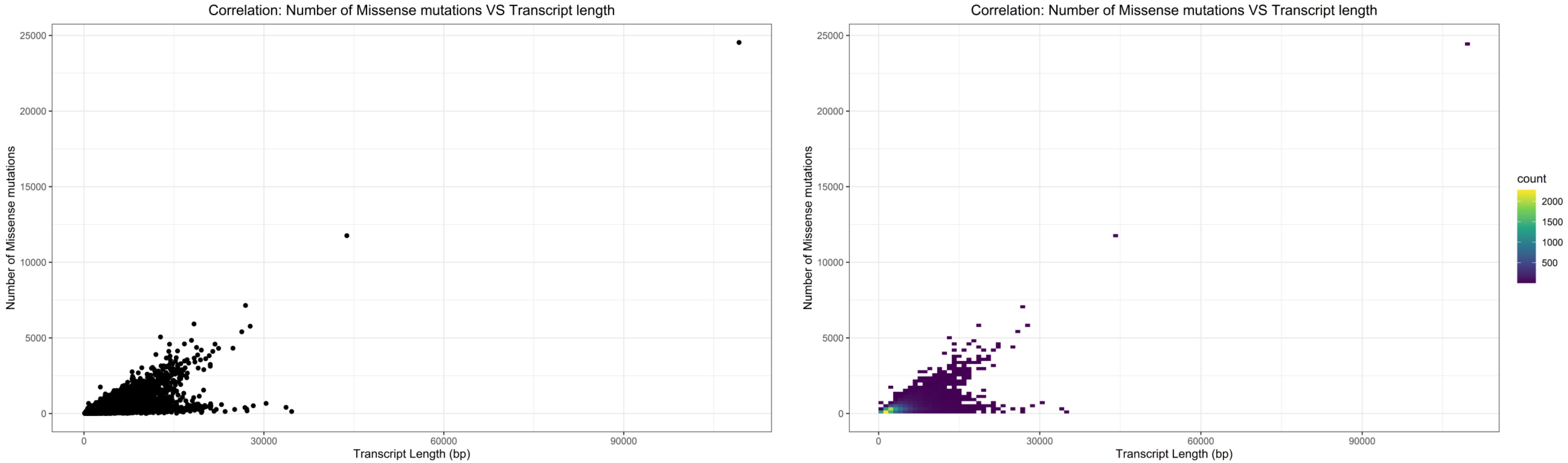

Supplementary Figure 6J.

Correlation between missense mutations and Transcript Length (bp) (Kendall test, tau = 0.42, p-value < 2.20E-16) and 2D density plot. The number of missense mutations and Transcript Length were obtained using biomart.

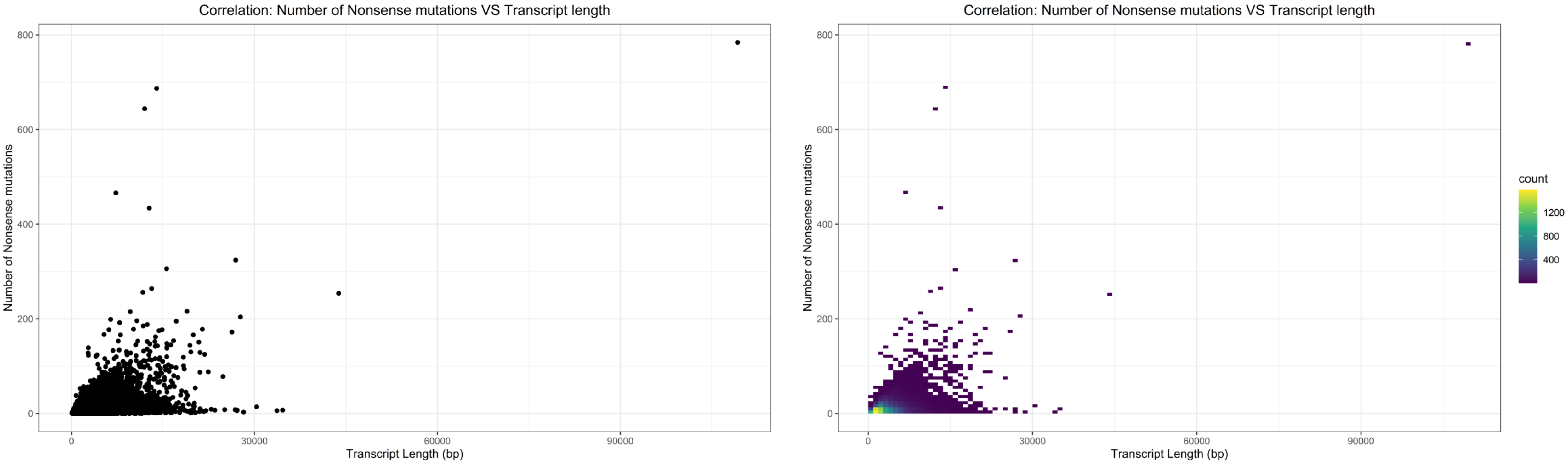

Supplementary Figure 6K.
Correlation between nonsense mutations and Transcript Length (bp) (Kendall test, tau = 0.21, p-value < 2.20E-16) and 2D density plot. The number of nonsense mutations and Transcript Length were obtained using biomart.

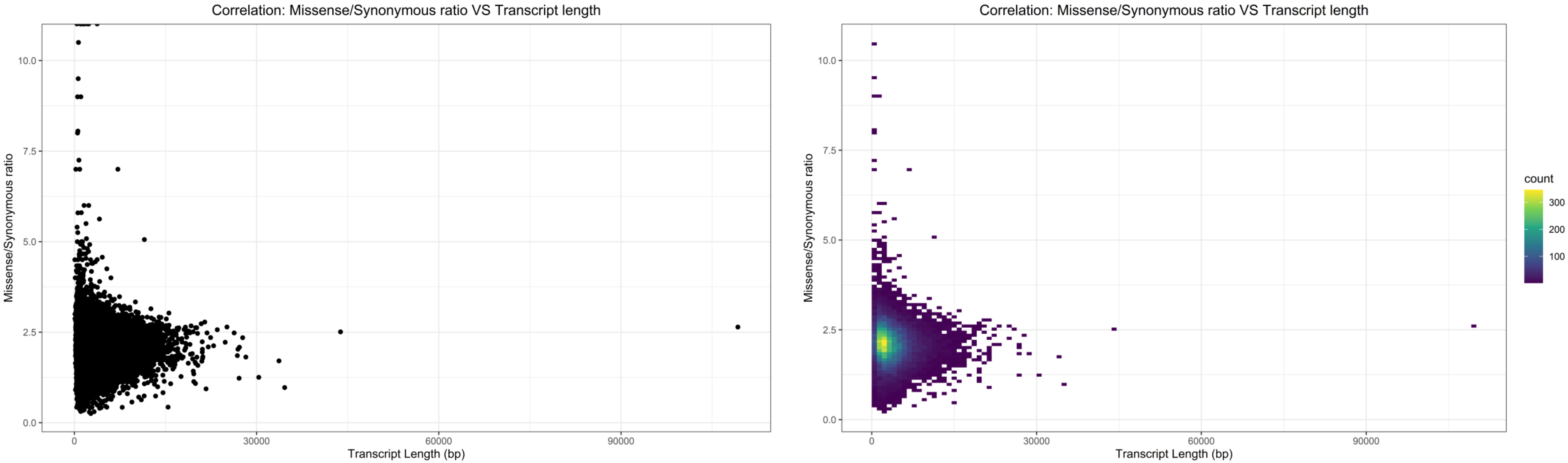

Supplementary Figure 6L.
Correlation between the missense/synonymous ratio of mutations and Transcript Length (bp) (Kendall test, tau = -0.07, p-value < 2.20E-16) and 2D density plot. The number of missense and synonymous mutations and Transcript Length were obtained using biomart.

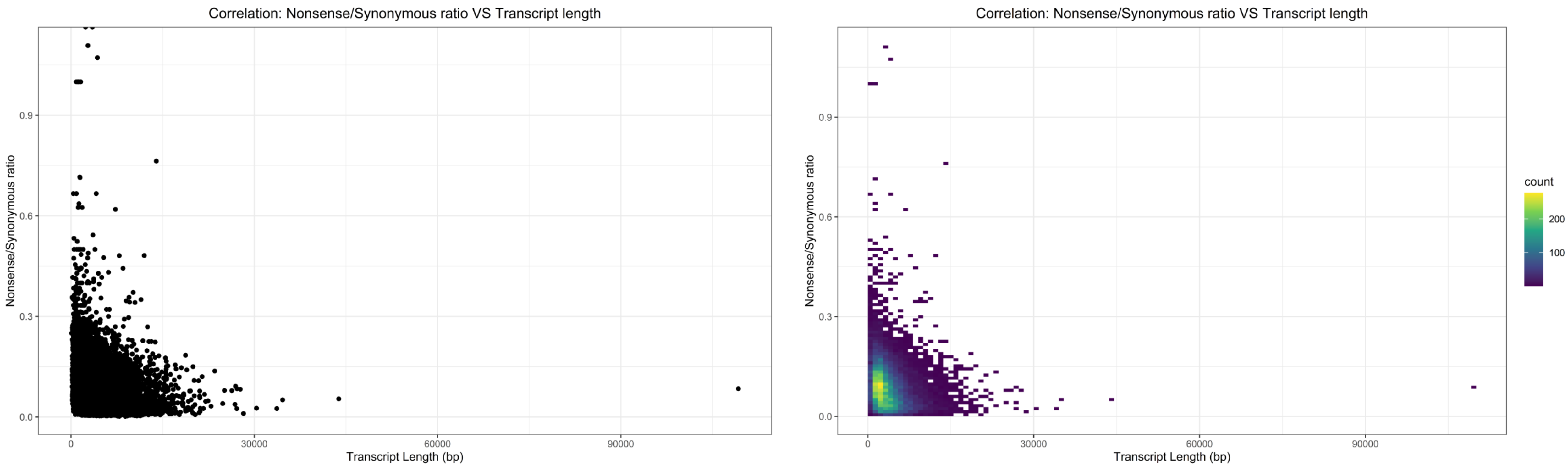

Supplementary Figure 6M.
Correlation between the nonsense/synonymous ratio of mutations and Transcript Length (bp) (Kendall test, tau = -0.19, p-value < 2.20E-16) and 2D density plot. The number of nonsense and synonymous mutations and Transcript Length were obtained using biomart.
