# Supplementary material for: Gene Size Matters: An Analysis of Gene Length in the Human Genome
Source: Front Genet. 2021 Feb 11;12:559998. doi: 10.3389/fgene.2021.559998 (PMC7905317; doi:10.3389/fgene.2021.559998)
Supplement: Supplementary file 13 [file Data_Sheet_7.pdf]

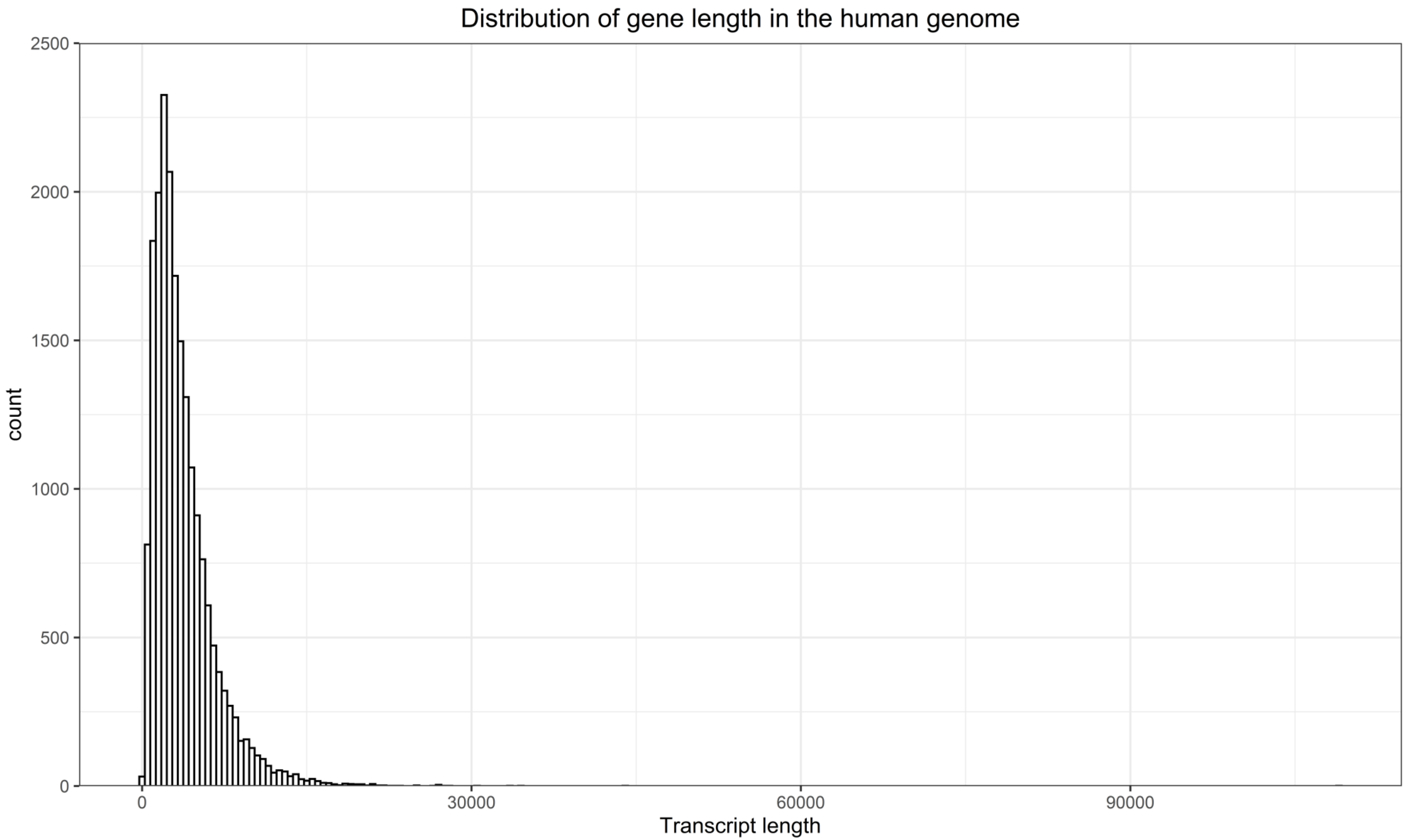

Supplementary Figure 7A.

Gene Length distribution in the human genome, for protein-coding genes only. Transcript Length was obtained from biomart.

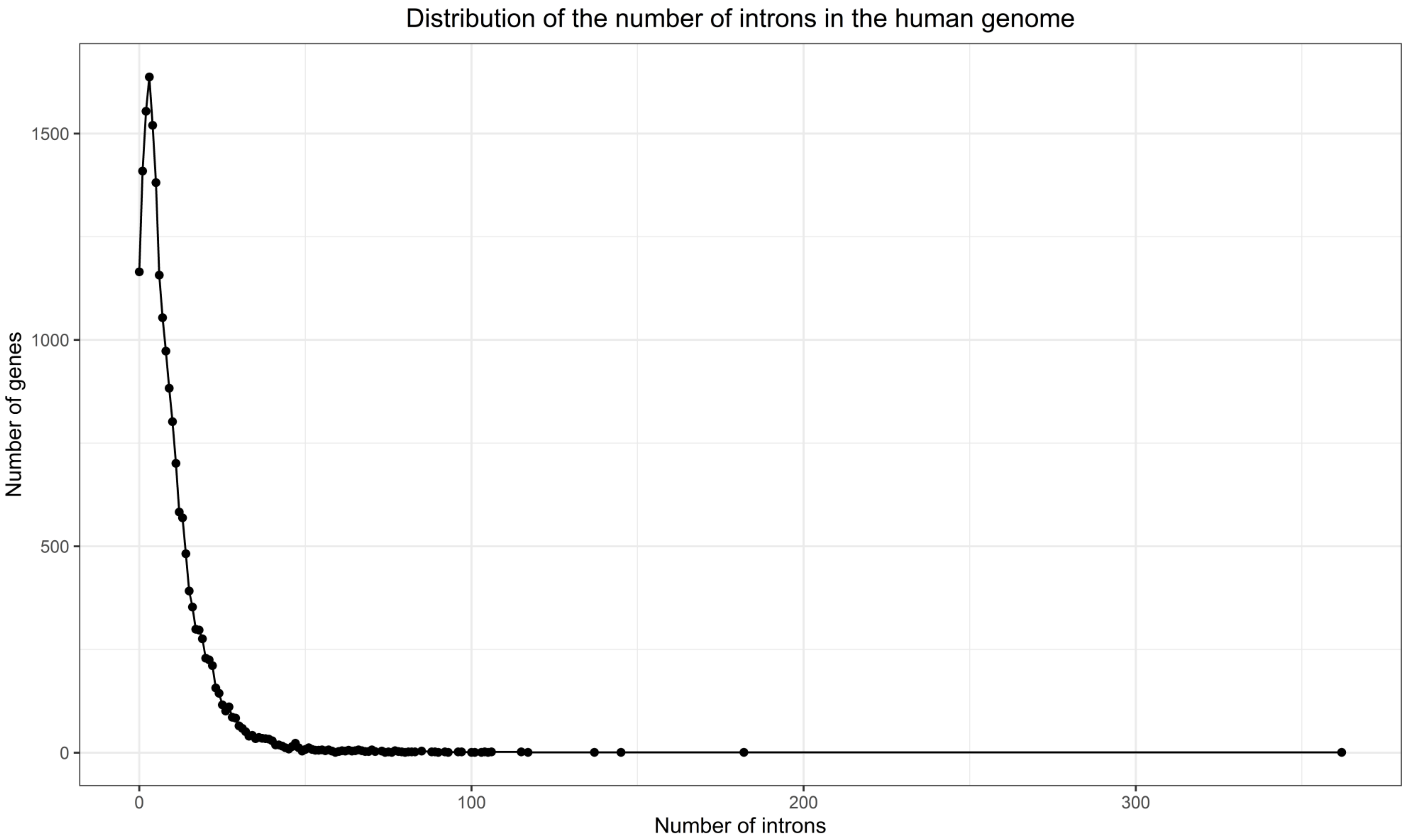

Supplementary Figure 7B.
Distributions of the number of introns in the human genome, for protein-coding genes only. The number of introns was obtained from biomart.
