# Supplementary material for: Gene Size Matters: An Analysis of Gene Length in the Human Genome
Source: Front Genet. 2021 Feb 11;12:559998. doi: 10.3389/fgene.2021.559998 (PMC7905317; doi:10.3389/fgene.2021.559998)
Supplement: Supplementary file 14 [file Data_Sheet_8.pdf]

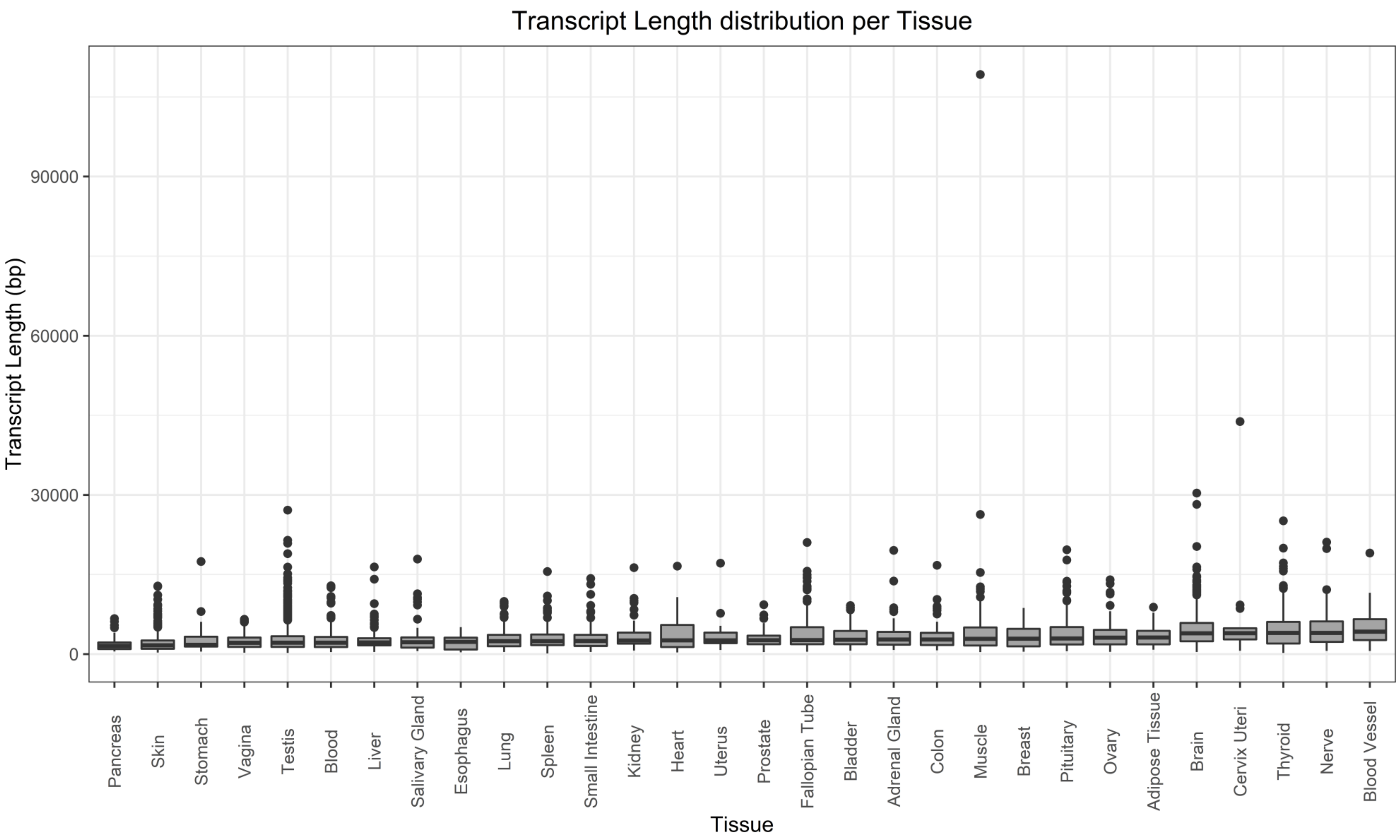

Supplementary Figure 8.

Transcript length distribution for genes specifically expressed in the given Tissues. Tissue specificity was defined as a gene having a Tau specificity score greater than 0.8.
