# Supplementary material for: Gene Size Matters: An Analysis of Gene Length in the Human Genome
Source: Front Genet. 2021 Feb 11;12:559998. doi: 10.3389/fgene.2021.559998 (PMC7905317; doi:10.3389/fgene.2021.559998)
Supplement: Supplementary file 15 [file Data_Sheet_9.pdf]

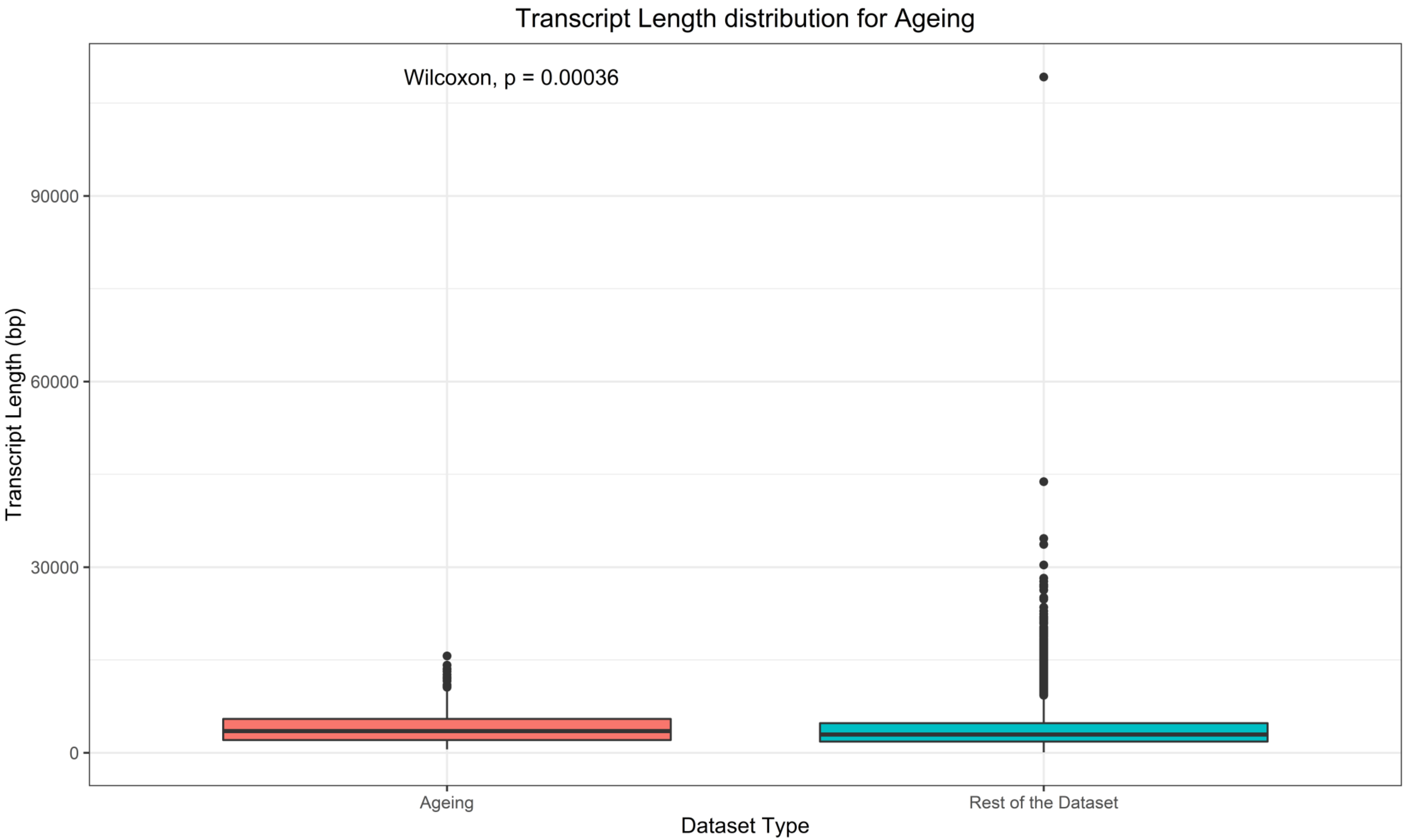

Supplementary Figure 9A.
Transcript length distribution for Ageing related genes and for the rest of the dataset. Ageing related genes were obtained from GenAge and Transcript Length was obtained from biomart.

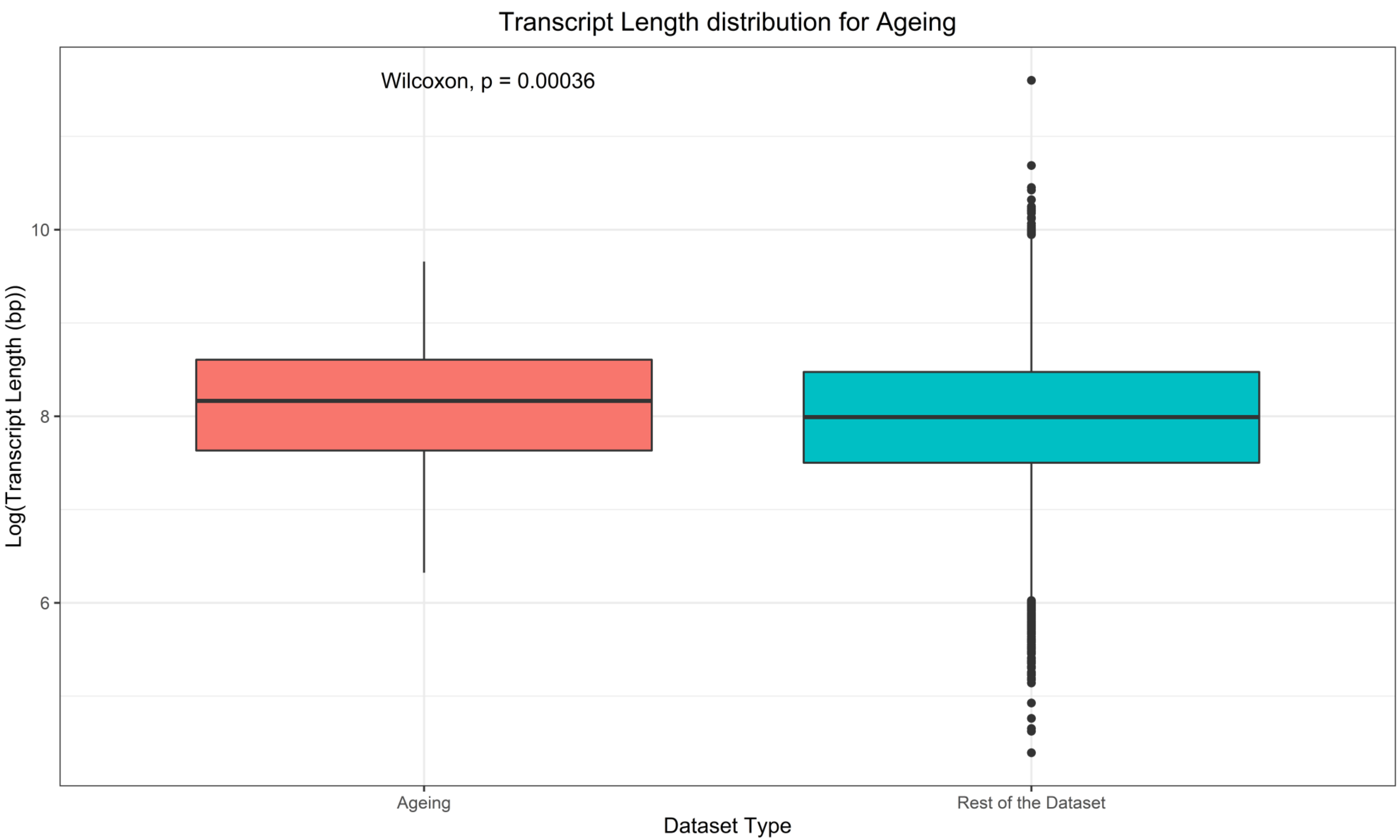

Supplementary Figure 9B.
Log transformed Transcript length distribution for Ageing related genes and for the rest of the dataset. Ageing related genes were obtained from GenAge and Transcript Length was obtained from biomart.

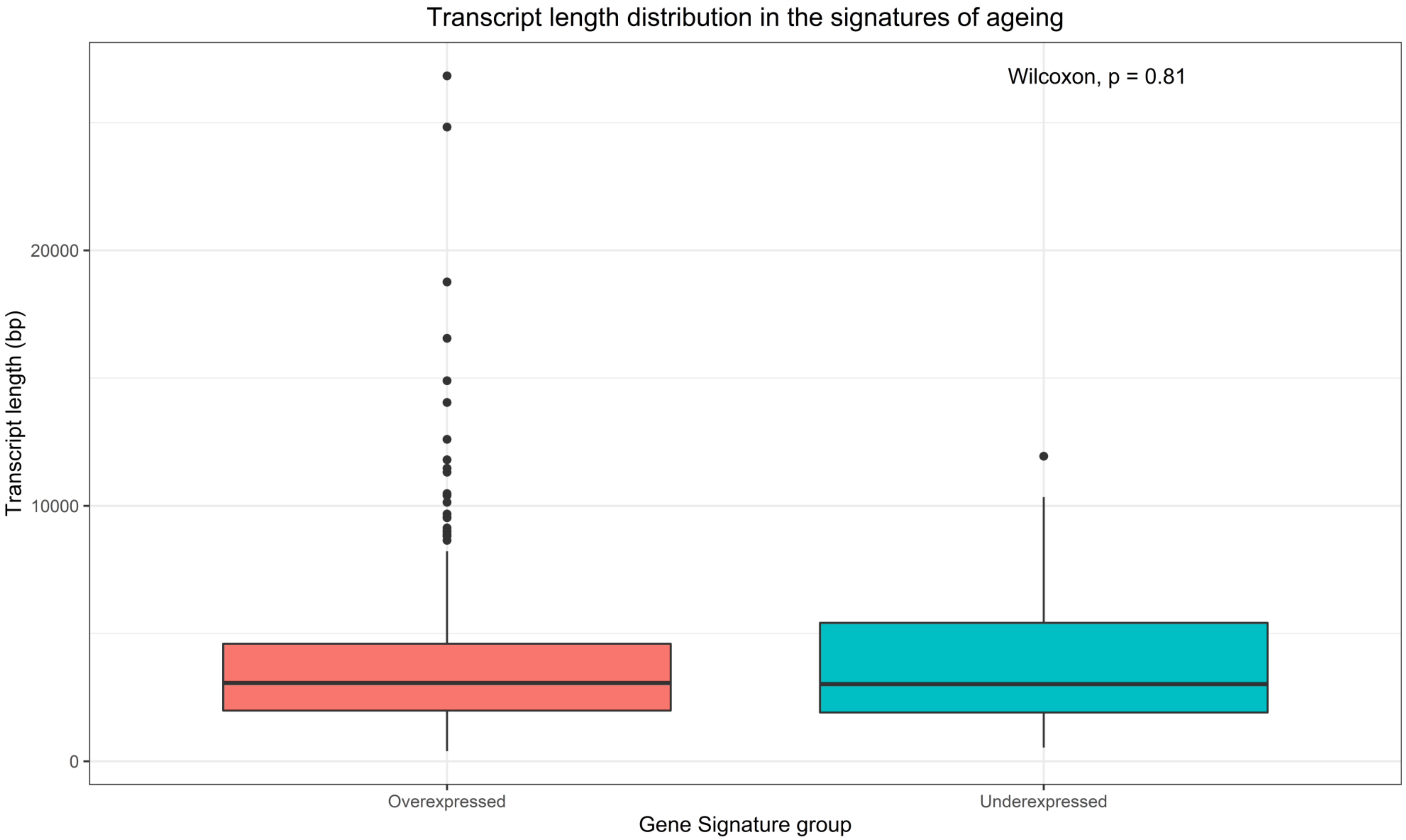

Supplementary Figure 9C.
Transcript length distribution for genes overexpressed and underexpressed with age.

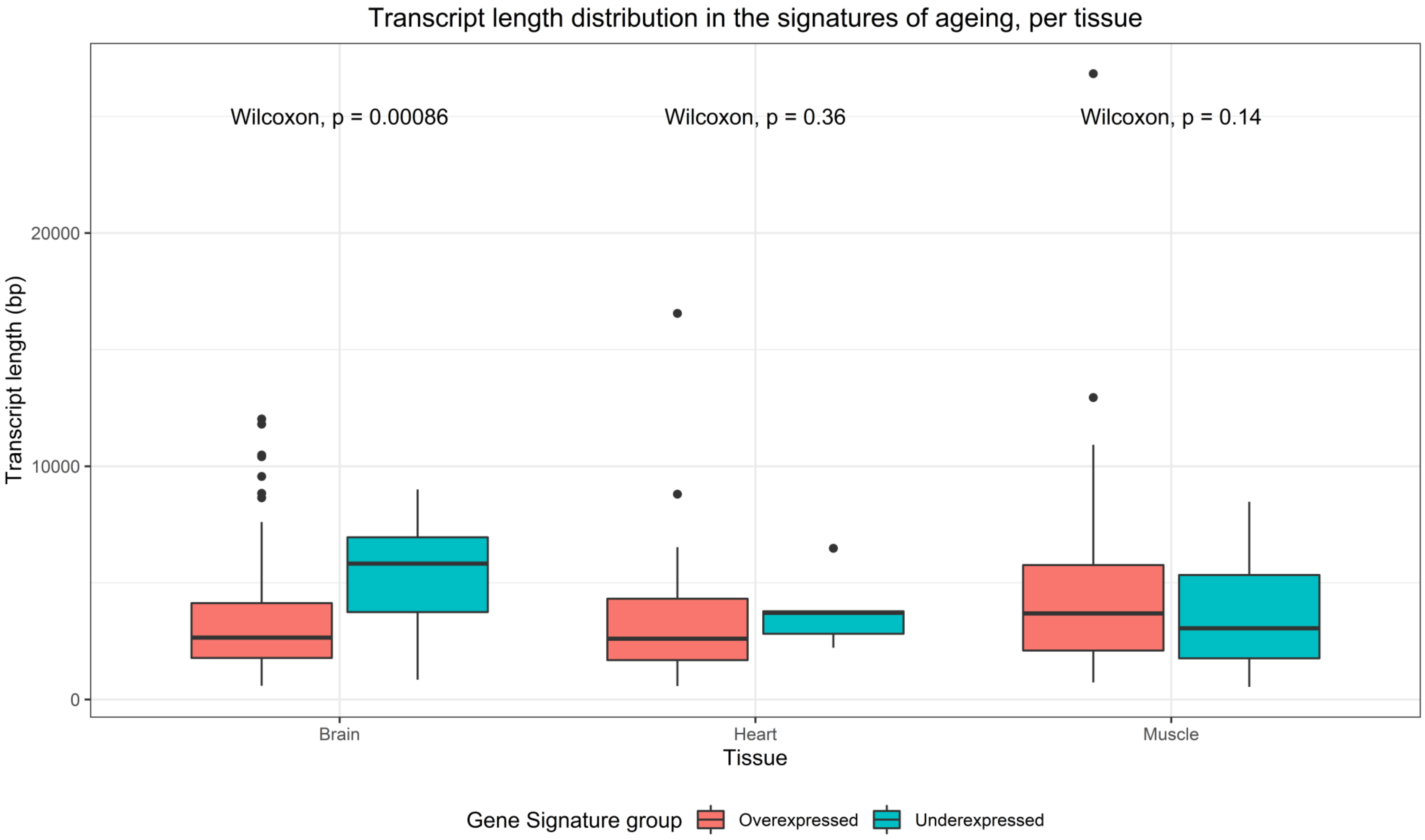

Supplementary Figure 9D.
Transcript length distribution for genes overexpressed and underexpressed with age, for brain, heart and muscle.
