# Supplementary material for: Gene Size Matters: An Analysis of Gene Length in the Human Genome
Source: Front Genet. 2021 Feb 11;12:559998. doi: 10.3389/fgene.2021.559998 (PMC7905317; doi:10.3389/fgene.2021.559998)
Supplement: Supplementary file 16 [file Data_Sheet_10.pdf]

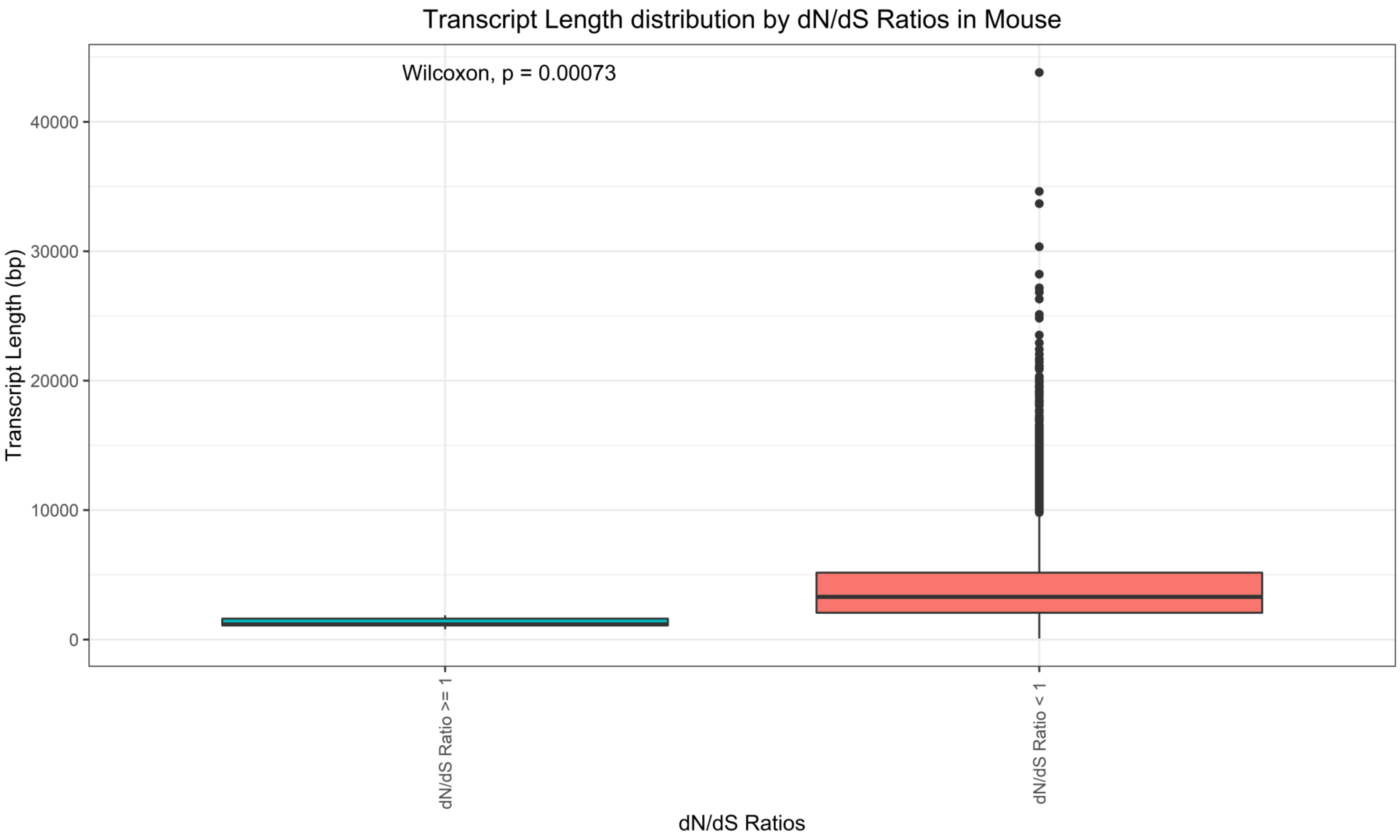

Supplementary Figure 10A.
Transcript length distribution for different dN/dS rations in Mouse. dN values, dS values and Transcript Length for each transcript were obtained using biomart.

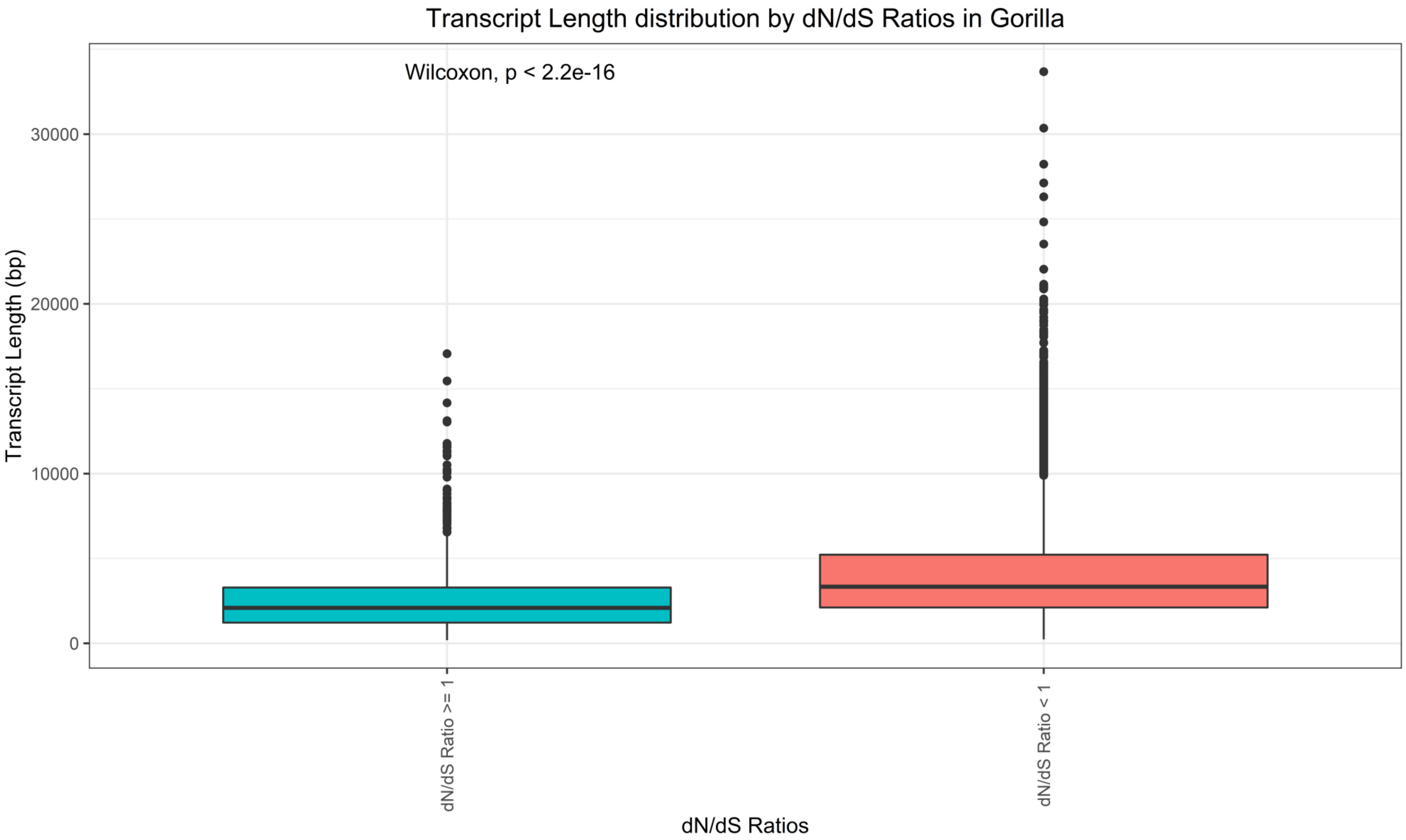

Supplementary Figure 10B.
Transcript length distribution for different dN/dS rations in Gorilla. dN values, dS values and Transcript Length for each transcript were obtained using biomart.

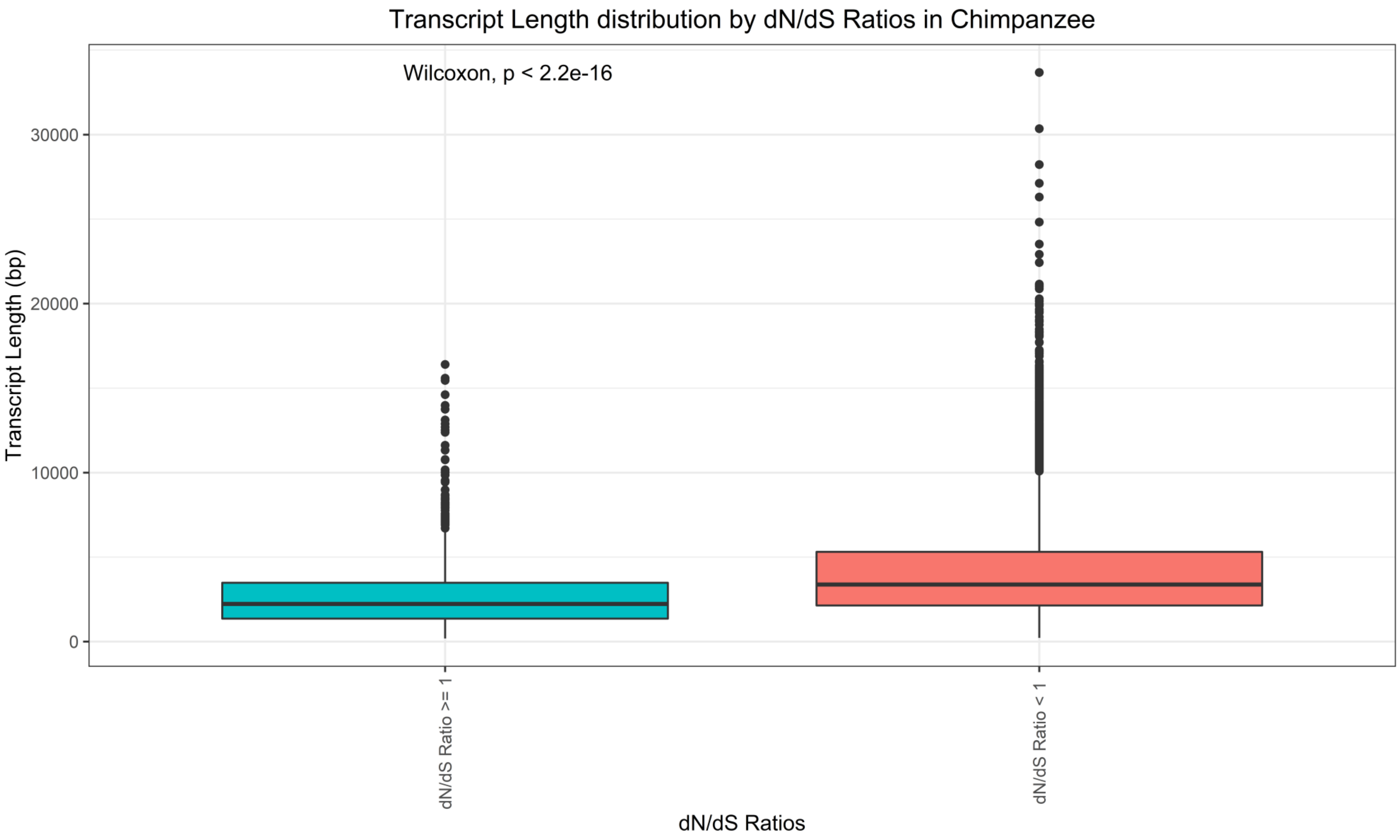

Supplementary Figure 10C.
Transcript length distribution for different dN/dS rations in Chimpanzee. dN values, dS values and Transcript Length for each transcript were obtained using biomart.
