# Supplementary material for: Gene Size Matters: An Analysis of Gene Length in the Human Genome
Source: Front Genet. 2021 Feb 11;12:559998. doi: 10.3389/fgene.2021.559998 (PMC7905317; doi:10.3389/fgene.2021.559998)
Supplement: Supplementary file 17 [file Data_Sheet_11.pdf]

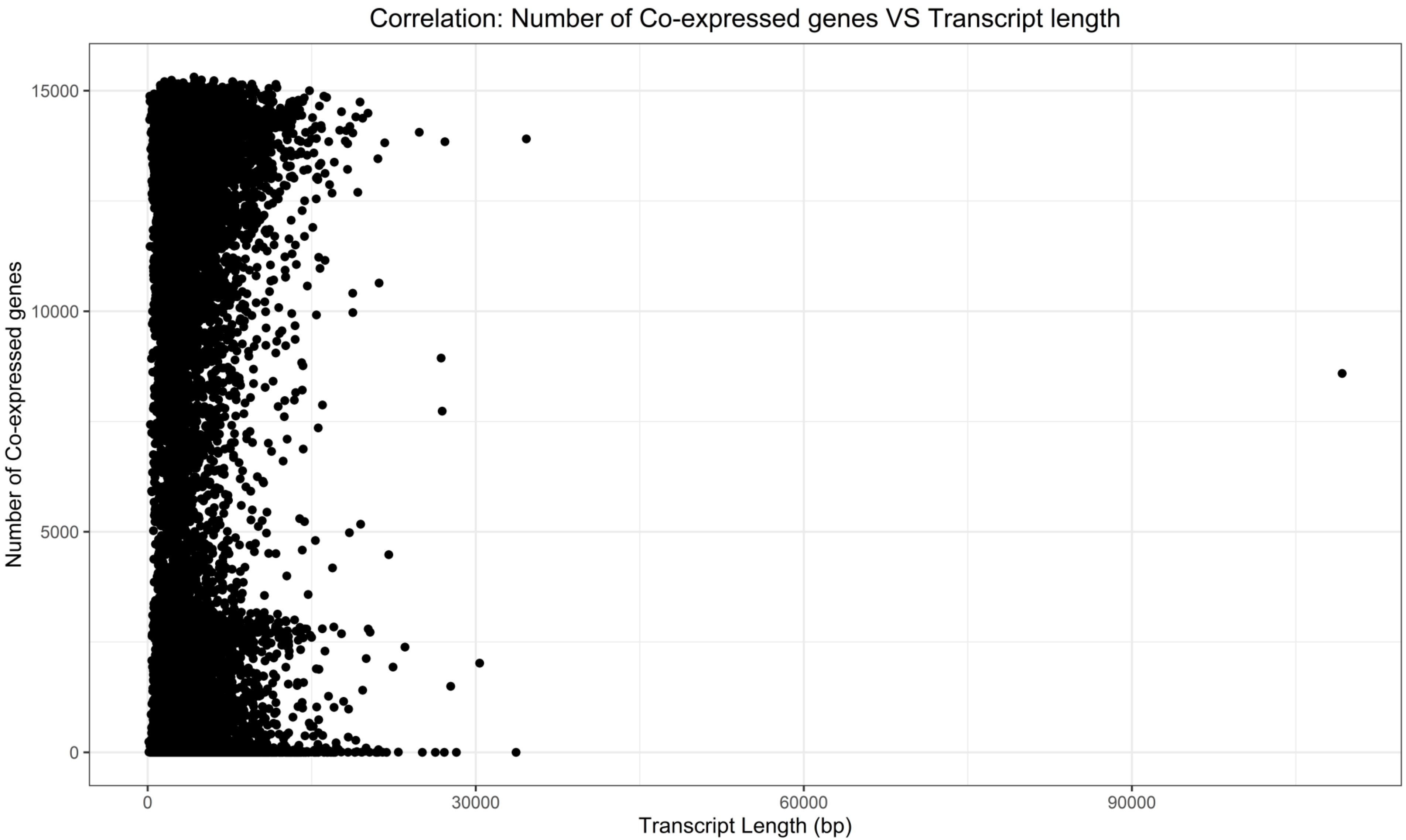

Supplementary Figure 11A.
Correlation between the number of co-expressed genes and Transcript Length (bp) (Kendall test, tau = 0.10, p-value < 2.20E-16). Number of co-expressed genes was obtained from GeneFriends and Transcript Length was obtained from biomart.

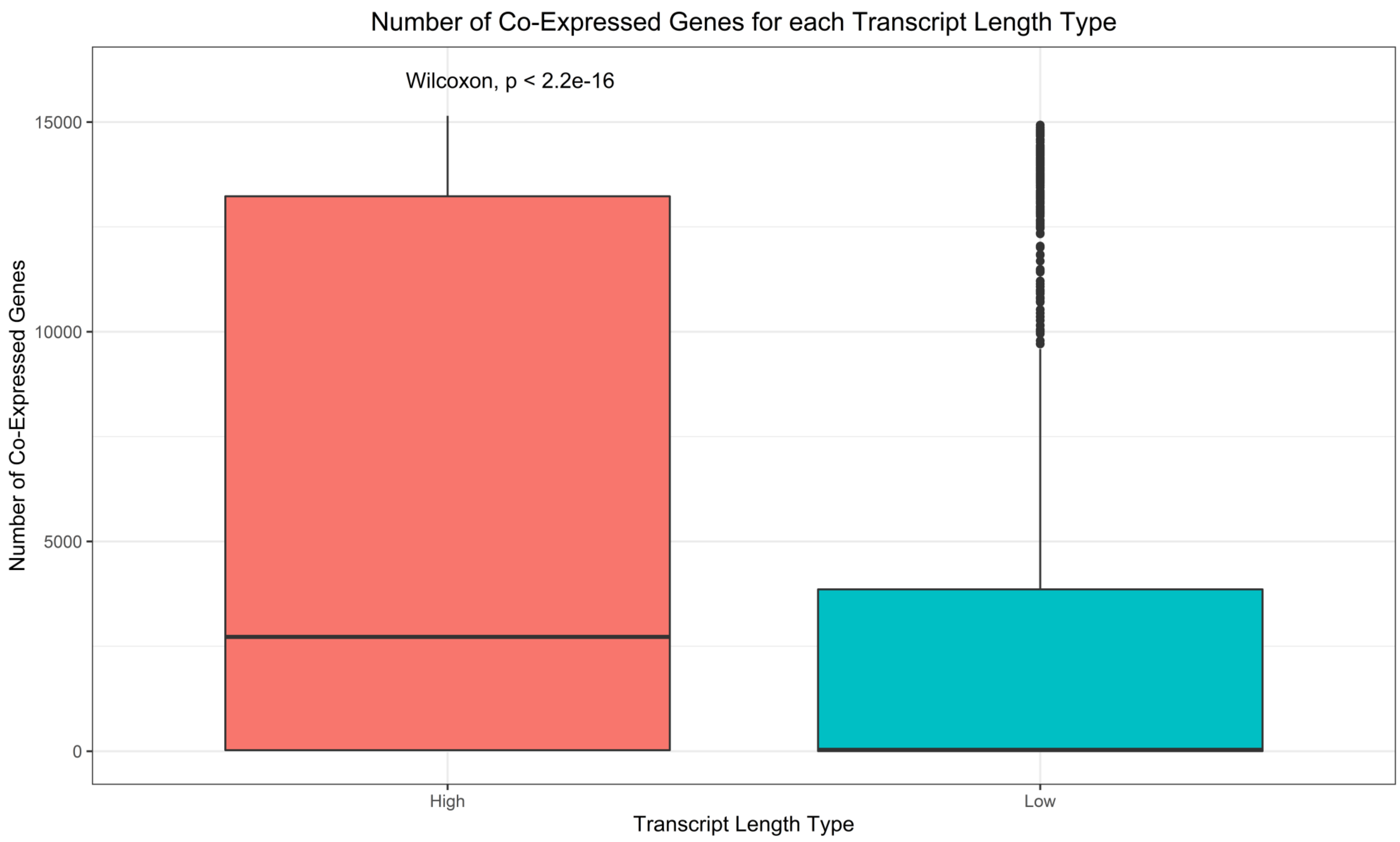

Supplementary Figure 11B.
Distribution of the number of co-expressed genes for long (High) genes and small (Low) genes. Number of co-expressed genes was obtained from GeneFriends and Transcript Length was obtained from biomart.

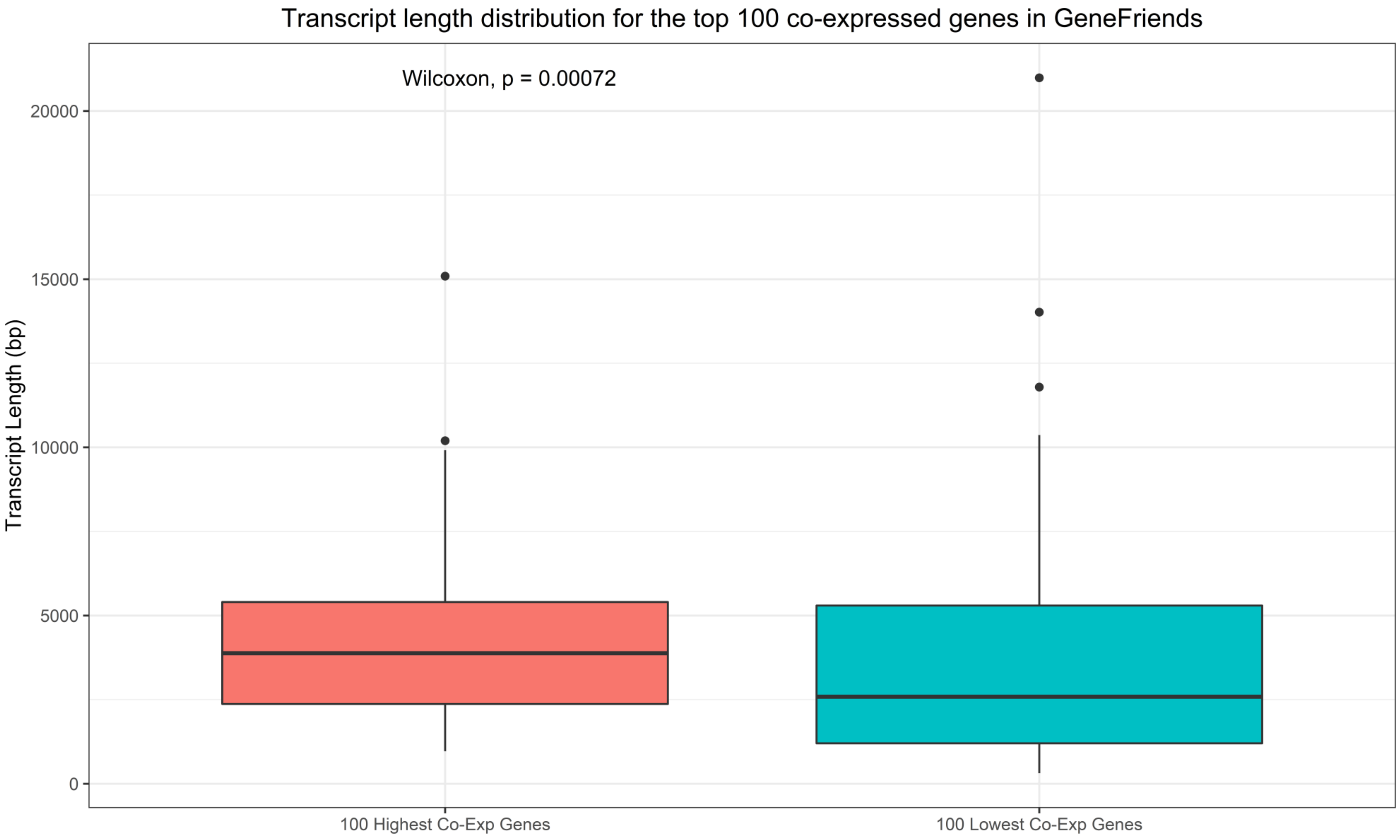

Supplementary Figure 11C.
Distribution of transcript length from our dataset for the top hundred highest/lowest co-expressed genes in GeneFriends. Median co-expression correlation values were calculated using GeneFriends and Transcript Length was obtained from biomart.
