# Supplementary material for: Gene Size Matters: An Analysis of Gene Length in the Human Genome
Source: Front Genet. 2021 Feb 11;12:559998. doi: 10.3389/fgene.2021.559998 (PMC7905317; doi:10.3389/fgene.2021.559998)
Supplement: Supplementary file 18 [file Data_Sheet_12.pdf]

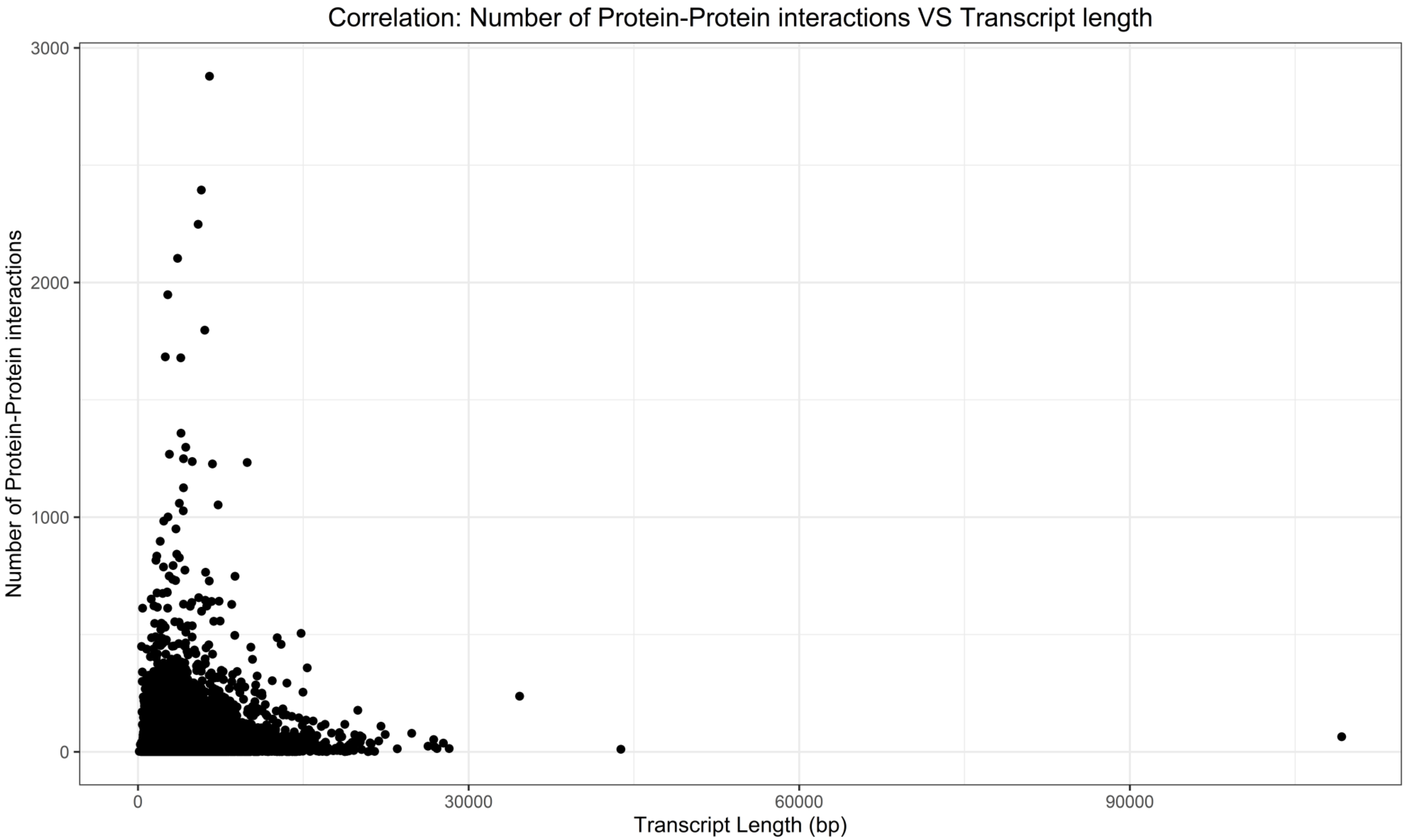

Supplementary Figure 12A.
Correlation between Number of Protein-Protein interactions and Transcript Length (bp) (Kendall test, tau = 0.06, p-value < 2.2E-16). Number of Protein-Protein interactions was obtained from BioGRID and the transcript length was obtained from biomart.

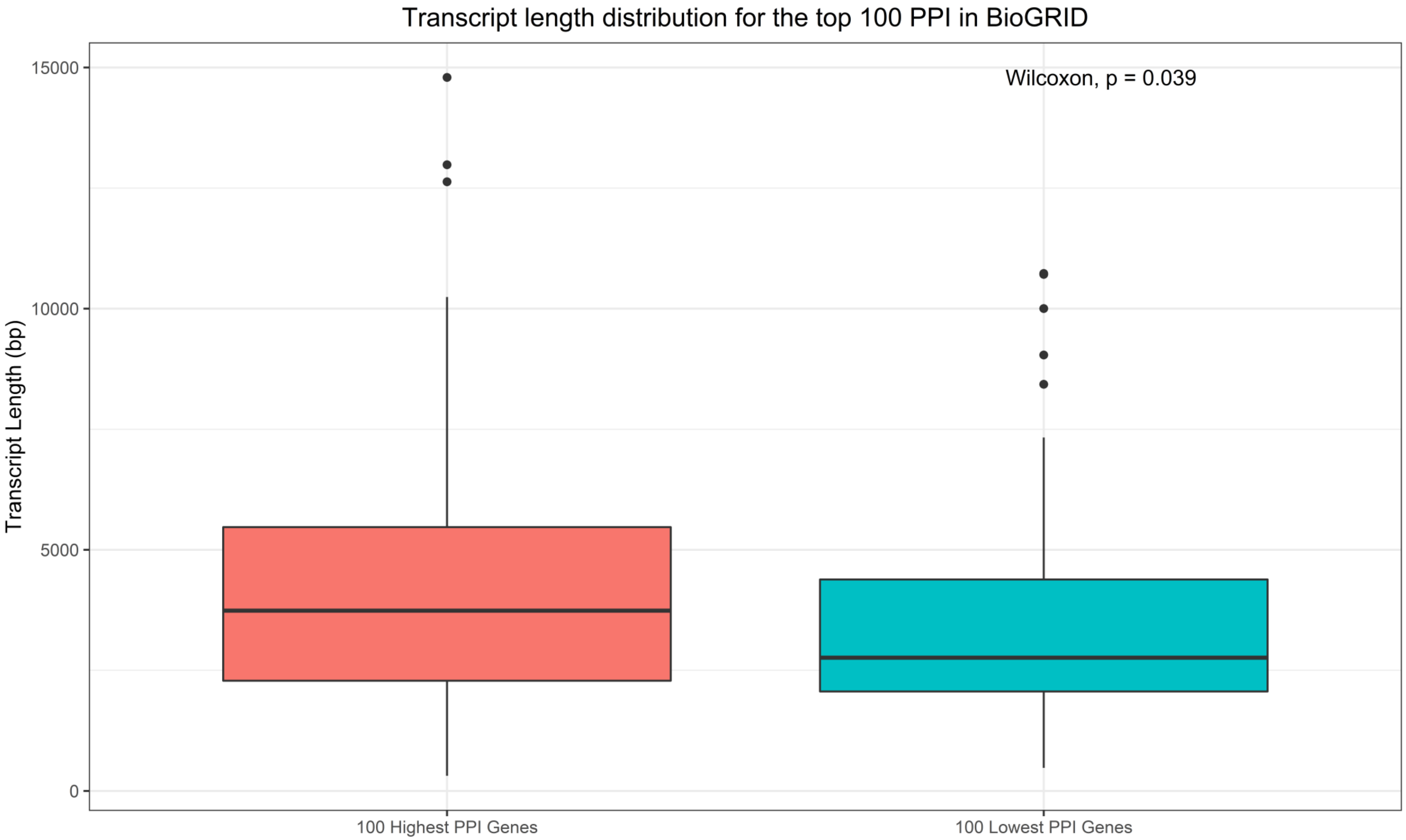

Supplementary Figure 12B.

Distribution of the transcript length for the top 100 genes with the highest and lowest protein-protein interactions. Number of Protein-Protein interactions was obtained from BioGRID and the transcript length was obtained from biomart.
